# Topological cell clustering in the ATLAS calorimeters and its performance in LHC Run 1

**DOI:** 10.1140/epjc/s10052-017-5004-5

**Published:** 2017-07-24

**Authors:** G. Aad, B. Abbott, J. Abdallah, O. Abdinov, R. Aben, M. Abolins, O. S. AbouZeid, H. Abramowicz, H. Abreu, R. Abreu, Y. Abulaiti, B. S. Acharya, L. Adamczyk, D. L. Adams, J. Adelman, S. Adomeit, T. Adye, A. A. Affolder, T. Agatonovic-Jovin, J. Agricola, J. A. Aguilar-Saavedra, S. P. Ahlen, F. Ahmadov, G. Aielli, H. Akerstedt, T. P. A. Åkesson, A. V. Akimov, G. L. Alberghi, J. Albert, S. Albrand, M. J. Alconada Verzini, M. Aleksa, I. N. Aleksandrov, C. Alexa, G. Alexander, T. Alexopoulos, M. Alhroob, G. Alimonti, L. Alio, J. Alison, S. P. Alkire, B. M. M. Allbrooke, P. P. Allport, A. Aloisio, A. Alonso, F. Alonso, C. Alpigiani, A. Altheimer, B. Alvarez Gonzalez, D. Álvarez Piqueras, M. G. Alviggi, B. T. Amadio, K. Amako, Y. Amaral Coutinho, C. Amelung, D. Amidei, S. P. Amor Dos Santos, A. Amorim, S. Amoroso, N. Amram, G. Amundsen, C. Anastopoulos, L. S. Ancu, N. Andari, T. Andeen, C. F. Anders, G. Anders, J. K. Anders, K. J. Anderson, A. Andreazza, V. Andrei, S. Angelidakis, I. Angelozzi, P. Anger, A. Angerami, F. Anghinolfi, A. V. Anisenkov, N. Anjos, A. Annovi, M. Antonelli, A. Antonov, J. Antos, F. Anulli, M. Aoki, L. Aperio Bella, G. Arabidze, Y. Arai, J. P. Araque, A. T. H. Arce, F. A. Arduh, J.-F. Arguin, S. Argyropoulos, M. Arik, A. J. Armbruster, O. Arnaez, H. Arnold, M. Arratia, O. Arslan, A. Artamonov, G. Artoni, S. Artz, S. Asai, N. Asbah, A. Ashkenazi, B. Åsman, L. Asquith, K. Assamagan, R. Astalos, M. Atkinson, N. B. Atlay, K. Augsten, M. Aurousseau, G. Avolio, B. Axen, M. K. Ayoub, G. Azuelos, M. A. Baak, A. E. Baas, M. J. Baca, C. Bacci, H. Bachacou, K. Bachas, M. Backes, M. Backhaus, P. Bagiacchi, P. Bagnaia, Y. Bai, T. Bain, J. T. Baines, O. K. Baker, E. M. Baldin, P. Balek, T. Balestri, F. Balli, W. K. Balunas, E. Banas, Sw. Banerjee, A. A. E. Bannoura, L. Barak, E. L. Barberio, D. Barberis, M. Barbero, T. Barillari, M. Barisonzi, T. Barklow, N. Barlow, S. L. Barnes, B. M. Barnett, R. M. Barnett, Z. Barnovska, A. Baroncelli, G. Barone, A. J. Barr, F. Barreiro, J. Barreiro Guimarães da Costa, R. Bartoldus, A. E. Barton, P. Bartos, A. Basalaev, A. Bassalat, A. Basye, R. L. Bates, S. J. Batista, J. R. Batley, M. Battaglia, M. Bauce, F. Bauer, H. S. Bawa, J. B. Beacham, M. D. Beattie, T. Beau, P. H. Beauchemin, R. Beccherle, P. Bechtle, H. P. Beck, K. Becker, M. Becker, M. Beckingham, C. Becot, A. J. Beddall, A. Beddall, V. A. Bednyakov, C. P. Bee, L. J. Beemster, T. A. Beermann, M. Begel, J. K. Behr, C. Belanger-Champagne, W. H. Bell, G. Bella, L. Bellagamba, A. Bellerive, M. Bellomo, K. Belotskiy, O. Beltramello, O. Benary, D. Benchekroun, M. Bender, K. Bendtz, N. Benekos, Y. Benhammou, E. Benhar Noccioli, J. A. Benitez Garcia, D. P. Benjamin, J. R. Bensinger, S. Bentvelsen, L. Beresford, M. Beretta, D. Berge, E. Bergeaas Kuutmann, N. Berger, F. Berghaus, J. Beringer, C. Bernard, N. R. Bernard, C. Bernius, F. U. Bernlochner, T. Berry, P. Berta, C. Bertella, G. Bertoli, F. Bertolucci, C. Bertsche, D. Bertsche, M. I. Besana, G. J. Besjes, O. Bessidskaia Bylund, M. Bessner, N. Besson, C. Betancourt, S. Bethke, A. J. Bevan, W. Bhimji, R. M. Bianchi, L. Bianchini, M. Bianco, O. Biebel, D. Biedermann, N. V. Biesuz, M. Biglietti, J. Bilbao De Mendizabal, H. Bilokon, M. Bindi, S. Binet, A. Bingul, C. Bini, S. Biondi, D. M. Bjergaard, C. W. Black, J. E. Black, K. M. Black, D. Blackburn, R. E. Blair, J.-B. Blanchard, J. E. Blanco, T. Blazek, I. Bloch, C. Blocker, W. Blum, U. Blumenschein, S. Blunier, G. J. Bobbink, V. S. Bobrovnikov, S. S. Bocchetta, A. Bocci, C. Bock, M. Boehler, J. A. Bogaerts, D. Bogavac, A. G. Bogdanchikov, C. Bohm, V. Boisvert, T. Bold, V. Boldea, A. S. Boldyrev, M. Bomben, M. Bona, M. Boonekamp, A. Borisov, G. Borissov, S. Borroni, J. Bortfeldt, V. Bortolotto, K. Bos, D. Boscherini, M. Bosman, J. Boudreau, J. Bouffard, E. V. Bouhova-Thacker, D. Boumediene, C. Bourdarios, N. Bousson, S. K. Boutle, A. Boveia, J. Boyd, I. R. Boyko, I. Bozic, J. Bracinik, A. Brandt, G. Brandt, O. Brandt, U. Bratzler, B. Brau, J. E. Brau, H. M. Braun, W. D. Breaden Madden, K. Brendlinger, A. J. Brennan, L. Brenner, R. Brenner, S. Bressler, T. M. Bristow, D. Britton, D. Britzger, F. M. Brochu, I. Brock, R. Brock, J. Bronner, G. Brooijmans, T. Brooks, W. K. Brooks, J. Brosamer, E. Brost, P. A. Bruckman de Renstrom, D. Bruncko, R. Bruneliere, A. Bruni, G. Bruni, M. Bruschi, N. Bruscino, L. Bryngemark, T. Buanes, Q. Buat, P. Buchholz, A. G. Buckley, I. A. Budagov, F. Buehrer, L. Bugge, M. K. Bugge, O. Bulekov, D. Bullock, H. Burckhart, S. Burdin, C. D. Burgard, B. Burghgrave, S. Burke, I. Burmeister, E. Busato, D. Büscher, V. Büscher, P. Bussey, J. M. Butler, A. I. Butt, C. M. Buttar, J. M. Butterworth, P. Butti, W. Buttinger, A. Buzatu, A. R. Buzykaev, S. Cabrera Urbán, D. Caforio, V. M. Cairo, O. Cakir, N. Calace, P. Calafiura, A. Calandri, G. Calderini, P. Calfayan, L. P. Caloba, D. Calvet, S. Calvet, R. Camacho Toro, S. Camarda, P. Camarri, D. Cameron, R. Caminal Armadans, S. Campana, M. Campanelli, A. Campoverde, V. Canale, A. Canepa, M. Cano Bret, J. Cantero, R. Cantrill, T. Cao, M. D. M. Capeans Garrido, I. Caprini, M. Caprini, M. Capua, R. Caputo, R. M. Carbone, R. Cardarelli, F. Cardillo, T. Carli, G. Carlino, L. Carminati, S. Caron, E. Carquin, G. D. Carrillo-Montoya, J. R. Carter, J. Carvalho, D. Casadei, M. P. Casado, M. Casolino, D. W. Casper, E. Castaneda-Miranda, A. Castelli, V. Castillo Gimenez, N. F. Castro, P. Catastini, A. Catinaccio, J. R. Catmore, A. Cattai, J. Caudron, V. Cavaliere, D. Cavalli, M. Cavalli-Sforza, V. Cavasinni, F. Ceradini, L. Cerda Alberich, B. C. Cerio, K. Cerny, A. S. Cerqueira, A. Cerri, L. Cerrito, F. Cerutti, M. Cerv, A. Cervelli, S. A. Cetin, A. Chafaq, D. Chakraborty, I. Chalupkova, Y. L. Chan, P. Chang, J. D. Chapman, D. G. Charlton, C. C. Chau, C. A. Chavez Barajas, S. Che, S. Cheatham, A. Chegwidden, S. Chekanov, S. V. Chekulaev, G. A. Chelkov, M. A. Chelstowska, C. Chen, H. Chen, K. Chen, L. Chen, S. Chen, S. Chen, X. Chen, Y. Chen, H. C. Cheng, Y. Cheng, A. Cheplakov, E. Cheremushkina, R. Cherkaoui El Moursli, V. Chernyatin, E. Cheu, L. Chevalier, V. Chiarella, G. Chiarelli, G. Chiodini, A. S. Chisholm, R. T. Chislett, A. Chitan, M. V. Chizhov, K. Choi, S. Chouridou, B. K. B. Chow, V. Christodoulou, D. Chromek-Burckhart, J. Chudoba, A. J. Chuinard, J. J. Chwastowski, L. Chytka, G. Ciapetti, A. K. Ciftci, D. Cinca, V. Cindro, I. A. Cioara, A. Ciocio, F. Cirotto, Z. H. Citron, M. Ciubancan, A. Clark, B. L. Clark, P. J. Clark, R. N. Clarke, C. Clement, Y. Coadou, M. Cobal, A. Coccaro, J. Cochran, L. Coffey, J. G. Cogan, L. Colasurdo, B. Cole, S. Cole, A. P. Colijn, J. Collot, T. Colombo, G. Compostella, P. Conde Muiño, E. Coniavitis, S. H. Connell, I. A. Connelly, V. Consorti, S. Constantinescu, C. Conta, G. Conti, F. Conventi, M. Cooke, B. D. Cooper, A. M. Cooper-Sarkar, T. Cornelissen, M. Corradi, F. Corriveau, A. Corso-Radu, A. Cortes-Gonzalez, G. Cortiana, G. Costa, M. J. Costa, D. Costanzo, D. Côté, G. Cottin, G. Cowan, B. E. Cox, K. Cranmer, G. Cree, S. Crépé-Renaudin, F. Crescioli, W. A. Cribbs, M. Crispin Ortuzar, M. Cristinziani, V. Croft, G. Crosetti, T. Cuhadar Donszelmann, J. Cummings, M. Curatolo, J. Cúth, C. Cuthbert, H. Czirr, P. Czodrowski, S. D’Auria, M. D’Onofrio, M. J. Da Cunha Sargedas De Sousa, C. Da Via, W. Dabrowski, A. Dafinca, T. Dai, O. Dale, F. Dallaire, C. Dallapiccola, M. Dam, J. R. Dandoy, N. P. Dang, A. C. Daniells, M. Danninger, M. Dano Hoffmann, V. Dao, G. Darbo, S. Darmora, J. Dassoulas, A. Dattagupta, W. Davey, C. David, T. Davidek, E. Davies, M. Davies, P. Davison, Y. Davygora, E. Dawe, I. Dawson, R. K. Daya-Ishmukhametova, K. De, R. de Asmundis, A. De Benedetti, S. De Castro, S. De Cecco, N. De Groot, P. de Jong, H. De la Torre, F. De Lorenzi, D. De Pedis, A. De Salvo, U. De Sanctis, A. De Santo, J. B. De Vivie De Regie, W. J. Dearnaley, R. Debbe, C. Debenedetti, D. V. Dedovich, I. Deigaard, J. Del Peso, T. Del Prete, D. Delgove, F. Deliot, C. M. Delitzsch, M. Deliyergiyev, A. Dell’Acqua, L. Dell’Asta, M. Dell’Orso, M. Della Pietra, D. della Volpe, M. Delmastro, P. A. Delsart, C. Deluca, D. A. DeMarco, S. Demers, M. Demichev, A. Demilly, S. P. Denisov, D. Derendarz, J. E. Derkaoui, F. Derue, P. Dervan, K. Desch, C. Deterre, K. Dette, P. O. Deviveiros, A. Dewhurst, S. Dhaliwal, A. Di Ciaccio, L. Di Ciaccio, A. Di Domenico, C. Di Donato, A. Di Girolamo, B. Di Girolamo, A. Di Mattia, B. Di Micco, R. Di Nardo, A. Di Simone, R. Di Sipio, D. Di Valentino, C. Diaconu, M. Diamond, F. A. Dias, M. A. Diaz, E. B. Diehl, J. Dietrich, S. Diglio, A. Dimitrievska, J. Dingfelder, P. Dita, S. Dita, F. Dittus, F. Djama, T. Djobava, J. I. Djuvsland, M. A. B. do Vale, D. Dobos, M. Dobre, C. Doglioni, T. Dohmae, J. Dolejsi, Z. Dolezal, B. A. Dolgoshein, M. Donadelli, S. Donati, P. Dondero, J. Donini, J. Dopke, A. Doria, M. T. Dova, A. T. Doyle, E. Drechsler, M. Dris, Y. Du, E. Dubreuil, E. Duchovni, G. Duckeck, O. A. Ducu, D. Duda, A. Dudarev, L. Duflot, L. Duguid, M. Dührssen, M. Dunford, H. Duran Yildiz, M. Düren, A. Durglishvili, D. Duschinger, B. Dutta, M. Dyndal, C. Eckardt, K. M. Ecker, R. C. Edgar, W. Edson, N. C. Edwards, W. Ehrenfeld, T. Eifert, G. Eigen, K. Einsweiler, T. Ekelof, M. El Kacimi, M. Ellert, S. Elles, F. Ellinghaus, A. A. Elliot, N. Ellis, J. Elmsheuser, M. Elsing, D. Emeliyanov, Y. Enari, O. C. Endner, M. Endo, J. Erdmann, A. Ereditato, G. Ernis, J. Ernst, M. Ernst, S. Errede, E. Ertel, M. Escalier, H. Esch, C. Escobar, B. Esposito, A. I. Etienvre, E. Etzion, H. Evans, A. Ezhilov, L. Fabbri, G. Facini, R. M. Fakhrutdinov, S. Falciano, R. J. Falla, J. Faltova, Y. Fang, M. Fanti, A. Farbin, A. Farilla, T. Farooque, S. Farrell, S. M. Farrington, P. Farthouat, F. Fassi, P. Fassnacht, D. Fassouliotis, M. Faucci Giannelli, A. Favareto, L. Fayard, O. L. Fedin, W. Fedorko, S. Feigl, L. Feligioni, C. Feng, E. J. Feng, H. Feng, A. B. Fenyuk, L. Feremenga, P. Fernandez Martinez, S. Fernandez Perez, J. Ferrando, A. Ferrari, P. Ferrari, R. Ferrari, D. E. Ferreira de Lima, A. Ferrer, D. Ferrere, C. Ferretti, A. Ferretto Parodi, M. Fiascaris, F. Fiedler, A. Filipčič, M. Filipuzzi, F. Filthaut, M. Fincke-Keeler, K. D. Finelli, M. C. N. Fiolhais, L. Fiorini, A. Firan, A. Fischer, C. Fischer, J. Fischer, W. C. Fisher, N. Flaschel, I. Fleck, P. Fleischmann, G. T. Fletcher, G. Fletcher, R. R. M. Fletcher, T. Flick, A. Floderus, L. R. Flores Castillo, M. J. Flowerdew, A. Formica, A. Forti, D. Fournier, H. Fox, S. Fracchia, P. Francavilla, M. Franchini, D. Francis, L. Franconi, M. Franklin, M. Frate, M. Fraternali, D. Freeborn, S. T. French, S. M. Fressard-Batraneanu, F. Friedrich, D. Froidevaux, J. A. Frost, C. Fukunaga, E. Fullana Torregrosa, B. G. Fulsom, T. Fusayasu, J. Fuster, C. Gabaldon, O. Gabizon, A. Gabrielli, A. Gabrielli, G. P. Gach, S. Gadatsch, S. Gadomski, G. Gagliardi, P. Gagnon, C. Galea, B. Galhardo, E. J. Gallas, B. J. Gallop, P. Gallus, G. Galster, K. K. Gan, J. Gao, Y. Gao, Y. S. Gao, F. M. Garay Walls, F. Garberson, C. García, J. E. García Navarro, M. Garcia-Sciveres, R. W. Gardner, N. Garelli, V. Garonne, C. Gatti, A. Gaudiello, G. Gaudio, B. Gaur, L. Gauthier, P. Gauzzi, I. L. Gavrilenko, C. Gay, G. Gaycken, E. N. Gazis, P. Ge, Z. Gecse, C. N. P. Gee, Ch. Geich-Gimbel, M. P. Geisler, C. Gemme, M. H. Genest, C. Geng, S. Gentile, M. George, S. George, D. Gerbaudo, A. Gershon, S. Ghasemi, H. Ghazlane, B. Giacobbe, S. Giagu, V. Giangiobbe, P. Giannetti, B. Gibbard, S. M. Gibson, M. Gignac, M. Gilchriese, T. P. S. Gillam, D. Gillberg, G. Gilles, D. M. Gingrich, N. Giokaris, M. P. Giordani, F. M. Giorgi, F. M. Giorgi, P. F. Giraud, P. Giromini, D. Giugni, C. Giuliani, M. Giulini, B. K. Gjelsten, S. Gkaitatzis, I. Gkialas, E. L. Gkougkousis, L. K. Gladilin, C. Glasman, J. Glatzer, P. C. F. Glaysher, A. Glazov, M. Goblirsch-Kolb, J. R. Goddard, J. Godlewski, S. Goldfarb, T. Golling, D. Golubkov, A. Gomes, R. Gonçalo, J. Goncalves Pinto Firmino Da Costa, L. Gonella, S. González de la Hoz, G. Gonzalez Parra, S. Gonzalez-Sevilla, L. Goossens, P. A. Gorbounov, H. A. Gordon, I. Gorelov, B. Gorini, E. Gorini, A. Gorišek, E. Gornicki, A. T. Goshaw, C. Gössling, M. I. Gostkin, D. Goujdami, A. G. Goussiou, N. Govender, E. Gozani, H. M. X. Grabas, L. Graber, I. Grabowska-Bold, P. O. J. Gradin, P. Grafström, J. Gramling, E. Gramstad, S. Grancagnolo, V. Gratchev, H. M. Gray, E. Graziani, Z. D. Greenwood, C. Grefe, K. Gregersen, I. M. Gregor, P. Grenier, J. Griffiths, A. A. Grillo, K. Grimm, S. Grinstein, Ph. Gris, J.-F. Grivaz, S. Groh, J. P. Grohs, A. Grohsjean, E. Gross, J. Grosse-Knetter, G. C. Grossi, Z. J. Grout, L. Guan, J. Guenther, F. Guescini, D. Guest, O. Gueta, E. Guido, T. Guillemin, S. Guindon, U. Gul, C. Gumpert, J. Guo, Y. Guo, S. Gupta, G. Gustavino, P. Gutierrez, N. G. Gutierrez Ortiz, C. Gutschow, C. Guyot, C. Gwenlan, C. B. Gwilliam, A. Haas, C. Haber, H. K. Hadavand, N. Haddad, P. Haefner, S. Hageböck, Z. Hajduk, H. Hakobyan, M. Haleem, J. Haley, D. Hall, G. Halladjian, G. D. Hallewell, K. Hamacher, P. Hamal, K. Hamano, A. Hamilton, G. N. Hamity, P. G. Hamnett, L. Han, K. Hanagaki, K. Hanawa, M. Hance, B. Haney, P. Hanke, R. Hanna, J. B. Hansen, J. D. Hansen, M. C. Hansen, P. H. Hansen, K. Hara, A. S. Hard, T. Harenberg, F. Hariri, S. Harkusha, R. D. Harrington, P. F. Harrison, F. Hartjes, M. Hasegawa, Y. Hasegawa, A. Hasib, S. Hassani, S. Haug, R. Hauser, L. Hauswald, M. Havranek, C. M. Hawkes, R. J. Hawkings, A. D. Hawkins, T. Hayashi, D. Hayden, C. P. Hays, J. M. Hays, H. S. Hayward, S. J. Haywood, S. J. Head, T. Heck, V. Hedberg, L. Heelan, S. Heim, T. Heim, B. Heinemann, L. Heinrich, J. Hejbal, L. Helary, S. Hellman, C. Helsens, J. Henderson, R. C. W. Henderson, Y. Heng, C. Hengler, S. Henkelmann, A. Henrichs, A. M. Henriques Correia, S. Henrot-Versille, G. H. Herbert, Y. Hernández Jiménez, G. Herten, R. Hertenberger, L. Hervas, G. G. Hesketh, N. P. Hessey, J. W. Hetherly, R. Hickling, E. Higón-Rodriguez, E. Hill, J. C. Hill, K. H. Hiller, S. J. Hillier, I. Hinchliffe, E. Hines, R. R. Hinman, M. Hirose, D. Hirschbuehl, J. Hobbs, N. Hod, M. C. Hodgkinson, P. Hodgson, A. Hoecker, M. R. Hoeferkamp, F. Hoenig, M. Hohlfeld, D. Hohn, T. R. Holmes, M. Homann, T. M. Hong, W. H. Hopkins, Y. Horii, A. J. Horton, J.-Y. Hostachy, S. Hou, A. Hoummada, J. Howard, J. Howarth, M. Hrabovsky, I. Hristova, J. Hrivnac, T. Hryn’ova, A. Hrynevich, C. Hsu, P. J. Hsu, S.-C. Hsu, D. Hu, Q. Hu, X. Hu, Y. Huang, Z. Hubacek, F. Hubaut, F. Huegging, T. B. Huffman, E. W. Hughes, G. Hughes, M. Huhtinen, T. A. Hülsing, N. Huseynov, J. Huston, J. Huth, G. Iacobucci, G. Iakovidis, I. Ibragimov, L. Iconomidou-Fayard, E. Ideal, Z. Idrissi, P. Iengo, O. Igonkina, T. Iizawa, Y. Ikegami, M. Ikeno, Y. Ilchenko, D. Iliadis, N. Ilic, T. Ince, G. Introzzi, P. Ioannou, M. Iodice, K. Iordanidou, V. Ippolito, A. Irles Quiles, C. Isaksson, M. Ishino, M. Ishitsuka, R. Ishmukhametov, C. Issever, S. Istin, J. M. Iturbe Ponce, R. Iuppa, J. Ivarsson, W. Iwanski, H. Iwasaki, J. M. Izen, V. Izzo, S. Jabbar, B. Jackson, M. Jackson, P. Jackson, M. R. Jaekel, V. Jain, K. B. Jakobi, K. Jakobs, S. Jakobsen, T. Jakoubek, J. Jakubek, D. O. Jamin, D. K. Jana, E. Jansen, R. Jansky, J. Janssen, M. Janus, G. Jarlskog, N. Javadov, T. Javůrek, L. Jeanty, J. Jejelava, G.-Y. Jeng, D. Jennens, P. Jenni, J. Jentzsch, C. Jeske, S. Jézéquel, H. Ji, J. Jia, H. Jiang, Y. Jiang, S. Jiggins, J. Jimenez Pena, S. Jin, A. Jinaru, O. Jinnouchi, M. D. Joergensen, P. Johansson, K. A. Johns, W. J. Johnson, K. Jon-And, G. Jones, R. W. L. Jones, T. J. Jones, J. Jongmanns, P. M. Jorge, K. D. Joshi, J. Jovicevic, X. Ju, A. Juste Rozas, M. Kaci, A. Kaczmarska, M. Kado, H. Kagan, M. Kagan, S. J. Kahn, E. Kajomovitz, C. W. Kalderon, A. Kaluza, S. Kama, A. Kamenshchikov, N. Kanaya, S. Kaneti, V. A. Kantserov, J. Kanzaki, B. Kaplan, L. S. Kaplan, A. Kapliy, D. Kar, K. Karakostas, A. Karamaoun, N. Karastathis, M. J. Kareem, E. Karentzos, M. Karnevskiy, S. N. Karpov, Z. M. Karpova, K. Karthik, V. Kartvelishvili, A. N. Karyukhin, K. Kasahara, L. Kashif, R. D. Kass, A. Kastanas, Y. Kataoka, C. Kato, A. Katre, J. Katzy, K. Kawade, K. Kawagoe, T. Kawamoto, G. Kawamura, S. Kazama, V. F. Kazanin, R. Keeler, R. Kehoe, J. S. Keller, J. J. Kempster, H. Keoshkerian, O. Kepka, B. P. Kerševan, S. Kersten, R. A. Keyes, F. Khalil-zada, H. Khandanyan, A. Khanov, A. G. Kharlamov, T. J. Khoo, V. Khovanskiy, E. Khramov, J. Khubua, S. Kido, H. Y. Kim, S. H. Kim, Y. K. Kim, N. Kimura, O. M. Kind, B. T. King, M. King, S. B. King, J. Kirk, A. E. Kiryunin, T. Kishimoto, D. Kisielewska, F. Kiss, K. Kiuchi, O. Kivernyk, E. Kladiva, M. H. Klein, M. Klein, U. Klein, K. Kleinknecht, P. Klimek, A. Klimentov, R. Klingenberg, J. A. Klinger, T. Klioutchnikova, E.-E. Kluge, P. Kluit, S. Kluth, J. Knapik, E. Kneringer, E. B. F. G. Knoops, A. Knue, A. Kobayashi, D. Kobayashi, T. Kobayashi, M. Kobel, M. Kocian, P. Kodys, T. Koffas, E. Koffeman, L. A. Kogan, S. Kohlmann, Z. Kohout, T. Kohriki, T. Koi, H. Kolanoski, M. Kolb, I. Koletsou, A. A. Komar, Y. Komori, T. Kondo, N. Kondrashova, K. Köneke, A. C. König, T. Kono, R. Konoplich, N. Konstantinidis, R. Kopeliansky, S. Koperny, L. Köpke, A. K. Kopp, K. Korcyl, K. Kordas, A. Korn, A. A. Korol, I. Korolkov, E. V. Korolkova, O. Kortner, S. Kortner, T. Kosek, V. V. Kostyukhin, V. M. Kotov, A. Kotwal, A. Kourkoumeli-Charalampidi, C. Kourkoumelis, V. Kouskoura, A. Koutsman, R. Kowalewski, T. Z. Kowalski, W. Kozanecki, A. S. Kozhin, V. A. Kramarenko, G. Kramberger, D. Krasnopevtsev, M. W. Krasny, A. Krasznahorkay, J. K. Kraus, A. Kravchenko, S. Kreiss, M. Kretz, J. Kretzschmar, K. Kreutzfeldt, P. Krieger, K. Krizka, K. Kroeninger, H. Kroha, J. Kroll, J. Kroseberg, J. Krstic, U. Kruchonak, H. Krüger, N. Krumnack, A. Kruse, M. C. Kruse, M. Kruskal, T. Kubota, H. Kucuk, S. Kuday, S. Kuehn, A. Kugel, F. Kuger, A. Kuhl, T. Kuhl, V. Kukhtin, R. Kukla, Y. Kulchitsky, S. Kuleshov, M. Kuna, T. Kunigo, A. Kupco, H. Kurashige, Y. A. Kurochkin, V. Kus, E. S. Kuwertz, M. Kuze, J. Kvita, T. Kwan, D. Kyriazopoulos, A. La Rosa, J. L. La Rosa Navarro, L. La Rotonda, C. Lacasta, F. Lacava, J. Lacey, H. Lacker, D. Lacour, V. R. Lacuesta, E. Ladygin, R. Lafaye, B. Laforge, T. Lagouri, S. Lai, L. Lambourne, S. Lammers, C. L. Lampen, W. Lampl, E. Lançon, U. Landgraf, M. P. J. Landon, V. S. Lang, J. C. Lange, A. J. Lankford, F. Lanni, K. Lantzsch, A. Lanza, S. Laplace, C. Lapoire, J. F. Laporte, T. Lari, F. Lasagni Manghi, M. Lassnig, P. Laurelli, W. Lavrijsen, A. T. Law, P. Laycock, T. Lazovich, O. Le Dortz, E. Le Guirriec, E. Le Menedeu, M. LeBlanc, T. LeCompte, F. Ledroit-Guillon, C. A. Lee, S. C. Lee, L. Lee, G. Lefebvre, M. Lefebvre, F. Legger, C. Leggett, A. Lehan, G. Lehmann Miotto, X. Lei, W. A. Leight, A. Leisos, A. G. Leister, M. A. L. Leite, R. Leitner, D. Lellouch, B. Lemmer, K. J. C. Leney, T. Lenz, B. Lenzi, R. Leone, S. Leone, C. Leonidopoulos, S. Leontsinis, C. Leroy, C. G. Lester, M. Levchenko, J. Levêque, D. Levin, L. J. Levinson, M. Levy, A. Lewis, A. M. Leyko, M. Leyton, B. Li, H. Li, H. L. Li, L. Li, L. Li, S. Li, X. Li, Y. Li, Z. Liang, H. Liao, B. Liberti, A. Liblong, P. Lichard, K. Lie, J. Liebal, W. Liebig, C. Limbach, A. Limosani, S. C. Lin, T. H. Lin, F. Linde, B. E. Lindquist, J. T. Linnemann, E. Lipeles, A. Lipniacka, M. Lisovyi, T. M. Liss, D. Lissauer, A. Lister, A. M. Litke, B. Liu, D. Liu, H. Liu, J. Liu, J. B. Liu, K. Liu, L. Liu, M. Liu, M. Liu, Y. Liu, M. Livan, A. Lleres, J. Llorente Merino, S. L. Lloyd, F. Lo Sterzo, E. Lobodzinska, P. Loch, W. S. Lockman, F. K. Loebinger, A. E. Loevschall-Jensen, K. M. Loew, A. Loginov, T. Lohse, K. Lohwasser, M. Lokajicek, B. A. Long, J. D. Long, R. E. Long, K. A. Looper, L. Lopes, D. Lopez Mateos, B. Lopez Paredes, I. Lopez Paz, J. Lorenz, N. Lorenzo Martinez, M. Losada, P. J. Lösel, X. Lou, A. Lounis, J. Love, P. A. Love, H. Lu, N. Lu, H. J. Lubatti, C. Luci, A. Lucotte, C. Luedtke, F. Luehring, W. Lukas, L. Luminari, O. Lundberg, B. Lund-Jensen, D. Lynn, R. Lysak, E. Lytken, H. Ma, L. L. Ma, G. Maccarrone, A. Macchiolo, C. M. Macdonald, B. Maček, J. Machado Miguens, D. Macina, D. Madaffari, R. Madar, H. J. Maddocks, W. F. Mader, A. Madsen, J. Maeda, S. Maeland, T. Maeno, A. Maevskiy, E. Magradze, K. Mahboubi, J. Mahlstedt, C. Maiani, C. Maidantchik, A. A. Maier, T. Maier, A. Maio, S. Majewski, Y. Makida, N. Makovec, B. Malaescu, Pa. Malecki, V. P. Maleev, F. Malek, U. Mallik, D. Malon, C. Malone, S. Maltezos, V. M. Malyshev, S. Malyukov, J. Mamuzic, G. Mancini, B. Mandelli, L. Mandelli, I. Mandić, R. Mandrysch, J. Maneira, L. Manhaes de Andrade Filho, J. Manjarres Ramos, A. Mann, A. Manousakis-Katsikakis, B. Mansoulie, R. Mantifel, M. Mantoani, L. Mapelli, L. March, G. Marchiori, M. Marcisovsky, C. P. Marino, M. Marjanovic, D. E. Marley, F. Marroquim, S. P. Marsden, Z. Marshall, L. F. Marti, S. Marti-Garcia, B. Martin, T. A. Martin, V. J. Martin, B. Martin dit Latour, M. Martinez, S. Martin-Haugh, V. S. Martoiu, A. C. Martyniuk, M. Marx, F. Marzano, A. Marzin, L. Masetti, T. Mashimo, R. Mashinistov, J. Masik, A. L. Maslennikov, I. Massa, L. Massa, P. Mastrandrea, A. Mastroberardino, T. Masubuchi, P. Mättig, J. Mattmann, J. Maurer, S. J. Maxfield, D. A. Maximov, R. Mazini, S. M. Mazza, G. Mc Goldrick, S. P. Mc Kee, A. McCarn, R. L. McCarthy, T. G. McCarthy, N. A. McCubbin, K. W. McFarlane, J. A. Mcfayden, G. Mchedlidze, S. J. McMahon, R. A. McPherson, M. Medinnis, S. Meehan, S. Mehlhase, A. Mehta, K. Meier, C. Meineck, B. Meirose, B. R. Mellado Garcia, F. Meloni, A. Mengarelli, S. Menke, E. Meoni, K. M. Mercurio, S. Mergelmeyer, P. Mermod, L. Merola, C. Meroni, F. S. Merritt, A. Messina, J. Metcalfe, A. S. Mete, C. Meyer, C. Meyer, J.-P. Meyer, J. Meyer, H. Meyer Zu Theenhausen, R. P. Middleton, S. Miglioranzi, L. Mijović, G. Mikenberg, M. Mikestikova, M. Mikuž, M. Milesi, A. Milic, D. W. Miller, C. Mills, A. Milov, D. A. Milstead, A. A. Minaenko, Y. Minami, I. A. Minashvili, A. I. Mincer, B. Mindur, M. Mineev, Y. Ming, L. M. Mir, K. P. Mistry, T. Mitani, J. Mitrevski, V. A. Mitsou, A. Miucci, P. S. Miyagawa, J. U. Mjörnmark, T. Moa, K. Mochizuki, S. Mohapatra, W. Mohr, S. Molander, R. Moles-Valls, R. Monden, M. C. Mondragon, K. Mönig, C. Monini, J. Monk, E. Monnier, A. Montalbano, J. Montejo Berlingen, F. Monticelli, S. Monzani, R. W. Moore, N. Morange, D. Moreno, M. Moreno Llácer, P. Morettini, D. Mori, T. Mori, M. Morii, M. Morinaga, V. Morisbak, S. Moritz, A. K. Morley, G. Mornacchi, J. D. Morris, S. S. Mortensen, A. Morton, L. Morvaj, M. Mosidze, J. Moss, K. Motohashi, R. Mount, E. Mountricha, S. V. Mouraviev, E. J. W. Moyse, S. Muanza, R. D. Mudd, F. Mueller, J. Mueller, R. S. P. Mueller, T. Mueller, D. Muenstermann, P. Mullen, G. A. Mullier, F. J. Munoz Sanchez, J. A. Murillo Quijada, W. J. Murray, H. Musheghyan, E. Musto, A. G. Myagkov, M. Myska, B. P. Nachman, O. Nackenhorst, J. Nadal, K. Nagai, R. Nagai, Y. Nagai, K. Nagano, A. Nagarkar, Y. Nagasaka, K. Nagata, M. Nagel, E. Nagy, A. M. Nairz, Y. Nakahama, K. Nakamura, T. Nakamura, I. Nakano, H. Namasivayam, R. F. Naranjo Garcia, R. Narayan, D. I. Narrias Villar, T. Naumann, G. Navarro, R. Nayyar, H. A. Neal, P. Yu. Nechaeva, T. J. Neep, P. D. Nef, A. Negri, M. Negrini, S. Nektarijevic, C. Nellist, A. Nelson, S. Nemecek, P. Nemethy, A. A. Nepomuceno, M. Nessi, M. S. Neubauer, M. Neumann, R. M. Neves, P. Nevski, P. R. Newman, D. H. Nguyen, R. B. Nickerson, R. Nicolaidou, B. Nicquevert, J. Nielsen, N. Nikiforou, A. Nikiforov, V. Nikolaenko, I. Nikolic-Audit, K. Nikolopoulos, J. K. Nilsen, P. Nilsson, Y. Ninomiya, A. Nisati, R. Nisius, T. Nobe, L. Nodulman, M. Nomachi, I. Nomidis, T. Nooney, S. Norberg, M. Nordberg, O. Novgorodova, S. Nowak, M. Nozaki, L. Nozka, K. Ntekas, G. Nunes Hanninger, T. Nunnemann, E. Nurse, F. Nuti, F. O’grady, D. C. O’Neil, V. O’Shea, F. G. Oakham, H. Oberlack, T. Obermann, J. Ocariz, A. Ochi, I. Ochoa, J. P. Ochoa-Ricoux, S. Oda, S. Odaka, H. Ogren, A. Oh, S. H. Oh, C. C. Ohm, H. Ohman, H. Oide, W. Okamura, H. Okawa, Y. Okumura, T. Okuyama, A. Olariu, S. A. Olivares Pino, D. Oliveira Damazio, A. Olszewski, J. Olszowska, A. Onofre, K. Onogi, P. U. E. Onyisi, C. J. Oram, M. J. Oreglia, Y. Oren, D. Orestano, N. Orlando, C. Oropeza Barrera, R. S. Orr, B. Osculati, R. Ospanov, G. Otero y Garzon, H. Otono, M. Ouchrif, F. Ould-Saada, A. Ouraou, K. P. Oussoren, Q. Ouyang, A. Ovcharova, M. Owen, R. E. Owen, V. E. Ozcan, N. Ozturk, K. Pachal, A. Pacheco Pages, C. Padilla Aranda, M. Pagáčová, S. Pagan Griso, E. Paganis, F. Paige, P. Pais, K. Pajchel, G. Palacino, S. Palestini, M. Palka, D. Pallin, A. Palma, Y. B. Pan, E. St. Panagiotopoulou, C. E. Pandini, J. G. Panduro Vazquez, P. Pani, S. Panitkin, D. Pantea, L. Paolozzi, Th. D. Papadopoulou, K. Papageorgiou, A. Paramonov, D. Paredes Hernandez, M. A. Parker, K. A. Parker, F. Parodi, J. A. Parsons, U. Parzefall, E. Pasqualucci, S. Passaggio, F. Pastore, Fr. Pastore, G. Pásztor, S. Pataraia, N. D. Patel, J. R. Pater, T. Pauly, J. Pearce, B. Pearson, L. E. Pedersen, M. Pedersen, S. Pedraza Lopez, R. Pedro, S. V. Peleganchuk, D. Pelikan, O. Penc, C. Peng, H. Peng, B. Penning, J. Penwell, D. V. Perepelitsa, E. Perez Codina, M. T. Pérez García-Estañ, L. Perini, H. Pernegger, S. Perrella, R. Peschke, V. D. Peshekhonov, K. Peters, R. F. Y. Peters, B. A. Petersen, T. C. Petersen, E. Petit, A. Petridis, C. Petridou, P. Petroff, E. Petrolo, F. Petrucci, N. E. Pettersson, R. Pezoa, P. W. Phillips, G. Piacquadio, E. Pianori, A. Picazio, E. Piccaro, M. Piccinini, M. A. Pickering, R. Piegaia, D. T. Pignotti, J. E. Pilcher, A. D. Pilkington, A. W. J. Pin, J. Pina, M. Pinamonti, J. L. Pinfold, A. Pingel, S. Pires, H. Pirumov, M. Pitt, C. Pizio, L. Plazak, M.-A. Pleier, V. Pleskot, E. Plotnikova, P. Plucinski, D. Pluth, R. Poettgen, L. Poggioli, D. Pohl, G. Polesello, A. Poley, A. Policicchio, R. Polifka, A. Polini, C. S. Pollard, V. Polychronakos, K. Pommès, L. Pontecorvo, B. G. Pope, G. A. Popeneciu, D. S. Popovic, A. Poppleton, S. Pospisil, K. Potamianos, I. N. Potrap, C. J. Potter, C. T. Potter, G. Poulard, J. Poveda, V. Pozdnyakov, M. E. Pozo Astigarraga, P. Pralavorio, A. Pranko, S. Prasad, S. Prell, D. Price, L. E. Price, M. Primavera, S. Prince, M. Proissl, K. Prokofiev, F. Prokoshin, E. Protopapadaki, S. Protopopescu, J. Proudfoot, M. Przybycien, E. Ptacek, D. Puddu, E. Pueschel, D. Puldon, M. Purohit, P. Puzo, J. Qian, G. Qin, Y. Qin, A. Quadt, D. R. Quarrie, W. B. Quayle, M. Queitsch-Maitland, D. Quilty, S. Raddum, V. Radeka, V. Radescu, S. K. Radhakrishnan, P. Radloff, P. Rados, F. Ragusa, G. Rahal, S. Rajagopalan, M. Rammensee, C. Rangel-Smith, F. Rauscher, S. Rave, T. Ravenscroft, M. Raymond, A. L. Read, N. P. Readioff, D. M. Rebuzzi, A. Redelbach, G. Redlinger, R. Reece, K. Reeves, L. Rehnisch, J. Reichert, H. Reisin, C. Rembser, H. Ren, A. Renaud, M. Rescigno, S. Resconi, O. L. Rezanova, P. Reznicek, R. Rezvani, R. Richter, S. Richter, E. Richter-Was, O. Ricken, M. Ridel, P. Rieck, C. J. Riegel, J. Rieger, O. Rifki, M. Rijssenbeek, A. Rimoldi, L. Rinaldi, B. Ristić, E. Ritsch, I. Riu, F. Rizatdinova, E. Rizvi, S. H. Robertson, A. Robichaud-Veronneau, D. Robinson, J. E. M. Robinson, A. Robson, C. Roda, S. Roe, O. Røhne, A. Romaniouk, M. Romano, S. M. Romano Saez, E. Romero Adam, N. Rompotis, M. Ronzani, L. Roos, E. Ros, S. Rosati, K. Rosbach, P. Rose, O. Rosenthal, V. Rossetti, E. Rossi, L. P. Rossi, J. H. N. Rosten, R. Rosten, M. Rotaru, I. Roth, J. Rothberg, D. Rousseau, C. R. Royon, A. Rozanov, Y. Rozen, X. Ruan, F. Rubbo, I. Rubinskiy, V. I. Rud, C. Rudolph, M. S. Rudolph, F. Rühr, A. Ruiz-Martinez, Z. Rurikova, N. A. Rusakovich, A. Ruschke, H. L. Russell, J. P. Rutherfoord, N. Ruthmann, Y. F. Ryabov, M. Rybar, G. Rybkin, N. C. Ryder, A. Ryzhov, A. F. Saavedra, G. Sabato, S. Sacerdoti, A. Saddique, H. F.-W. Sadrozinski, R. Sadykov, F. Safai Tehrani, P. Saha, M. Sahinsoy, M. Saimpert, T. Saito, H. Sakamoto, Y. Sakurai, G. Salamanna, A. Salamon, J. E. Salazar Loyola, M. Saleem, D. Salek, P. H. Sales De Bruin, D. Salihagic, A. Salnikov, J. Salt, D. Salvatore, F. Salvatore, A. Salvucci, A. Salzburger, D. Sammel, D. Sampsonidis, A. Sanchez, J. Sánchez, V. Sanchez Martinez, H. Sandaker, R. L. Sandbach, H. G. Sander, M. P. Sanders, M. Sandhoff, C. Sandoval, R. Sandstroem, D. P. C. Sankey, M. Sannino, A. Sansoni, C. Santoni, R. Santonico, H. Santos, I. Santoyo Castillo, K. Sapp, A. Sapronov, J. G. Saraiva, B. Sarrazin, O. Sasaki, Y. Sasaki, K. Sato, G. Sauvage, E. Sauvan, G. Savage, P. Savard, C. Sawyer, L. Sawyer, J. Saxon, C. Sbarra, A. Sbrizzi, T. Scanlon, D. A. Scannicchio, M. Scarcella, V. Scarfone, J. Schaarschmidt, P. Schacht, D. Schaefer, R. Schaefer, J. Schaeffer, S. Schaepe, S. Schaetzel, U. Schäfer, A. C. Schaffer, D. Schaile, R. D. Schamberger, V. Scharf, V. A. Schegelsky, D. Scheirich, M. Schernau, C. Schiavi, C. Schillo, M. Schioppa, S. Schlenker, K. Schmieden, C. Schmitt, S. Schmitt, S. Schmitt, S. Schmitz, B. Schneider, Y. J. Schnellbach, U. Schnoor, L. Schoeffel, A. Schoening, B. D. Schoenrock, E. Schopf, A. L. S. Schorlemmer, M. Schott, D. Schouten, J. Schovancova, S. Schramm, M. Schreyer, N. Schuh, M. J. Schultens, H.-C. Schultz-Coulon, H. Schulz, M. Schumacher, B. A. Schumm, Ph. Schune, C. Schwanenberger, A. Schwartzman, T. A. Schwarz, Ph. Schwegler, H. Schweiger, Ph. Schwemling, R. Schwienhorst, J. Schwindling, T. Schwindt, E. Scifo, G. Sciolla, F. Scuri, F. Scutti, J. Searcy, G. Sedov, E. Sedykh, P. Seema, S. C. Seidel, A. Seiden, F. Seifert, J. M. Seixas, G. Sekhniaidze, K. Sekhon, S. J. Sekula, D. M. Seliverstov, N. Semprini-Cesari, C. Serfon, L. Serin, L. Serkin, T. Serre, M. Sessa, R. Seuster, H. Severini, T. Sfiligoj, F. Sforza, A. Sfyrla, E. Shabalina, M. Shamim, L. Y. Shan, R. Shang, J. T. Shank, M. Shapiro, P. B. Shatalov, K. Shaw, S. M. Shaw, A. Shcherbakova, C. Y. Shehu, P. Sherwood, L. Shi, S. Shimizu, C. O. Shimmin, M. Shimojima, M. Shiyakova, A. Shmeleva, D. Shoaleh Saadi, M. J. Shochet, S. Shojaii, S. Shrestha, E. Shulga, M. A. Shupe, P. Sicho, P. E. Sidebo, O. Sidiropoulou, D. Sidorov, A. Sidoti, F. Siegert, Dj. Sijacki, J. Silva, Y. Silver, S. B. Silverstein, V. Simak, O. Simard, Lj. Simic, S. Simion, E. Simioni, B. Simmons, D. Simon, M. Simon, P. Sinervo, N. B. Sinev, M. Sioli, G. Siragusa, A. N. Sisakyan, S. Yu. Sivoklokov, J. Sjölin, T. B. Sjursen, M. B. Skinner, H. P. Skottowe, P. Skubic, M. Slater, T. Slavicek, M. Slawinska, K. Sliwa, V. Smakhtin, B. H. Smart, L. Smestad, S. Yu. Smirnov, Y. Smirnov, L. N. Smirnova, O. Smirnova, M. N. K. Smith, R. W. Smith, M. Smizanska, K. Smolek, A. A. Snesarev, G. Snidero, S. Snyder, R. Sobie, F. Socher, A. Soffer, D. A. Soh, G. Sokhrannyi, C. A. Solans Sanchez, M. Solar, J. Solc, E. Yu. Soldatov, U. Soldevila, A. A. Solodkov, A. Soloshenko, O. V. Solovyanov, V. Solovyev, P. Sommer, H. Y. Song, N. Soni, A. Sood, A. Sopczak, B. Sopko, V. Sopko, V. Sorin, D. Sosa, M. Sosebee, C. L. Sotiropoulou, R. Soualah, A. M. Soukharev, D. South, B. C. Sowden, S. Spagnolo, M. Spalla, M. Spangenberg, F. Spanò, W. R. Spearman, D. Sperlich, F. Spettel, R. Spighi, G. Spigo, L. A. Spiller, M. Spousta, R. D. St. Denis, A. Stabile, S. Staerz, J. Stahlman, R. Stamen, S. Stamm, E. Stanecka, R. W. Stanek, C. Stanescu, M. Stanescu-Bellu, M. M. Stanitzki, S. Stapnes, E. A. Starchenko, J. Stark, P. Staroba, P. Starovoitov, R. Staszewski, P. Steinberg, B. Stelzer, H. J. Stelzer, O. Stelzer-Chilton, H. Stenzel, G. A. Stewart, J. A. Stillings, M. C. Stockton, M. Stoebe, G. Stoicea, P. Stolte, S. Stonjek, A. R. Stradling, A. Straessner, M. E. Stramaglia, J. Strandberg, S. Strandberg, A. Strandlie, E. Strauss, M. Strauss, P. Strizenec, R. Ströhmer, D. M. Strom, R. Stroynowski, A. Strubig, S. A. Stucci, B. Stugu, N. A. Styles, D. Su, J. Su, R. Subramaniam, A. Succurro, S. Suchek, Y. Sugaya, M. Suk, V. V. Sulin, S. Sultansoy, T. Sumida, S. Sun, X. Sun, J. E. Sundermann, K. Suruliz, G. Susinno, M. R. Sutton, S. Suzuki, M. Svatos, M. Swiatlowski, I. Sykora, T. Sykora, D. Ta, C. Taccini, K. Tackmann, J. Taenzer, A. Taffard, R. Tafirout, N. Taiblum, H. Takai, R. Takashima, H. Takeda, T. Takeshita, Y. Takubo, M. Talby, A. A. Talyshev, J. Y. C. Tam, K. G. Tan, J. Tanaka, R. Tanaka, S. Tanaka, B. B. Tannenwald, S. Tapia Araya, S. Tapprogge, S. Tarem, F. Tarrade, G. F. Tartarelli, P. Tas, M. Tasevsky, T. Tashiro, E. Tassi, A. Tavares Delgado, Y. Tayalati, A. C. Taylor, F. E. Taylor, G. N. Taylor, P. T. E. Taylor, W. Taylor, F. A. Teischinger, P. Teixeira-Dias, K. K. Temming, D. Temple, H. Ten Kate, P. K. Teng, J. J. Teoh, F. Tepel, S. Terada, K. Terashi, J. Terron, S. Terzo, M. Testa, R. J. Teuscher, T. Theveneaux-Pelzer, J. P. Thomas, J. Thomas-Wilsker, E. N. Thompson, P. D. Thompson, R. J. Thompson, A. S. Thompson, L. A. Thomsen, E. Thomson, M. Thomson, R. P. Thun, M. J. Tibbetts, R. E. Ticse Torres, V. O. Tikhomirov, Yu. A. Tikhonov, S. Timoshenko, E. Tiouchichine, P. Tipton, S. Tisserant, K. Todome, T. Todorov, S. Todorova-Nova, J. Tojo, S. Tokár, K. Tokushuku, K. Tollefson, E. Tolley, L. Tomlinson, M. Tomoto, L. Tompkins, K. Toms, E. Torrence, H. Torres, E. Torró Pastor, J. Toth, F. Touchard, D. R. Tovey, T. Trefzger, L. Tremblet, A. Tricoli, I. M. Trigger, S. Trincaz-Duvoid, M. F. Tripiana, W. Trischuk, B. Trocmé, C. Troncon, M. Trottier-McDonald, M. Trovatelli, L. Truong, M. Trzebinski, A. Trzupek, C. Tsarouchas, J. C.-L. Tseng, P. V. Tsiareshka, D. Tsionou, G. Tsipolitis, N. Tsirintanis, S. Tsiskaridze, V. Tsiskaridze, E. G. Tskhadadze, K. M. Tsui, I. I. Tsukerman, V. Tsulaia, S. Tsuno, D. Tsybychev, A. Tudorache, V. Tudorache, A. N. Tuna, S. A. Tupputi, S. Turchikhin, D. Turecek, R. Turra, A. J. Turvey, P. M. Tuts, A. Tykhonov, M. Tylmad, M. Tyndel, I. Ueda, R. Ueno, M. Ughetto, F. Ukegawa, G. Unal, A. Undrus, G. Unel, F. C. Ungaro, Y. Unno, C. Unverdorben, J. Urban, P. Urquijo, P. Urrejola, G. Usai, A. Usanova, L. Vacavant, V. Vacek, B. Vachon, C. Valderanis, N. Valencic, S. Valentinetti, A. Valero, L. Valery, S. Valkar, S. Vallecorsa, J. A. Valls Ferrer, W. Van Den Wollenberg, P. C. Van Der Deijl, R. van der Geer, H. van der Graaf, N. van Eldik, P. van Gemmeren, J. Van Nieuwkoop, I. van Vulpen, M. C. van Woerden, M. Vanadia, W. Vandelli, R. Vanguri, A. Vaniachine, F. Vannucci, G. Vardanyan, R. Vari, E. W. Varnes, T. Varol, D. Varouchas, A. Vartapetian, K. E. Varvell, F. Vazeille, T. Vazquez Schroeder, J. Veatch, L. M. Veloce, F. Veloso, T. Velz, S. Veneziano, A. Ventura, D. Ventura, M. Venturi, N. Venturi, A. Venturini, V. Vercesi, M. Verducci, W. Verkerke, J. C. Vermeulen, A. Vest, M. C. Vetterli, O. Viazlo, I. Vichou, T. Vickey, O. E. Vickey Boeriu, G. H. A. Viehhauser, S. Viel, R. Vigne, M. Villa, M. Villaplana Perez, E. Vilucchi, M. G. Vincter, V. B. Vinogradov, I. Vivarelli, S. Vlachos, D. Vladoiu, M. Vlasak, M. Vogel, P. Vokac, G. Volpi, M. Volpi, H. von der Schmitt, H. von Radziewski, E. von Toerne, V. Vorobel, K. Vorobev, M. Vos, R. Voss, J. H. Vossebeld, N. Vranjes, M. Vranjes Milosavljevic, V. Vrba, M. Vreeswijk, R. Vuillermet, I. Vukotic, Z. Vykydal, P. Wagner, W. Wagner, H. Wahlberg, S. Wahrmund, J. Wakabayashi, J. Walder, R. Walker, W. Walkowiak, C. Wang, F. Wang, H. Wang, H. Wang, J. Wang, J. Wang, K. Wang, R. Wang, S. M. Wang, T. Wang, T. Wang, X. Wang, C. Wanotayaroj, A. Warburton, C. P. Ward, D. R. Wardrope, A. Washbrook, C. Wasicki, P. M. Watkins, A. T. Watson, I. J. Watson, M. F. Watson, G. Watts, S. Watts, B. M. Waugh, S. Webb, M. S. Weber, S. W. Weber, J. S. Webster, A. R. Weidberg, B. Weinert, J. Weingarten, C. Weiser, H. Weits, P. S. Wells, T. Wenaus, T. Wengler, S. Wenig, N. Wermes, M. Werner, P. Werner, M. Wessels, J. Wetter, K. Whalen, A. M. Wharton, A. White, M. J. White, R. White, S. White, D. Whiteson, F. J. Wickens, W. Wiedenmann, M. Wielers, P. Wienemann, C. Wiglesworth, L. A. M. Wiik-Fuchs, A. Wildauer, H. G. Wilkens, H. H. Williams, S. Williams, C. Willis, S. Willocq, A. Wilson, J. A. Wilson, I. Wingerter-Seez, F. Winklmeier, B. T. Winter, M. Wittgen, J. Wittkowski, S. J. Wollstadt, M. W. Wolter, H. Wolters, B. K. Wosiek, J. Wotschack, M. J. Woudstra, K. W. Wozniak, M. Wu, M. Wu, S. L. Wu, X. Wu, Y. Wu, T. R. Wyatt, B. M. Wynne, S. Xella, D. Xu, L. Xu, B. Yabsley, S. Yacoob, R. Yakabe, M. Yamada, D. Yamaguchi, Y. Yamaguchi, A. Yamamoto, S. Yamamoto, T. Yamanaka, K. Yamauchi, Y. Yamazaki, Z. Yan, H. Yang, H. Yang, Y. Yang, W.-M. Yao, Y. C. Yap, Y. Yasu, E. Yatsenko, K. H. Yau Wong, J. Ye, S. Ye, I. Yeletskikh, A. L. Yen, E. Yildirim, K. Yorita, R. Yoshida, K. Yoshihara, C. Young, C. J. S. Young, S. Youssef, D. R. Yu, J. Yu, J. M. Yu, J. Yu, L. Yuan, S. P. Y. Yuen, A. Yurkewicz, I. Yusuff, B. Zabinski, R. Zaidan, A. M. Zaitsev, J. Zalieckas, A. Zaman, S. Zambito, L. Zanello, D. Zanzi, C. Zeitnitz, M. Zeman, A. Zemla, J. C. Zeng, Q. Zeng, K. Zengel, O. Zenin, T. Ženiš, D. Zerwas, D. Zhang, F. Zhang, G. Zhang, H. Zhang, J. Zhang, L. Zhang, R. Zhang, X. Zhang, Z. Zhang, X. Zhao, Y. Zhao, Z. Zhao, A. Zhemchugov, J. Zhong, B. Zhou, C. Zhou, L. Zhou, L. Zhou, M. Zhou, N. Zhou, C. G. Zhu, H. Zhu, J. Zhu, Y. Zhu, X. Zhuang, K. Zhukov, A. Zibell, D. Zieminska, N. I. Zimine, C. Zimmermann, S. Zimmermann, Z. Zinonos, M. Zinser, M. Ziolkowski, L. Živković, G. Zobernig, A. Zoccoli, M. zur Nedden, G. Zurzolo, L. Zwalinski, ATLAS Collaboration

**Affiliations:** 1 Department of Physics, University of Adelaide, Adelaide, Australia; 2 Physics Department, SUNY Albany, Albany, NY USA; 3 Department of Physics, University of Alberta, Edmonton, AB Canada; 4 Department of Physics, Ankara University, Ankara, Turkey; 5 Istanbul Aydin University, Istanbul, Turkey; 6 Division of Physics, TOBB University of Economics and Technology, Ankara, Turkey; 7 LAPP, CNRS/IN2P3 and Université Savoie Mont Blanc, Annecy-le-Vieux, France; 8 High Energy Physics Division, Argonne National Laboratory, Argonne, IL USA; 9 Department of Physics, University of Arizona, Tucson, AZ USA; 10 Department of Physics, The University of Texas at Arlington, Arlington, TX USA; 11 Physics Department, University of Athens, Athens, Greece; 12 Physics Department, National Technical University of Athens, Zografou, Greece; 13 Institute of Physics, Azerbaijan Academy of Sciences, Baku, Azerbaijan; 14 Institut de Física d’Altes Energies (IFAE), The Barcelona Institute of Science and Technology, Barcelona, Spain; 15 Institute of Physics, University of Belgrade, Belgrade, Serbia; 16 Department for Physics and Technology, University of Bergen, Bergen, Norway; 17 Physics Division, Lawrence Berkeley National Laboratory and University of California, Berkeley, CA USA; 18 Department of Physics, Humboldt University, Berlin, Germany; 19 Albert Einstein Center for Fundamental Physics and Laboratory for High Energy Physics, University of Bern, Bern, Switzerland; 20 School of Physics and Astronomy, University of Birmingham, Birmingham, UK; 21 Department of Physics, Bogazici University, Istanbul, Turkey; 22 Department of Physics Engineering, Gaziantep University, Gaziantep, Turkey; 23 Department of Physics, Dogus University, Istanbul, Turkey; 24 Centro de Investigaciones, Universidad Antonio Narino, Bogotá, Colombia; 25 INFN Sezione di Bologna, Bologna, Italy; 26 Dipartimento di Fisica e Astronomia, Università di Bologna, Bologna, Italy; 27 Physikalisches Institut, University of Bonn, Bonn, Germany; 28 Department of Physics, Boston University, Boston, MA USA; 29 Department of Physics, Brandeis University, Waltham, MA USA; 30 Universidade Federal do Rio De Janeiro COPPE/EE/IF, Rio de Janeiro, Brazil; 31 Electrical Circuits Department, Federal University of Juiz de Fora (UFJF), Juiz de Fora, Brazil; 32 Federal University of Sao Joao del Rei (UFSJ), Sao Joao del Rei, Brazil; 33 Instituto de Fisica, Universidade de Sao Paulo, Sao Paulo, Brazil; 34 Physics Department, Brookhaven National Laboratory, Upton, NY USA; 35 Transilvania University of Brasov, Brasov, Romania; 36 National Institute of Physics and Nuclear Engineering, Bucharest, Romania; 37 Physics Department, National Institute for Research and Development of Isotopic and Molecular Technologies, Cluj Napoca, Romania; 38 University Politehnica Bucharest, Bucharest, Romania; 39 West University in Timisoara, Timisoara, Romania; 40 Departamento de Física, Universidad de Buenos Aires, Buenos Aires, Argentina; 41 Cavendish Laboratory, University of Cambridge, Cambridge, UK; 42 Department of Physics, Carleton University, Ottawa, ON Canada; 43 CERN, Geneva, Switzerland; 44 Enrico Fermi Institute, University of Chicago, Chicago, IL USA; 45 Departamento de Física, Pontificia Universidad Católica de Chile, Santiago, Chile; 46 Departamento de Física, Universidad Técnica Federico Santa María, Valparaiso, Chile; 47 Institute of High Energy Physics, Chinese Academy of Sciences, Beijing, China; 48 Department of Modern Physics, University of Science and Technology of China, Hefei, Anhui China; 49 Department of Physics, Nanjing University, Nanjing, Jiangsu China; 50 School of Physics, Shandong University, Jinan, Shandong China; 51 Department of Physics and Astronomy, Shanghai Key Laboratory for Particle Physics and Cosmology (also affiliated with PKU-CHEP), Shanghai Jiao Tong University, Shanghai, China; 52 Physics Department, Tsinghua University, Beijing, 100084 China; 53 Laboratoire de Physique Corpusculaire, Clermont Université and Université Blaise Pascal and CNRS/IN2P3, Clermont-Ferrand, France; 54 Nevis Laboratory, Columbia University, Irvington, NY USA; 55 Niels Bohr Institute, University of Copenhagen, Copenhagen, Denmark; 56 INFN Gruppo Collegato di Cosenza, Laboratori Nazionali di Frascati, Frascati, Italy; 57 Dipartimento di Fisica, Università della Calabria, Rende, Italy; 58 Faculty of Physics and Applied Computer Science, AGH University of Science and Technology, Kraków, Poland; 59 Marian Smoluchowski Institute of Physics, Jagiellonian University, Kraków, Poland; 60 Institute of Nuclear Physics, Polish Academy of Sciences, Kraków, Poland; 61 Physics Department, Southern Methodist University, Dallas, TX USA; 62 Physics Department, University of Texas at Dallas, Richardson, TX USA; 63 DESY, Hamburg and Zeuthen, Germany; 64 Institut für Experimentelle Physik IV, Technische Universität Dortmund, Dortmund, Germany; 65 Institut für Kern- und Teilchenphysik, Technische Universität Dresden, Dresden, Germany; 66 Department of Physics, Duke University, Durham, NC USA; 67 SUPA-School of Physics and Astronomy, University of Edinburgh, Edinburgh, UK; 68 INFN Laboratori Nazionali di Frascati, Frascati, Italy; 69 Fakultät für Mathematik und Physik, Albert-Ludwigs-Universität, Freiburg, Germany; 70 Section de Physique, Université de Genève, Geneva, Switzerland; 71 INFN Sezione di Genova, Genoa, Italy; 72 Dipartimento di Fisica, Università di Genova, Genoa, Italy; 73 E. Andronikashvili Institute of Physics, Iv. Javakhishvili Tbilisi State University, Tbilisi, Georgia; 74 High Energy Physics Institute, Tbilisi State University, Tbilisi, Georgia; 75 II Physikalisches Institut, Justus-Liebig-Universität Giessen, Giessen, Germany; 76 SUPA-School of Physics and Astronomy, University of Glasgow, Glasgow, UK; 77 II Physikalisches Institut, Georg-August-Universität, Göttingen, Germany; 78 Laboratoire de Physique Subatomique et de Cosmologie, Université Grenoble-Alpes, CNRS/IN2P3, Grenoble, France; 79 Department of Physics, Hampton University, Hampton, VA USA; 80 Laboratory for Particle Physics and Cosmology, Harvard University, Cambridge, MA USA; 81 Kirchhoff-Institut für Physik, Ruprecht-Karls-Universität Heidelberg, Heidelberg, Germany; 82 Physikalisches Institut, Ruprecht-Karls-Universität Heidelberg, Heidelberg, Germany; 83 ZITI Institut für technische Informatik, Ruprecht-Karls-Universität Heidelberg, Mannheim, Germany; 84 Faculty of Applied Information Science, Hiroshima Institute of Technology, Hiroshima, Japan; 85 Department of Physics, The Chinese University of Hong Kong, Shatin, N.T. Hong Kong; 86 Department of Physics, The University of Hong Kong, Hong Kong, China; 87 Department of Physics, The Hong Kong University of Science and Technology, Clear Water Bay, Kowloon, Hong Kong, China; 88 Department of Physics, Indiana University, Bloomington, IN USA; 89 Institut für Astro- und Teilchenphysik, Leopold-Franzens-Universität, Innsbruck, Austria; 90 University of Iowa, Iowa City, IA USA; 91 Department of Physics and Astronomy, Iowa State University, Ames, IA USA; 92 Joint Institute for Nuclear Research, JINR Dubna, Dubna, Russia; 93 KEK, High Energy Accelerator Research Organization, Tsukuba, Japan; 94 Graduate School of Science, Kobe University, Kobe, Japan; 95 Faculty of Science, Kyoto University, Kyoto, Japan; 96 Kyoto University of Education, Kyoto, Japan; 97 Department of Physics, Kyushu University, Fukuoka, Japan; 98 Instituto de Física La Plata, Universidad Nacional de La Plata and CONICET, La Plata, Argentina; 99 Physics Department, Lancaster University, Lancaster, UK; 100 INFN Sezione di Lecce, Lecce, Italy; 101 Dipartimento di Matematica e Fisica, Università del Salento, Lecce, Italy; 102 Oliver Lodge Laboratory, University of Liverpool, Liverpool, UK; 103 Department of Physics, Jožef Stefan Institute and University of Ljubljana, Ljubljana, Slovenia; 104 School of Physics and Astronomy, Queen Mary University of London, London, UK; 105 Department of Physics, Royal Holloway University of London, Surrey, UK; 106 Department of Physics and Astronomy, University College London, London, UK; 107 Louisiana Tech University, Ruston, LA USA; 108 Laboratoire de Physique Nucléaire et de Hautes Energies, UPMC and Université Paris-Diderot and CNRS/IN2P3, Paris, France; 109 Fysiska institutionen, Lunds universitet, Lund, Sweden; 110 Departamento de Fisica Teorica C-15, Universidad Autonoma de Madrid, Madrid, Spain; 111 Institut für Physik, Universität Mainz, Mainz, Germany; 112 School of Physics and Astronomy, University of Manchester, Manchester, UK; 113 CPPM, Aix-Marseille Université and CNRS/IN2P3, Marseille, France; 114 Department of Physics, University of Massachusetts, Amherst, MA USA; 115 Department of Physics, McGill University, Montreal, QC Canada; 116 School of Physics, University of Melbourne, Melbourne, VIC Australia; 117 Department of Physics, The University of Michigan, Ann Arbor, MI USA; 118 Department of Physics and Astronomy, Michigan State University, East Lansing, MI USA; 119 INFN Sezione di Milano, Milan, Italy; 120 Dipartimento di Fisica, Università di Milano, Milan, Italy; 121 B.I. Stepanov Institute of Physics, National Academy of Sciences of Belarus, Minsk, Republic of Belarus; 122 National Scientific and Educational Centre for Particle and High Energy Physics, Minsk, Republic of Belarus; 123 Department of Physics, Massachusetts Institute of Technology, Cambridge, MA USA; 124 Group of Particle Physics, University of Montreal, Montreal, QC Canada; 125 P.N. Lebedev Physical Institute of the Russian Academy of Sciences, Moscow, Russia; 126 Institute for Theoretical and Experimental Physics (ITEP), Moscow, Russia; 127 National Research Nuclear University MEPhI, Moscow, Russia; 128 D.V. Skobeltsyn Institute of Nuclear Physics, M.V. Lomonosov Moscow State University, Moscow, Russia; 129 Fakultät für Physik, Ludwig-Maximilians-Universität München, Munich, Germany; 130 Max-Planck-Institut für Physik (Werner-Heisenberg-Institut), Munich, Germany; 131 Nagasaki Institute of Applied Science, Nagasaki, Japan; 132 Graduate School of Science and Kobayashi-Maskawa Institute, Nagoya University, Nagoya, Japan; 133 INFN Sezione di Napoli, Naples, Italy; 134 Dipartimento di Fisica, Università di Napoli, Naples, Italy; 135 Department of Physics and Astronomy, University of New Mexico, Albuquerque, NM USA; 136 Institute for Mathematics, Astrophysics and Particle Physics, Radboud University Nijmegen/Nikhef, Nijmegen, The Netherlands; 137 Nikhef National Institute for Subatomic Physics and University of Amsterdam, Amsterdam, The Netherlands; 138 Department of Physics, Northern Illinois University, DeKalb, IL USA; 139 Budker Institute of Nuclear Physics, SB RAS, Novosibirsk, Russia; 140 Department of Physics, New York University, New York, NY USA; 141 Ohio State University, Columbus, OH USA; 142 Faculty of Science, Okayama University, Okayama, Japan; 143 Homer L. Dodge Department of Physics and Astronomy, University of Oklahoma, Norman, OK USA; 144 Department of Physics, Oklahoma State University, Stillwater, OK USA; 145 Palacký University, RCPTM, Olomouc, Czech Republic; 146 Center for High Energy Physics, University of Oregon, Eugene, OR USA; 147 LAL, Univ. Paris-Sud, CNRS/IN2P3, Université Paris-Saclay, Orsay, France; 148 Graduate School of Science, Osaka University, Osaka, Japan; 149 Department of Physics, University of Oslo, Oslo, Norway; 150 Department of Physics, Oxford University, Oxford, UK; 151 INFN Sezione di Pavia, Pavia, Italy; 152 Dipartimento di Fisica, Università di Pavia, Pavia, Italy; 153 Department of Physics, University of Pennsylvania, Philadelphia, PA USA; 154 National Research Centre “Kurchatov Institute” B.P. Konstantinov Petersburg Nuclear Physics Institute, St. Petersburg, Russia; 155 INFN Sezione di Pisa, Pisa, Italy; 156 Dipartimento di Fisica E. Fermi, Università di Pisa, Pisa, Italy; 157 Department of Physics and Astronomy, University of Pittsburgh, Pittsburgh, PA USA; 158 Laboratório de Instrumentação e Física Experimental de Partículas-LIP, Lisbon, Portugal; 159 Faculdade de Ciências, Universidade de Lisboa, Lisbon, Portugal; 160 Department of Physics, University of Coimbra, Coimbra, Portugal; 161 Centro de Física Nuclear da Universidade de Lisboa, Lisbon, Portugal; 162 Departamento de Fisica, Universidade do Minho, Braga, Portugal; 163 Departamento de Fisica Teorica y del Cosmos and CAFPE, Universidad de Granada, Granada, Spain; 164 Dep Fisica and CEFITEC of Faculdade de Ciencias e Tecnologia, Universidade Nova de Lisboa, Caparica, Portugal; 165 Institute of Physics, Academy of Sciences of the Czech Republic, Prague, Czech Republic; 166 Czech Technical University in Prague, Prague, Czech Republic; 167 Faculty of Mathematics and Physics, Charles University in Prague, Prague, Czech Republic; 168 State Research Center Institute for High Energy Physics (Protvino), NRC KI, Moscow, Russia; 169 Particle Physics Department, Rutherford Appleton Laboratory, Didcot, UK; 170 INFN Sezione di Roma, Rome, Italy; 171 Dipartimento di Fisica, Sapienza Università di Roma, Rome, Italy; 172 INFN Sezione di Roma Tor Vergata, Rome, Italy; 173 Dipartimento di Fisica, Università di Roma Tor Vergata, Rome, Italy; 174 INFN Sezione di Roma Tre, Rome, Italy; 175 Dipartimento di Matematica e Fisica, Università Roma Tre, Rome, Italy; 176 Faculté des Sciences Ain Chock, Réseau Universitaire de Physique des Hautes Energies-Université Hassan II, Casablanca, Morocco; 177 Centre National de l’Energie des Sciences Techniques Nucleaires, Rabat, Morocco; 178 Faculté des Sciences Semlalia, Université Cadi Ayyad, LPHEA-Marrakech, Marrakech, Morocco; 179 Faculté des Sciences, Université Mohamed Premier and LPTPM, Oujda, Morocco; 180 Faculté des Sciences, Université Mohammed V, Rabat, Morocco; 181 DSM/IRFU (Institut de Recherches sur les Lois Fondamentales de l’Univers), CEA Saclay (Commissariat à l’Energie Atomique et aux Energies Alternatives), Gif-sur-Yvette, France; 182 Santa Cruz Institute for Particle Physics, University of California Santa Cruz, Santa Cruz, CA USA; 183 Department of Physics, University of Washington, Seattle, WA USA; 184 Department of Physics and Astronomy, University of Sheffield, Sheffield, UK; 185 Department of Physics, Shinshu University, Nagano, Japan; 186 Fachbereich Physik, Universität Siegen, Siegen, Germany; 187 Department of Physics, Simon Fraser University, Burnaby, BC Canada; 188 SLAC National Accelerator Laboratory, Stanford, CA USA; 189 Faculty of Mathematics, Physics and Informatics, Comenius University, Bratislava, Slovak Republic; 190 Department of Subnuclear Physics, Institute of Experimental Physics of the Slovak Academy of Sciences, Kosice, Slovak Republic; 191 Department of Physics, University of Cape Town, Cape Town, South Africa; 192 Department of Physics, University of Johannesburg, Johannesburg, South Africa; 193 School of Physics, University of the Witwatersrand, Johannesburg, South Africa; 194 Department of Physics, Stockholm University, Stockholm, Sweden; 195 The Oskar Klein Centre, Stockholm, Sweden; 196 Physics Department, Royal Institute of Technology, Stockholm, Sweden; 197 Departments of Physics and Astronomy and Chemistry, Stony Brook University, Stony Brook, NY USA; 198 Department of Physics and Astronomy, University of Sussex, Brighton, UK; 199 School of Physics, University of Sydney, Sydney, Australia; 200 Institute of Physics, Academia Sinica, Taipei, Taiwan; 201 Department of Physics, Technion: Israel Institute of Technology, Haifa, Israel; 202 Raymond and Beverly Sackler School of Physics and Astronomy, Tel Aviv University, Tel Aviv, Israel; 203 Department of Physics, Aristotle University of Thessaloniki, Thessaloníki, Greece; 204 International Center for Elementary Particle Physics and Department of Physics, The University of Tokyo, Tokyo, Japan; 205 Graduate School of Science and Technology, Tokyo Metropolitan University, Tokyo, Japan; 206 Department of Physics, Tokyo Institute of Technology, Tokyo, Japan; 207 Department of Physics, University of Toronto, Toronto, ON Canada; 208 TRIUMF, Vancouver, BC Canada; 209 Department of Physics and Astronomy, York University, Toronto, ON Canada; 210 Faculty of Pure and Applied Sciences, and Center for Integrated Research in Fundamental Science and Engineering, University of Tsukuba, Tsukuba, Japan; 211 Department of Physics and Astronomy, Tufts University, Medford, MA USA; 212 Department of Physics and Astronomy, University of California Irvine, Irvine, CA USA; 213 INFN Gruppo Collegato di Udine, Sezione di Trieste, Udine, Italy; 214 ICTP, Trieste, Italy; 215 Dipartimento di Chimica, Fisica e Ambiente, Università di Udine, Udine, Italy; 216 Department of Physics and Astronomy, University of Uppsala, Uppsala, Sweden; 217 Department of Physics, University of Illinois, Urbana, IL USA; 218 Instituto de Física Corpuscular (IFIC) and Departamento de Física Atómica, Molecular y Nuclear and Departamento de Ingeniería Electrónica and Instituto de Microelectrónica de Barcelona (IMB-CNM), University of Valencia and CSIC, Valencia, Spain; 219 Department of Physics, University of British Columbia, Vancouver, BC Canada; 220 Department of Physics and Astronomy, University of Victoria, Victoria, BC Canada; 221 Department of Physics, University of Warwick, Coventry, UK; 222 Waseda University, Tokyo, Japan; 223 Department of Particle Physics, The Weizmann Institute of Science, Rehovot, Israel; 224 Department of Physics, University of Wisconsin, Madison, WI USA; 225 Fakultät für Physik und Astronomie, Julius-Maximilians-Universität, Würzburg, Germany; 226 Fakultät für Mathematik und Naturwissenschaften, Fachgruppe Physik, Bergische Universität Wuppertal, Wuppertal, Germany; 227 Department of Physics, Yale University, New Haven, CT USA; 228 Yerevan Physics Institute, Yerevan, Armenia; 229 Centre de Calcul de l’Institut National de Physique Nucléaire et de Physique des Particules (IN2P3), Villeurbanne, France; 230 CERN, 1211 Geneva 23, Switzerland

## Abstract

The reconstruction of the signal from hadrons and jets emerging from the proton–proton collisions at the Large Hadron Collider (LHC) and entering the ATLAS calorimeters is based on a three-dimensional topological clustering of individual calorimeter cell signals. The cluster formation follows cell signal-significance patterns generated by electromagnetic and hadronic showers. In this, the clustering algorithm implicitly performs a topological noise suppression by removing cells with insignificant signals which are not in close proximity to cells with significant signals. The resulting *topological cell clusters* have shape and location information, which is exploited to apply a local energy calibration and corrections depending on the nature of the cluster. Topological cell clustering is established as a well-performing calorimeter signal definition for jet and missing transverse momentum reconstruction in ATLAS.

## Introduction

The detectable final state emerging from the proton–proton collisions at the Large Hadron Collider (LHC) consists of particles and jets which are reconstructed with high precision for physics analyses. In the ATLAS experiment [1], clusters of topologically connected calorimeter cell signals (topo-clusters) are employed as a principal signal definition for use in the reconstruction of the (hadronic) part of the final state comprising isolated hadrons, jets and hadronically decaying $\tau$-leptons. In addition, topo-clusters are also used to represent the energy flow from softer particles, which is needed for the reconstruction of full-event observables such as the missing transverse momentum.

The algorithm building the topo-clusters explores the spatial distribution of the cell signals in all three dimensions to establish connections between neighbours in an attempt to reconstruct the energy and directions of the incoming particles. The signals from cells determined to be connected are summed, and are used together with the cell locations to calculate direction, location, and shapes of the resulting clusters. Calorimeter cells with insignificant signals found to not be connected to neighbouring cells with significant signals are considered noise and discarded from further jet, particle and missing transverse momentum reconstruction.

The topo-clusters, while well established in deep inelastic scattering experiments such as H1 [2] at HERA and in electron–positron collider experiments such as ALEPH [3] at LEP and BaBar [4] at PEP-II, are used here in an innovative implementation as fully calibrated three-dimensional objects representing the calorimeter signals in the complex final-state environment of hadron–hadron collisions. A similar application in this particular environment, previously developed by the D0 Collaboration, implements the topological clustering in the two dimensions spanned by pseudorapidity and the azimuthal angle, thus applying the noise-suppression strategy inherent in this algorithm for jet reconstruction [5]. Several features and aspects of the ATLAS topo-cluster algorithms and their validations have previously been presented in Refs. [6–9].

Some of the complexity of the final state in hadron–hadron collisions is introduced by particles from the underlying event generated by radiation and multiple parton interactions in the two colliding hadrons producing the hard-scatter final state. Other detector signal contributions from the collision environment, especially important for higher intensity operations at the LHC, arise from pile-up generated by diffuse particle emissions produced by the additional proton–proton collisions occurring in the same bunch crossing as the hard-scatter interaction (in-time pile-up). Further pile-up influences on the signal are from signal remnants from the energy flow in other bunch crossings in the ATLAS calorimeters (out-of-time pile-up).

This paper first describes the ATLAS detector in Sect. 2, together with the datasets used for the performance evaluations. The motivations and basic implementation of the topo-cluster algorithm are presented in Sect. 3. The computation of additional variables associated with topo-clusters including geometric and signal moments is described in Sect. 4. The various signal corrections applied to topo-clusters in the context of the local hadronic calibration are presented in Sect. 5. Section 6 summarises the performance of the topo-cluster signal in the reconstruction of isolated hadrons and jets produced in the proton–proton collisions at LHC. Performance evaluations with and without pile-up are discussed in this section, together with results from the corresponding Monte Carlo (MC) simulations. The paper concludes with a summary and outlook in Sect. 7.

## The ATLAS experiment

In this section the basic systems forming the ATLAS detector are described in Sect. 2.1, followed in Sect. 2.2 by a description of the datasets considered in this paper and the corresponding run conditions in data. The MC simulation setup for final-state generation and the simulation of the calorimeter response to the incident particles is described in Sect. 2.3.

### The ATLAS detector

The ATLAS experiment features a multi-purpose detector system with a forward–backward symmetric cylindrical geometry. It provides nearly complete and hermetic coverage of the solid angle around the proton–proton collisions at the LHC. A detailed description of the ATLAS experiment can be found in Ref. [1].

#### The ATLAS detector systems

The detector closest to the proton–proton collision vertex is the inner tracking detector (ID). It has complete azimuthal coverage and spans the pseudorapidity^1^ region $|\eta |<2.5$. It consists of a silicon pixel detector, a silicon micro-strip detector, and a straw-tube transition radiation tracking detector covering $|\eta |<2$. The ID is immersed into a uniform axial magnetic field of $2\,\text {T}$ provided by a thin superconducting solenoid magnet.

**Fig. 1:**
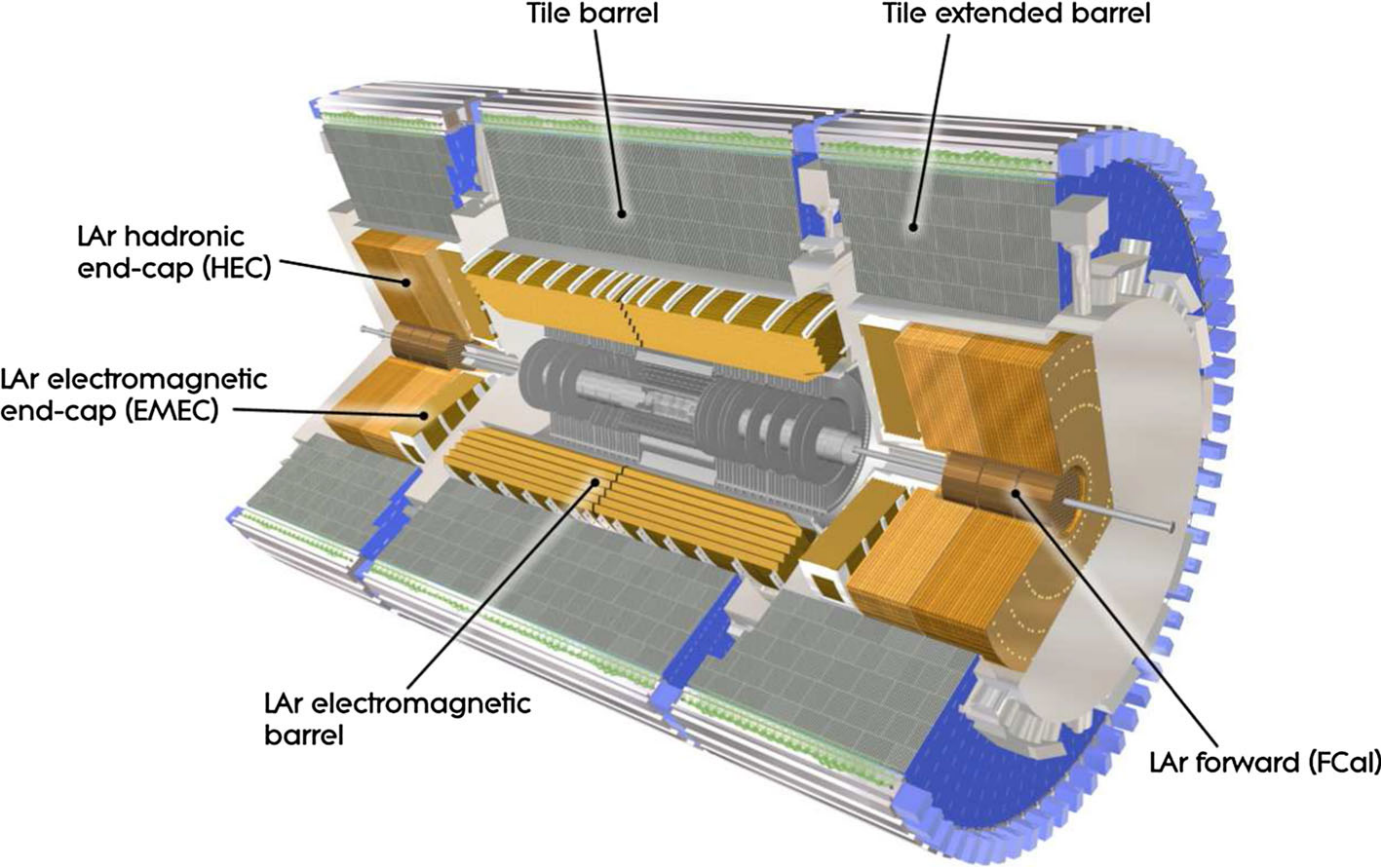
Cutaway view on the ATLAS calorimeter system

The ATLAS calorimeter system is illustrated in Fig. 1. It comprises several calorimeters with various read-out granularities and with different technologies. The electromagnetic calorimeter (EM) surrounding the ID is a high-granularity liquid-argon sampling calorimeter (LAr), using lead as an absorber. It is divided into one barrel (EMB; $|\eta |<1.475$) and two end-cap (EMEC; $1.375<|\eta |<3.2$) regions.

The barrel and end-cap regions also feature pre-samplers mounted between the cryostat cold wall and the calorimeter modules. The barrel pre-sampler (PreSamplerB) covers $|\eta | < 1.52$, while the end-cap pre-sampler (PreSamplerE) covers $1.5< |\eta | < 1.8$.

The hadronic calorimeters are divided into three distinct sections. The most central section contains the central barrel region ($|\eta |<0.8$) and two extended barrel regions ($0.8<|\eta |<1.7$). These regions are instrumented with scintillator-tile/steel hadronic calorimeters (Tile). Each barrel region consists of 64 modules with individual azimuthal ($\phi$) coverages of $\pi /32$ rad. The two hadronic end-cap calorimeters (HEC; $1.5<|\eta |<3.2$) feature liquid-argon/copper calorimeter modules. The two forward calorimeters (FCAL; $3.1<|\eta |<4.9$) are instrumented with liquid-argon/copper and liquid-argon/tungsten modules for electromagnetic and hadronic energy measurements, respectively.

The ATLAS calorimeters have a highly granular lateral and longitudinal segmentation. Including the pre-samplers, there are seven sampling layers in the combined central calorimeters (PreSamplerB, three in EMB and three in Tile) and eight sampling layers in the end-cap region (PreSamplerE, three in EMEC and four in HEC). The three FCal modules provide three sampling layers in the forward region. Altogether, the calorimeter system has about $188\,000$ read-out channels. The EM calorimeters are between 24 radiation lengths ($X_{0}$) and $27\,X_{0}$ deep. The combined depth of the calorimeters for hadronic energy measurements is more than 10 hadronic interaction lengths ($\lambda$) nearly everywhere across the full detector acceptance ($|\eta | \le 4.9$). The amount of inactive material in front of the calorimeters depends on $\eta$. It varies from about $2\,X_{0}$ at $\eta = 0$ to about $4\,X_{0}$ at $|\eta | \approx 1.8$, when measured from the nominal interaction point in ATLAS to the first active sampling layer (including PreSamplerB and PreSamplerE). It can increase to more than $6\,X_{0}$ in the transition region between central and end-cap calorimeters ($|\eta | \approx 1.45$ and $|\eta | \approx 1.7$). The amount of inactive material for hadrons is approximately $1\,\lambda$ across the full covered $\eta$-range, with spikes going up to more than $2\,\lambda$ in transition regions and in regions with complex cryostat structures and beam line services ($|\eta | \approx 4$).

The absorption power of the ATLAS calorimeters and their segmentation allow for very precise energy-flow reconstruction based on the topo-clusters described in this paper, with considerable exploitation of the topo-cluster shapes for signal calibration purposes. For more details of the calorimeter read-out structures, absorption characteristics, inactive material distributions, and cell signal formation, see Ref. [1]. The segmentation of the read-out structure in the various calorimeter sampling layers, each named by a dedicated identifier ($S_{\text {calo}}$), is shown in Table 1.

**Table 1:** The read-out granularity of the ATLAS calorimeter system [1], given in terms of $\Delta \eta \times \Delta \phi$ with the exception of the forward calorimeters, where it is given in linear measures $\Delta x \times \Delta y$, due to the non-pointing read-out geometry of the FCAL. For comparison, the FCAL granularity is approximately $\Delta \eta \times \Delta \phi = 0.15 \times 0.15 (0.3 \times 0.3)$ at $\eta = 3.5(4.5)$. The total number of read-out cells, including both ends of the calorimeter system, with (without) pre-samplers is $187\,652$ (178,308)

Calorimeter	Module sampling ($S_{\text {calo}}$)	$N_{\text {cells}}$	$\eta$-coverage	$\Delta \eta \times \Delta \phi$
*Electromagnetic calorimeters*	EMB	109,568	$|\eta |<1.52$	
	PreSamplerB	7808	$|\eta |<1.52$	$0.025\times \pi /32$
	EMB1		$|\eta |<1.4$	$0.025/8\times \pi /32$
			$1.4<|\eta |<1.475$	$0.025\times \pi /128$
	EMB2		$|\eta |<1.4$	$0.025\times \pi /128$
			$1.4<|\eta |<1.475$	$0.075\times \pi /128$
	EMB3		$|\eta |<1.35$	$0.050\times \pi /128$
	EMEC	63,744	$1.375< |\eta | < 3.2$	
	PreSamplerE	1536	$1.5<|\eta |<1.8$	$0.025\times \pi /32$
	EME1		$1.375<|\eta |<1.425$	$0.050\times \pi /32$
			$1.425<|\eta |<1.5$	$0.025\times \pi /32$
			$1.5<|\eta |<1.8$	$0.025/8\times \pi /32$
			$1.8<|\eta |<2.0$	$0.025/6\times \pi /32$
			$2.0<|\eta |<2.4$	$0.025/4\times \pi /32$
			$2.4<|\eta |<2.5$	$0.025\times \pi /32$
			$2.5<|\eta |<3.2$	$0.1\times \pi /32$
	EME2		$1.375<|\eta |<1.425$	$0.050\times \pi /128$
			$1.425<|\eta |<2.5$	$0.025\times \pi /128$
			$2.5<|\eta |<3.2$	$0.1\times \pi /128$
	EME3		$1.5<|\eta |<2.5$	$0.050\times \pi /128$
*Hadronic calorimeters*	Tile (barrel)	2880	$|\eta |<1$	
	TileBar0/1			$0.1\times \pi /32$
	TileBar2			$0.2 \times \pi /32$
	Tile (extended barrel)	2304	$0.8< |\eta | < 1.7$	
	TileExt0/1			$0.1\times \pi /32$
	TileExt2			$0.2\times \pi /32$
	HEC	5632	$1.5<|\eta |<3.2$	
	HEC0/1/2/3		$1.5<|\eta |<2.5$	$0.1\times \pi /32$
			$2.5<|\eta |<3.2$	$0.2\times \pi /16$
*Forward calorimeters*	FCAL	3524	$3.1<|\eta |<4.9$	$\Delta x \times \Delta y$
	FCAL0		$3.1<|\eta |<3.15$	$1.5\,{\text {cm}}\times 1.3\,{\text {cm}}$
			$3.15<|\eta |<4.3$	$3.0\,{\text {cm}}\times 2.6\,{\text {cm}}$
			$4.3<|\eta |<4.83$	$1.5\,{\text {cm}}\times 1.3\,{\text {cm}}$
	FCAL1		$3.2<|\eta |<3.24$	$1.7\,{\text {cm}}\times 2.1\,{\text {cm}}$
			$3.24<|\eta |<4.5$	$3.3\,{\text {cm}}\times 4.2\,{\text {cm}}$
			$4.5<|\eta |<4.81$	$1.7\,{\text {cm}}\times 2.1\,{\text {cm}}$
	FCAL2		$3.29<|\eta |<3.32$	$2.7\,{\text {cm}}\times 2.4\,{\text {cm}}$
			$3.32<|\eta |<4.6$	$5.4\,{\text {cm}}\times 4.7\,{\text {cm}}$
			$4.6<|\eta |<4.75$	$2.7\,{\text {cm}}\times 2.4\,{\text {cm}}$

The muon spectrometer surrounds the ATLAS calorimeters. A system of three large air-core toroids, a barrel and two end-caps with eight coils each, generates a magnetic field in the pseudorapidity range of $|\eta | < 2.7$. The muon spectrometer measures the full momentum of muons based on their tracks reconstructed with three layers of precision tracking chambers in the toroidal field. It is also instrumented with separate trigger chambers.

#### The ATLAS trigger

The trigger system for the ATLAS detector in Run 1 consisted of a hardware-based Level 1 (L1) trigger and a software-based High Level Trigger (HLT) [10]. For the evaluation of the topo-cluster reconstruction performance, samples of minimum-bias (MB) triggered events, samples of events selected by jet triggers, and samples of events with hard objects such as muons, which are not triggered by the calorimeter, are useful.

The ATLAS MB trigger [11] used signals from a dedicated system of scintillators (MBTS [12]; $2.1< |\eta | < 3.8$) at L1 in 2010 and 2011 data-taking. Depending on the run period, it required one hit in either of the $\eta$ hemispheres, or one hit in each $\eta$ hemisphere. In 2012, the MB samples were triggered by a zero-bias trigger. This trigger unconditionally accepted events from bunch crossings occurring a fixed number of LHC cycles after a high-energy electron or photon was accepted by the L1 trigger. The L1 trigger rate for these hard objects scales linearly with luminosity, thus the collision environment generated by the luminosity-dependent additional proton–proton interactions discussed in Sect. 2.2.1 is well reflected in the MB samples.

For triggering on collision events with jets at L1, jets are first built from coarse-granularity calorimeter towers using a sliding-window algorithm (L1-jets). The events are accepted if they have L1-jets passing triggers based on (1) the transverse momentum ($p_{\text {T}}$) of individual L1-jets (single-jet triggers) or on (2) the detection of several such jets at increasing transverse momenta (multi-jet triggers). Those events accepted by L1 are then subjected to refined jet-trigger decisions based on jet $p_{\text {T}}$ and multi-jet topology in the HLT, now using jets that are reconstructed from calorimeter cell signals with algorithms similar to the ones applied in the offline precision reconstruction [13].

A $Z$ boson sample is collected from muon triggers at L1. Since the trigger rate and the reconstruction of the decay properties of the accepted $Z \!\rightarrow \!\mu \mu$ events are basically unaffected by pile-up, this sample is not only unbiased in this respect but also with respect to other possible biases introduced by the ATLAS calorimeter signals.

### Dataset

**Fig. 2:**
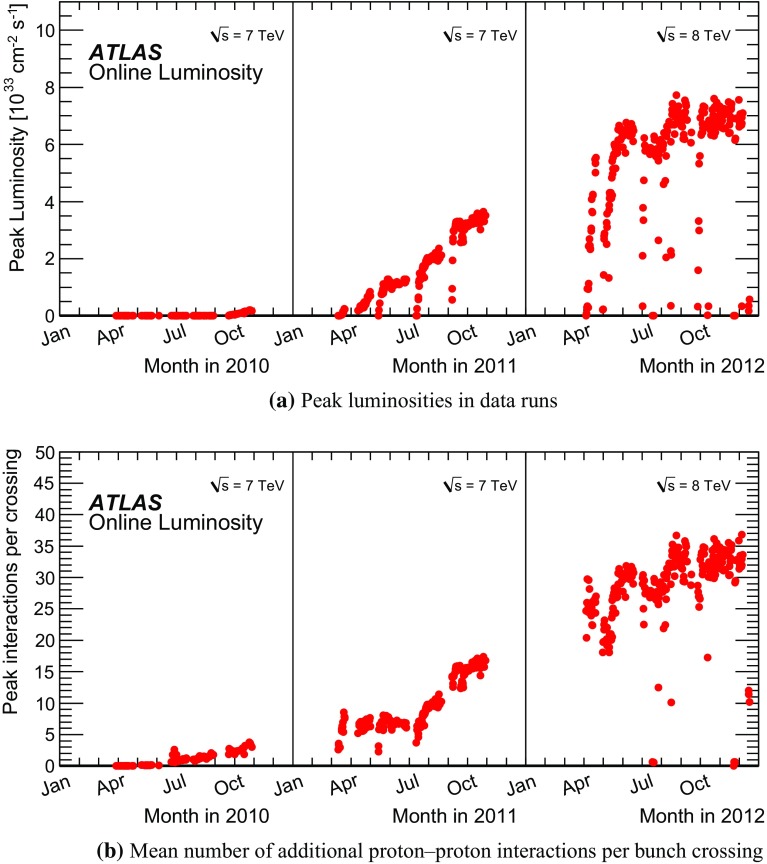
The peak luminosities measured by the ATLAS online luminosity monitor system throughout the run years are shown in (**a**). The mean number of additional proton–proton interactions at the beginning of each LHC fill is shown in (**b**) for the same period in time

The data used for the evaluation of the topo-cluster reconstruction performance are selected from proton–proton collision events at a centre-of-mass energy of $\sqrt{s} = 7\,{\text {TeV}}$, recorded with the ATLAS detector in 2010, and at $\sqrt{s} = 8\,{\text {TeV}}$ in 2012. The overall amount of high-quality data recorded at those times corresponds to $\sim \!45\,\text {pb}^{-1}$ in 2010, and $\sim \!20.3\,\text {fb}^{-1}$ in 2012. Peak instantaneous luminosities reached in the first three years of LHC running (LHC Run 1) are shown in Fig. 2a. Some early data recorded during the very first proton–proton collisions in the LHC in 2009 are considered for the studies of the topo-cluster reconstruction performance as well. The corresponding events are extracted from approximately $540\,000$ proton–proton collisions at $\sqrt{s} = 900\,{\text {GeV}}$, recorded during stable beam conditions and corresponding to about $12\,\text {mb}^{-1}$. Occasional references to 2011 run conditions, where protons collided in the LHC with $\sqrt{s} = 7\,{\text {TeV}}$ and ATLAS collected data corresponding to $\sim \!5.1\,\text {fb}^{-1}$, are provided to illustrate the evolution of the operational conditions during LHC Run 1 relevant to topo-cluster reconstruction. The specific choice of 2010 and 2012 data for the performance evaluations encompasses the most important scenarios with the lowest and highest luminosity operation, respectively.

#### Pile-up in data

One important aspect of the contribution from additional proton–proton interactions (pile-up) to the calorimeter signal in data is the sensitivity of the ATLAS liquid-argon calorimeters to this pile-up as a function of the instantaneous luminosity, and as a function of the signal history from previous bunch crossings.

**Fig. 3:**
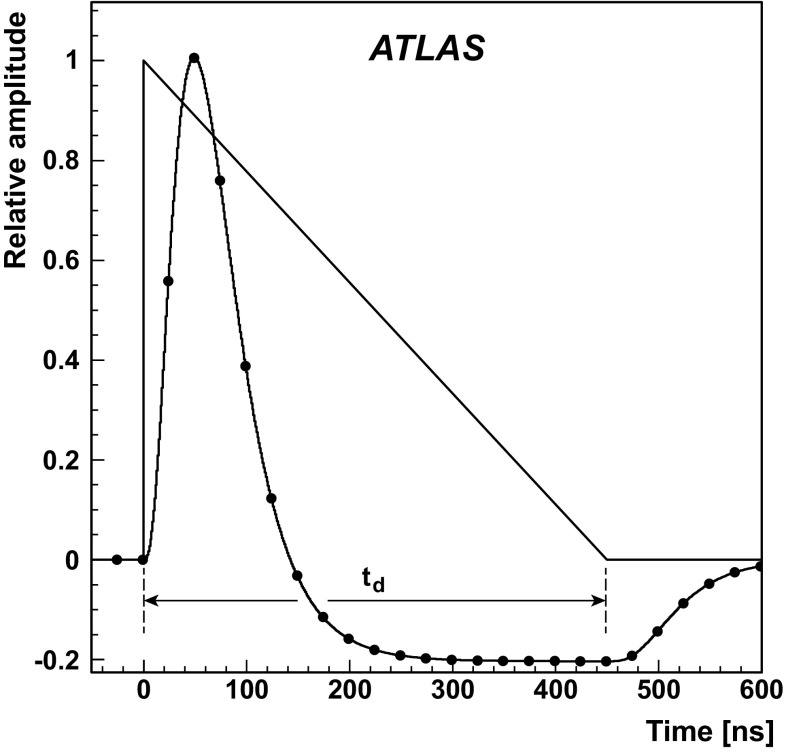
The pulse shape in the ATLAS LAr calorimeters. The unipolar triangular pulse is the current pulse in the liquid argon generated by fast ionising particles. Its characteristic time is the drift time (charge collection time) $t_{\text {d}}$, with $t_{\text {d}} \approx 450\,{\text {ns}}$ in the example for the central EMB calorimeter shown here. The shaped pulse is superimposed, with a characteristic duration of $\tau _{\text {signal}} \approx 600\,{\text {ns}}$. The *full circles* on the shaped pulse indicate the nominal bunch crossings at $25\,{\text {ns}}$ intervals. The figure has been adapted from Ref. [14]

In the initial phase of data-taking in 2010 the proton beam intensities at LHC were relatively low. The recorded events contain on average three additional proton–proton interactions, as shown in Fig. 2b. In addition, the initial bunch crossing interval of $t_{\text {BX}} = 750\,{\text {ns}}$ was larger than the window of sensitivity of the LAr calorimeter, which is defined by the duration $\tau _{\text {signal}}$ of the shaped signal, with $\tau _{\text {signal}} \approx 600\,{\text {ns}}$, as depicted in Fig. 3 for the typical charge collection time of $t_{\text {d}} = 450\,{\text {ns}}$ in this detector. In later data-taking periods in 2010 the bunch crossing interval was reduced to $t_{\text {BX}} = 175\,{\text {ns}}$, which is within the sensitivity of the LAr calorimeter signal formation ($t_{\text {BX}} < \tau _{\text {signal}}$). Nevertheless, the still-low instantaneous luminosity reduced the amount of energy scattered into the calorimeter in the other bunch crossings to a negligible contribution with little effect on the signal history.

Throughout operations in 2011 and 2012, the proton beam intensities in the LHC were significantly increased, leading to the corresponding increases in the number of pile-up interactions per bunch crossing shown in Fig. 2(b). At the same time, $t_{\text {BX}}$ was reduced to $50\,{\text {ns}}$. These two changes in the run conditions introduced a sensitivity of the LAr calorimeter signal to the signal residuals from proton–proton interactions occurring in $N_{\text {BX}}^{\text {PU}} \approx 12$ preceding bunch crossings at the LHC (out-of-time pile-up), in addition to pile-up interactions in the current bunch crossing (in-time pile-up). The out-of-time pile-up effect on the cell signal depends on $N_{\text {BX}}^{\text {PU}} \approx \tau _{\text {signal}}/t_{\text {BX}}$ and the energy deposited in each of the $N_{\text {BX}}^{\text {PU}}$ bunch crossings.

The bipolar shape of the LAr calorimeter signal shown in Fig. 3 reduces the overall effect of pile-up, because it features a net-zero integral over time. This leads to cancellation on average of in-time pile-up signal contributions by out-of-time pile-up signal residuals in any given calorimeter cell. By design of the shaping amplifier, and the choice of digitally sampling the shaped pulse amplitude in time with a frequency of $40\,\text {MHz}$ in the read-out, the most efficient suppression is achieved for $25\,{\text {ns}}$ bunch spacing in the LHC beams. It is fully effective in the limit where for each bunch crossing contributing to out-of-time pile-up about the same amount of energy is deposited in a given calorimeter cell. A small loss of efficiency is observed for $50\,{\text {ns}}$ bunch spacing, due to the less frequent injection of energy by the fewer previous bunch crossings.

Approximately the first ten bunch crossings in each LHC bunch train at $50\,{\text {ns}}$ bunch spacing are characterised by different out-of-time pile-up contributions from the collision history. This history gets filled with signal remnants from an increasing number of past bunch crossings with proton–proton interactions the larger the time difference between the bunch crossing and the beginning of the train becomes. The remaining bunch crossings in a train, about 26 of a total of 36 in 2011 and 62 of a total of 72 in 2012, have an out-of-time pile-up signal contribution which is stable within the bunch-to-bunch fluctuations in the beam intensity. In 2012 data a dedicated cell-by-cell correction is applied in the offline cell signal reconstruction to compensate for the corresponding variations in the out-of-time pile-up. Further details of the ATLAS liquid-argon calorimeter read-out and signal processing can be found in Ref. [15].

Even with a constant proton bunch intensity and apart from the bunch train effects, the efficiency of pile-up suppression by signal shaping is reduced by the large fluctuations in the number of additional interactions from bunch crossing to bunch crossing, and by the different energy-flow patterns of the individual collisions in the time window of sensitivity $\tau _{\text {signal}}$ in the LAr calorimeters. Consequently, the signal shows a principal sensitivity to pile-up, even after shaping and digital filtering in the read-out. This is evident from the residual event-by-event deviation of the cell-signal baseline, which depends on the specific pile-up condition at the time of the triggered event, from the (average zero) baseline expected from the signal shaping. These baseline fluctuations can lead to relevant signal offsets once the noise suppression is applied, which is an important part of the calorimeter signal extraction strategy using topo-clusters presented in Sect. 3.

The Tile calorimeter shows very little sensitivity to pile-up since most of the associated (soft particle) energy flow is absorbed in the LAr calorimeters in front of it. Moreover, out-of-time pile-up is suppressed by a shorter signal collection time and a short pulse shaping time, reducing the sensitivity of the signal to only about three bunch crossings at $50\,{\text {ns}}$ intervals [12].

#### Effect on calorimeter noise

**Fig. 4:**
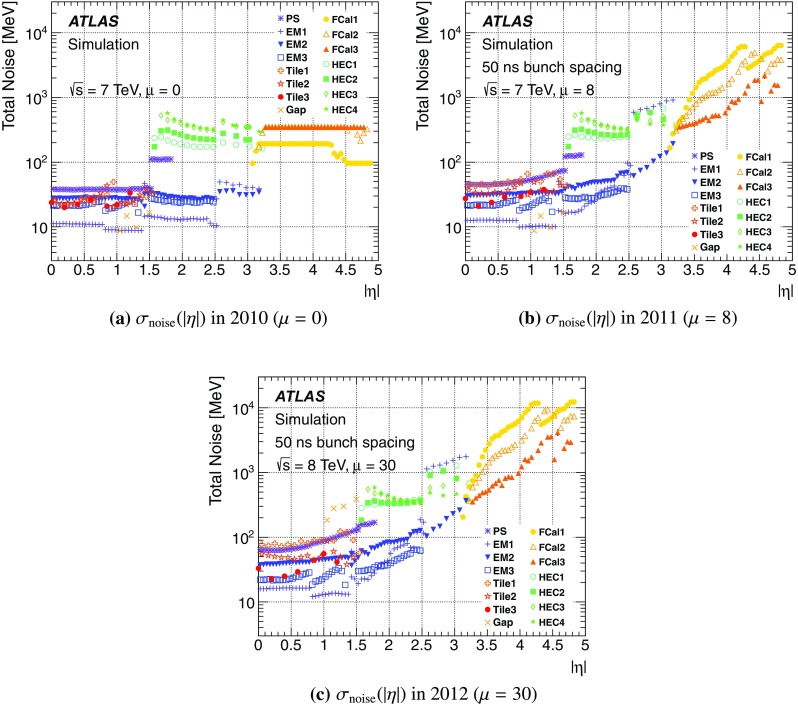
The energy-equivalent cell noise in the ATLAS calorimeters on the electromagnetic (EM) scale as a function of the direction $\left| \eta \right|$ in the detector, for **a** the 2010 configuration with $\mu = 0$, **b** the 2011 configuration with $\mu = 8$ (both plots from Ref. [16]), and **c** the 2012 configuration with $\mu = 30$. The various *colours* indicate the noise in the pre-sampler (PS) and the three layers of the LAr EM calorimeter, the three layers of the Tile calorimeter, the four layers of the hadronic end-cap (HEC) calorimeter, and the three modules of the forward (FCAL) calorimeter

In ATLAS operations prior to 2011 the cell noise was dominated by electronic noise. The short bunch crossing interval and higher instantaneous luminosity in 2011 and 2012 LHC running added additional and dominant noise contributions from the cell-signal baseline fluctuations introduced by pile-up, as discussed in Sect. 2.2.1. These fluctuations, even though not perfectly following a Gaussian distribution,^2^ can nevertheless be expressed as noise measured by the standard deviation of their distribution, taken from simulated MB events and scaled to the expected number of pile-up interactions. The cell noise thresholds steering the topo-cluster formation described in Sect. 3 thus needed to be increased from those used in 2010 to accommodate this pile-up-induced noise. This is done by adjusting the nominal energy-equivalent noise $\sigma _{\text {noise}}$ according to

1 $$\begin{aligned} \sigma _{\text {noise}}= \left\{ \begin{array}{lr} \sigma _{\text {noise}}^{\text {electronic}}&{}\text {(2010 operations)}, \\ \sqrt{\left( \sigma _{\text {noise}}^{\text {electronic}}\right) ^{2} + \left( \sigma _{\text {noise}}^{\mathrm{pile-up}}\right) ^{2}} &{}\text {(2011 and 2012 operations)}. \end{array}\right. \end{aligned}$$

Here, $\sigma _{\text {noise}}^{\text {electronic}}$ is the electronic noise, and $\sigma _{\text {noise}}^{\mathrm{pile-up}}$ the noise from pile-up, corresponding to an average of eight additional proton–proton interactions per bunch crossing ($\mu = 8$) in 2011, and $\mu = 30$ in 2012. These configurations are choices based on the expected average $\langle \mu \rangle$ for the run year. They needed to be made before the respective data-taking started, to allow for a fast turn-around reconstruction of the collected data. As $\mu$ changes with the decrease of the instantaneous luminosity $L_{\text {inst}}$ through-out the LHC proton fill, $\sigma _{\text {noise}}^{\mathrm{pile-up}}$ is only optimal for the small subset of data recorded when $L_{\text {inst}}$ generated the nominal (*a priori* chosen) $\mu$ pile-up interactions on average. LHC operations at lower $\mu$ lead to slightly reduced calorimeter sensitivity to relevant small signals, as $\sigma _{\text {noise}}^{\mathrm{pile-up}}$ is too large. For data-taking periods with higher than nominal $\mu$ the noise suppression is not optimal, leading to more noise contributions to the topo-cluster signals.

The change of the total nominal noise $\sigma _{\text {noise}}$ and its dependence on the calorimeter region in ATLAS can be seen by comparing Fig. 4a–c. In most calorimeter regions, the total noise rises significantly above the electronic noise with increasing pile-up activity, as expected. This increase is largest in the forward calorimeters, where $\sigma _{\text {noise}}^{\mathrm{pile-up}}\gg \sigma _{\text {noise}}^{\text {electronic}}$ by more than one order of magnitude, already under 2011 run conditions.

### Monte Carlo simulations

The energy and direction of particles produced in proton–proton collisions are simulated using various MC event generators. An overview of these generators for LHC physics can be found in Ref. [17]. The samples for comparisons to 2010 data are produced at $\sqrt{s} = 7\,{\text {TeV}}$, while the MC samples for 2012 analyses are generated at $\sqrt{s} = 8\,{\text {TeV}}$. Some configuration details for the inclusive jet and inclusive $Z$ boson MC samples and the simulated MB samples are given below.

#### Monte Carlo simulations of signal samples

Simulated signal samples include inclusive jet-production, which is generated using Pythia [18] version 6.425 for 2010 analyses, and Pythia8 [19] version 8.160 for 2012 analysis. Both generators model the hard sub-process in the final states of the generated proton–proton collisions using a $2\!\!\rightarrow \!\!2$ matrix element at leading order in the strong coupling $\alpha _{\text {S}}$. Additional radiation is modelled in the leading-logarithmic (LL) approximation by $p_{\text {T}}$-ordered parton showers [20]. Multiple parton interactions (MPI) [21], as well as fragmentation and hadronisation based on the Lund string model [22], are also generated.

For comparisons with 2012 data, samples of $Z$ bosons with $Z \!\rightarrow \!\mu \mu$ are generated. The next-to-leading-order (NLO) POWHEG [23, 24] model is used, with the final-state partons showered by Pythia8 using the CT10 NLO parton distribution function (PDF) [25] and the ATLAS AU2 [26] set of tuned parton shower and other soft underlying event generation parameters. Pythia8 also provides the MPI, fragmentation and hadronisation for these events.

#### Minimum-bias samples and pile-up modelling

The MB samples for 2012 running conditions are generated using Pythia8 with the ATLAS AM2 [26] set of tuned soft interaction parameters and the MSTW2008LO PDF set [27]. A single, fully simulated event for that run year is built by overlaying a number $N_{\text {PU}}$ of generated MB events onto one generated hard-scatter event. The actual $N_{\text {PU}}$ is drawn from a Poisson distribution around the average number $\langle \mu \rangle$ of additional proton–proton collisions per bunch crossing. The value of $\langle \mu \rangle$ is measured by the experiment as an average over one *luminosity block*, which can last as long as two minutes, with its actual duration depending on the central data acquisition configuration at the time of the data-taking. The measurement of $\langle \mu \rangle$ is mainly based on single $\eta$-hemisphere hit counting as well as counting coincidental hits in both $\eta$-hemispheres with the fast ATLAS luminosity detectors consisting of two small Cherenkov counter (LUCID; $5.6< |\eta | < 6.0$) and two sets of small diamond sensors forming two beam conditions monitors (BCM; $|\eta | = 4.2$). Details of these detectors and the measurement are given in Ref. [28]. The distribution of the measured $\langle \mu \rangle$ over the whole run period is taken into account in the pile-up simulation.

The LHC bunch train structure with 72 proton bunches per train and $50\,{\text {ns}}$ spacing between the bunches in 2012, is also modelled by organising the simulated collisions into four such trains. This allows the inclusion of out-of-time pile-up effects driven by the distance of the hard-scatter events from the beginning of the bunch train, as discussed in Sect. 2.2.1. A correction depending on the bunch position in the train is applied to data and MC simulations to mitigate these effects. Bunch-to-bunch intensity fluctuations in the LHC are not included in the MC modelling. These are corrected in the data by the correction depending on the position of the bunch in the train.

#### Minimum-bias overlay samples for 2012

In addition to the fully generated and simulated MC samples described earlier, samples with events mixing data and MC simulations are used to study the topo-cluster reconstruction performance. These samples are produced by overlaying one event from the MB samples collected by the zero-bias trigger described in Sect. 2.1.2 and a hard-scatter interaction from the MC generator [29–31]. The generated hard-scatter event is simulated using the detector simulation described in Sect. 2.1, but without any noise effects included. The recorded and simulated raw electronic signals are then overlaid prior to the digitisation step in the simulation. This results in modelling both the detector noise and the effect of pile-up from data with the correct experimental conditions on top of the simulated event. Theses samples are useful for detailed comparisons of topo-cluster signal features in 2012, as they do not depend on limitations in the soft-event modelling introduced by any of the generators.

#### Detector simulation

The Geant4 software toolkit [32] within the ATLAS simulation framework [33] propagates the stable particles^3^ produced by the event generators through the ATLAS detector and simulates their interactions with the detector material and the signal formation. Hadronic showers are simulated with the quark–gluon-string-plasma model employing a quark–gluon string model [34] at high energies and the Bertini intra-nuclear cascade model [35–37] at low energies (QGSP_BERT). There are differences between the detector simulation used in 2010 and in 2012. A newer version of Geant4 (version 9.4) is employed in 2012, together with a more detailed description of the LAr calorimeter absorber structure. These geometry changes introduce an increase of about 2% in the calorimeter response to pions with energies of less than $10\,{\text {GeV}}$.

### Hadronic final-state reconstruction in ATLAS

The fully reconstructed final state of the proton–proton collisions in ATLAS includes identified individual particles comprising electrons, photons, muons, and $\tau$-leptons, in addition to jets and missing transverse momentum ($E_{\text {T}}^{\text {miss}}$). Calorimeter signals contribute to all objects, except for muons. The topo-clusters introduced in detail in Sect. 3 are primarily used for the reconstruction of isolated hadrons, jets and $E_{\text {T}}^{\text {miss}}$.

Jets are reconstructed using topo-clusters, with their energies either reconstructed on the basic (electromagnetic) scale presented in Sect. 3.2, or on the fully calibrated and corrected (hadronic) scale described in Sect. 5.

Additional refinement of the jet energy scale (JES) may include reconstructed charged-particle tracks from the ID. More details of jet reconstruction and calibration can be found in Refs. [16, 38].

Jets used in the studies presented here are reconstructed in data and MC simulations using the anti-$k_{t}$ jet algorithm [39] as implemented in the FastJet package [40]. The jet size is defined by the radius parameter *R* in the jet algorithm, where both $R = 0.4$ and $R = 0.6$ are used. Full four-momentum recombination is used, restricting the input topo-cluster signals to be positive for a meaningful jet formation. The jets are fully calibrated and corrected after formation, including a correction for pile-up signal contributions. For 2012, the pile-up correction employs the reconstructed median transverse momentum density in the event and the area of the jet to subtract the $p_{\text {T}}$ contribution from pile-up, following the suggestions in Ref. [41]. In addition, an MC simulation-based residual correction is applied [42].

## Topological cluster formation and features

The collection of the calorimeter signals of a given collision event into clusters of topologically connected cell signals is an attempt to extract the significant signal from a background of electronic noise and other sources of fluctuations such as pile-up. This strategy is most effective in a highly granular calorimeter system such as the one employed by ATLAS. Finely segmented lateral read-out together with longitudinal sampling layers allows the resolution of energy-flow structures generating these spatial signal patterns, thus retaining only signals important for particle and jet reconstruction while efficiently removing insignificant signals induced by noise. The signal extraction is guided by reconstructing three-dimensional “energy blobs” from particle showers in the active calorimeter volume. Individual topo-clusters are not solely expected to contain the entire response to a single particle all of the time. Rather, depending on the incoming particle types, energies, spatial separations and cell signal formation, individual topo-clusters represent the full or fractional response to a single particle (full shower or shower fragment), the merged response of several particles, or a combination of merged full and partial showers.

### Topo-cluster formation

The collection of calorimeter cell signals into topo-clusters follows spatial signal-significance patterns generated by particle showers. The basic observable controlling this cluster formation is the cell signal significance $\varsigma _{\text {cell}}^{\,{{\text {EM}}}}$, which is defined as the ratio of the cell signal to the average (expected) noise $\sigma _{\text {noise,cell}}^{\,{{\text {EM}}}}$ in this cell, as estimated for each run year according to Eq. (1) (with $\sigma _{\text {noise,cell}}^{\,{{\text {EM}}}}= \sigma _{\text {noise}}$),

2 $$\begin{aligned} \varsigma _{\text {cell}}^{\,{{\text {EM}}}}= \frac{E_{\text {cell}}^{{{\text {EM}}}}}{\sigma _{\text {noise,cell}}^{\,{{\text {EM}}}}} . \end{aligned}$$

Both the cell signal $E_{\text {cell}}^{{{\text {EM}}}}$ and $\sigma _{\text {noise,cell}}^{\,{{\text {EM}}}}$ are measured on the electromagnetic (EM) energy scale. This scale reconstructs the energy deposited by electrons and photons correctly but does not include any corrections for the loss of signal for hadrons due to the non-compensating character of the ATLAS calorimeters.

Topo-clusters are formed by a growing-volume algorithm starting from a calorimeter cell with a highly significant seed signal. The seeding, growth, and boundary features of topo-clusters are in this algorithm controlled by the three respective parameters $\{S,N,P\}$, which define signal thresholds in terms of $\sigma _{\text {noise,cell}}^{\,{{\text {EM}}}}$ and thus apply selections based on $\varsigma _{\text {cell}}^{\,{{\text {EM}}}}$ from Eq. (2),

3 $$\begin{aligned} \left| E_{\text {cell}}^{{{\text {EM}}}}\right|> S \sigma _{\text {noise,cell}}^{\,{{\text {EM}}}}&\Rightarrow \left| \varsigma _{\text {cell}}^{\,{{\text {EM}}}}\right| > S&(\text {primary seed threshold, default} S = 4); \end{aligned}$$

4 $$\begin{aligned} \left| E_{\text {cell}}^{{{\text {EM}}}}\right|> N \sigma _{\text {noise,cell}}^{\,{{\text {EM}}}}&\Rightarrow \left| \varsigma _{\text {cell}}^{\,{{\text {EM}}}}\right| > N&(\text {threshold for growth control, default } N = 2); \end{aligned}$$

5 $$\begin{aligned} \left| E_{\text {cell}}^{{{\text {EM}}}}\right|> P \sigma _{\text {noise,cell}}^{\,{{\text {EM}}}}&\Rightarrow \left| \varsigma _{\text {cell}}^{\,{{\text {EM}}}}\right| > P&(\text {principal cell filter, default } P = 0). \end{aligned}$$

Useful configurations employ a $S > N \ge P$ rule, as reflected in the default configuration for ATLAS indicated above. The default values are derived from optimisations of the response and the relative energy resolution for charged pions in test-beam experiments using ATLAS calorimeter prototypes [43].

#### Collecting cells into topo-clusters

Topo-cluster formation is a sequence of *seed and collect* steps, which are repeated until all topologically connected cells passing the criteria given in Eqs. (3) and (4) and their direct neighbours satisfying the condition in Eq. (5) are found. The algorithm starts by selecting all cells with signal significances $\varsigma _{\text {cell}}^{\,{{\text {EM}}}}$ passing the threshold defined by *S* in Eq. (3) from calorimeter regions which are allowed to seed clusters.^4^ These seed cells are then ordered in decreasing $\varsigma _{\text {cell}}^{\,{{\text {EM}}}}$.

Each seed cell forms a *proto-cluster*. The cells neighbouring a seed and satisfying Eqs. (4) or (5) are collected into the corresponding proto-cluster. Here *neighbouring* is generally defined as two calorimeter cells being directly adjacent in a given sampling layer, or, if in adjacent layers, having at least partial overlap in the $(\eta ,\phi )$ plane. This means that the cell collection for topo-clusters can span modules within the same calorimeter as well as calorimeter sub-detector transition regions. Should a neigbouring cell have a signal significance passing the threshold defined by the parameter *N* in Eq. (4), its neighbours are collected into the proto-cluster as well. If a particular neighbour is a seed cell passing the threshold *S* defined in Eq. (3), the two proto-clusters are merged. If a neighbouring cell is attached to two different proto-clusters and its signal significance is above the threshold defined by *N*, the two proto-clusters are merged. This procedure is iteratively applied to further neighbours until the last set of neighbouring cells with significances passing the threshold defined by *P* in Eq. (5), but not the one in Eq. (4), is collected. At this point the formation stops.

**Fig. 5:**
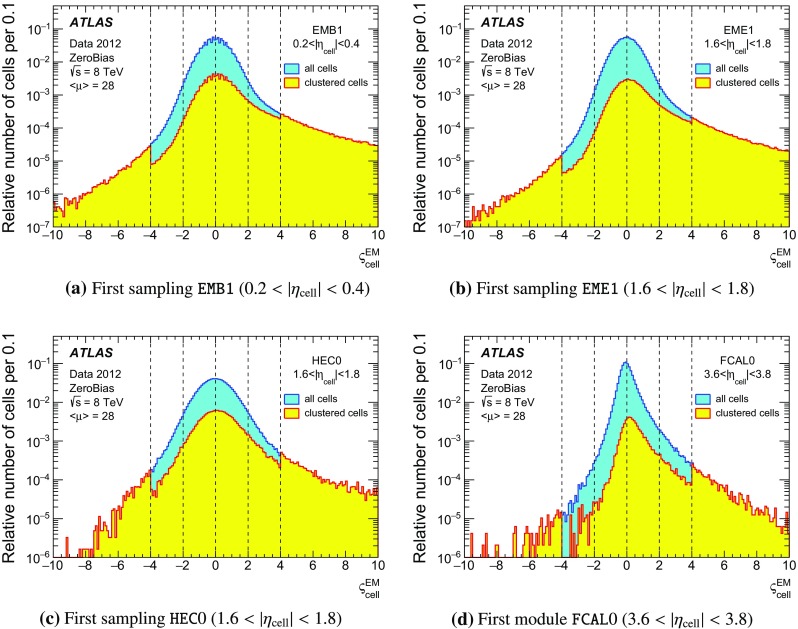
Signal significance ($\varsigma _{\text {cell}}^{\,{{\text {EM}}}}$) distributions for all cells (*blue*/*cyan*) and for cells after the noise suppression in the topological cell clustering is applied (*red*/*yellow*), in selected sampling layers of the LAr calorimeters: **a** the first sampling of the central electromagnetic LAr calorimeter (EMB), **b** the first sampling of the electromagnetic LAr end-cap calorimeter (EMEC), **c** the first sampling of the hadronic LAr end-cap calorimeter (HEC), and **d** the first module of the LAr forward calorimeter (FCAL). The spectra are extracted from 2012 zero-bias data at $\sqrt{s} = 8\,{\text {TeV}}$ with an average number of pile-up interactions $\langle \mu \rangle = 28$. The *dashed lines* indicate $S = \pm 4$, $N = \pm 2$, and $P = 0$

The resulting proto-cluster is characterised by a core of cells with highly significant signals. This core is surrounded by an envelope of cells with less significant signals. The configuration optimised for ATLAS hadronic final-state reconstruction is $S = 4$, $N = 2$, and $P = 0$, as indicated in Eqs. (3) to (5). This particular configuration with $P = 0$ means that any cell neighbouring a cell with signal significance passing the threshold given by *N* in Eq. (4) is collected into a proto-cluster, independent of its signal. Using the correlations between energies in adjacent cells in this way allows the retention of cells with signals that are close to the noise levels while preserving the noise suppression feature of the clustering algorithm.

The implicit noise suppression implemented by the topo-cluster algorithm discussed above leads to significant improvements in various aspects of the calorimeter performance, such as the energy and spatial resolutions in the presence of pile-up. Contributions from large negative and positive signal fluctuations introduced by pile-up can survive in a given event, though, and thus contribute to the sensitivity to pile-up observed in e.g. the jet response [42], in addition to the cell-level effects mentioned in Sect. 2.2.1. Examples of the effect of this noise suppression on the cells contributing to zero-bias events recorded with ATLAS in 2012 are shown in the cell signal-significance spectra in Fig. 5a–d for four different LAr calorimeters in ATLAS.

#### Treatment of negative cell signals

Negative cell signals in the ATLAS calorimeters are the result of fluctuations introduced predominantly by pile-up and, to a lesser extent, by electronic noise, as discussed in Sects. 2.2.1 and 2.2.2. The thresholds in Eqs. (3)–(5) are applied in terms of the absolute value of $\varsigma _{\text {cell}}^{\,{{\text {EM}}}}$. This means that not only large positive cell signals can seed a cluster, but also those with large negative signals. In addition, cells with negative signals can also contribute to the cluster growth control and are added to the envelope around the topo-cluster core.

The use of cells with $E_{\text {cell}}^{{{\text {EM}}}}< 0$ as topo-cluster seeds provides a diagnostic tool for the amount of noise in the overall calorimeter signal for a given event. At the fixed noise value given in Eq. (1) and used in Eq. (3), the luminosity-dependent actual noise in the event is reflected in the number of topo-clusters reconstructed with negative seeds. This number serves as an estimator mainly for out-of-time pile-up.

Topo-clusters with negative seeds often have a total energy $E_{\text {clus}}^{{{\text {EM}}}}< 0$ as well, especially when $|\varsigma _{\text {cell}}^{\,{{\text {EM}}}}| \gg P$. This is due to the dominance of the negative seed and the correlation between this seed signal and signals in the neighbouring cells, which likely also have $E_{\text {cell}}^{{{\text {EM}}}}< 0$. If a negative seed signal is generated by out-of-time pile-up, it is induced by a particle injected into the calorimeter more than $100\,{\text {ns}}$ before the event. Its residual signal trace is scaled by the negative undershoot of the shaping function shown in Fig. 3. This particle also injected significant energy in the neighbouring cells at the same time, due to its electromagnetic or hadronic shower, which leads to $E_{\text {cell}}^{{{\text {EM}}}}< 0$ in these cells at the time of the event. For the same reasons, topo-clusters from out-of-time pile-up seeded by $E_{\text {cell}}^{{{\text {EM}}}}>0$ often yield $E_{\text {clus}}^{{{\text {EM}}}}> 0$, because they are typically generated by particles injected in past bunch crossings closer in time (within $100\,{\text {ns}}$). The topo-clusters with $E_{\text {clus}}^{{{\text {EM}}}}< 0$ can be used to provide an average global cancellation of contributions of clusters seeded by positive fluctuations in out-of-time pile-up in full event observables including $E_{\text {T}}^{\text {miss}}$  [44].

Clustering cells with $E_{\text {cell}}^{{{\text {EM}}}}< 0$ in any topo-cluster, including those containing and seeded by large positive signals, improves noise suppression due to the local cancellation of random positive (upward) noise fluctuations by negative (downward) fluctuations within this cluster. Allowing only positive signals to contribute introduces a bias in the cluster signal, while the random cancellation partially suppresses this bias.

To reconstruct physics objects such as jets from topo-clusters, only those clusters with a net energy $E_{\text {clus}}^{{{\text {EM}}}}> 0$ are considered. The expectation is that clusters with net negative energy have no contribution to the signal of the reconstructed object, as there is no correlation of the corresponding downward fluctuation mainly induced by the energy flow in previous bunch crossings with the final state that is triggered and reconstructed.

#### Cluster splitting

The proto-clusters built as described in Sect. 3.1.1 can be too large to provide a good measurement of the energy flow from the particles generated in the recorded event. This is true because spatial signal structures inside those clusters are not explicitly taken into account in the formation. In particular, local signal maxima indicate the presence of two or more particles injecting energy into the calorimeter in close proximity.

To avoid biases in jet-finding and to support detailed jet substructure analysis as well as a high-quality $E_{\text {T}}^{\text {miss}}$ reconstruction, proto-clusters with two or more local maxima are split between the corresponding signal peaks in all three spatial dimensions. A local signal maximum is defined by $E_{\text {cell}}^{{{\text {EM}}}}> 500\,\text {MeV}$, in addition to the topological requirements for this cell to have at least four neighbours and that none of the neighbours has a larger signal. Also, the location of cells providing local maxima is restricted to cells in the EM sampling layers EMB2, EMB3, EME2 and EME3, and to FCAL0. This means that for a proto-cluster located completely inside the electromagnetic calorimeters, or extending from the electromagnetic to the hadronic calorimeters, splitting is guided by the spatial cell signal distributions in the highly granular electromagnetic calorimeters. The cluster splitting is refined in an additional step, where signal maxima can be provided by cells from the thin EM sampling layers EMB1 and EME1 with a highly granular $\eta$-strip read-out geometry, all sampling layers in the hadronic calorimeters (HEC0 to HEC3, Tile0 to Tile2), and the hadronic forward calorimeter modules FCAL1 and FCAL2.^5^ The use of EMB1 and EME1 in the topo-cluster splitting improves the photon separation in $\pi ^{0}\rightarrow \gamma \gamma$.

**Fig. 6:**
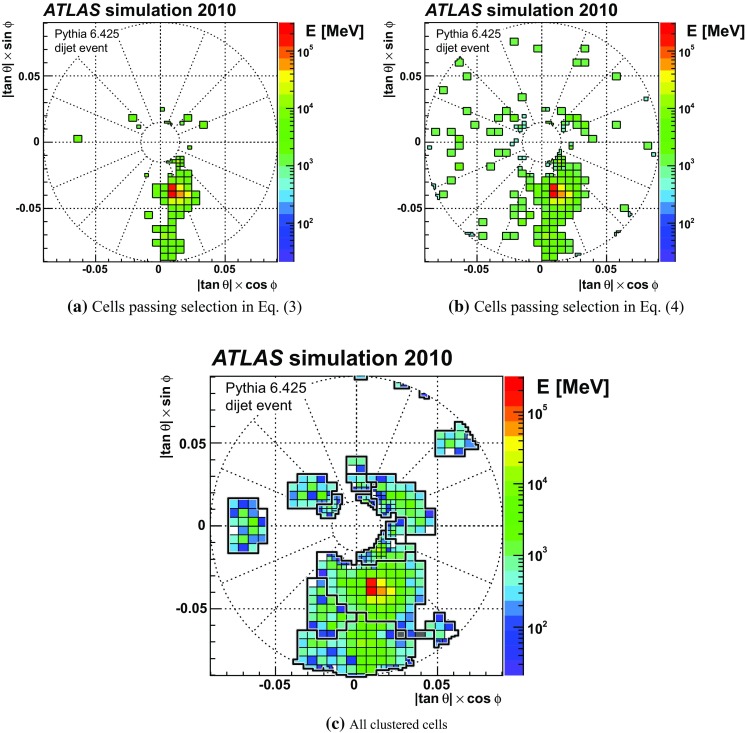
Stages of topo-cluster formation in the first module (FCAL0) of the FCAL calorimeter for a simulated dijet event with at least one jet entering this calorimeter. Shown in **a** are cells with signal significance $|\varsigma _{\text {cell}}^{\,{{\text {EM}}}}| > 4$ that can seed topo-clusters, in **b** cells with $|\varsigma _{\text {cell}}^{\,{{\text {EM}}}}| > 2$ controlling the topo-cluster growth, and in **c** all clustered cells and the outline of topo-clusters and topo-cluster fragments in this module. All clusters shown in **c** which do not contain a seed cell from this module are seeded in other modules of the FCAL, or in other calorimeters surrounding it. Pile-up is not included in this simulation, but electronic noise is modelled. Cells not *colour coded* but inside a topo-cluster have a negative signal, while cells *shaded grey* are completely surrounded by clustered cells but not part of a topo-cluster themselves. The cell and cluster boundaries are displayed on a dimensionless grid using the polar angle $\theta$ and the azimuthal angle $\phi$. This view maintains the cell shapes and proportions. For the definition of the cell signal significance $\varsigma _{\text {cell}}^{\,{{\text {EM}}}}$ see Eq. (2)

The cluster splitting algorithm can find cells which are neighbours to two or more signal maxima. In this case, the cell is assigned to the two highest-energy clusters after splitting of the original topo-cluster it is associated with. This means that each cell is only shared once at most, and, even then, is never shared between more than two clusters.

The sharing of its signal between the two clusters with respective energies $E_{\text {clus},1}^{{{\text {EM}}}}$ and $E_{\text {clus},2}^{{{\text {EM}}}}$ is expressed in terms of two geometrical weights $w_{\text {cell},1}^{\text {geo}}$ and $w_{\text {cell},2}^{\text {geo}}$. These weights are calculated from the distances of the cell to the centres of gravity of the two clusters ($d_{1}$, $d_{2}$), measured in units of a typical electromagnetic shower size scale in the ATLAS calorimeters,^6^ and the cluster energies,

6 $$\begin{aligned} w_{\text {cell},1}^{\text {geo}}= & {} \dfrac{E_{\text {clus},1}^{{{\text {EM}}}}}{E_{\text {clus},1}^{{{\text {EM}}}} + r E_{\text {clus},2}^{{{\text {EM}}}}}, \end{aligned}$$

7 $$\begin{aligned} w_{\text {cell},2}^{\text {geo}}= & {} 1 - w_{\text {cell},1}^{\text {geo}}, \end{aligned}$$

8 $$\begin{aligned} r= & {} \exp (d_{1}-d_{2}). \end{aligned}$$

The geometrical weights reflect the splitting rule that each cell can only appear in two proto-clusters at most, as $w_{\text {cell},1}^{\text {geo}} + w_{\text {cell},2}^{\text {geo}} = 1$. After splitting, the final proto-clusters are the topo-clusters used for further reconstruction of the recorded or simulated final state.

Figure 6 shows an example of topo-clusters generated by an MC simulated jet in the first module of the ATLAS forward calorimeter under 2010 run conditions (no pile-up). Possible seed cells, as defined in Eq. (3), are shown in Fig. 6a. Cells with signal significances above the threshold *N* specified in Eq. (4) are displayed in Fig. 6b. The cells from this module included in any topo-cluster are shown in Fig. 6c. This display shows the effectiveness of cluster splitting in tracing signal structures. Comparing Figs. 6a and c clearly shows the survival of cells with $|\varsigma _{\text {cell}}^{\,{{\text {EM}}}}| < 2$ in the vicinity of more significant signals, even if those are not in the same module (or sampling layer).

**Fig. 7:**
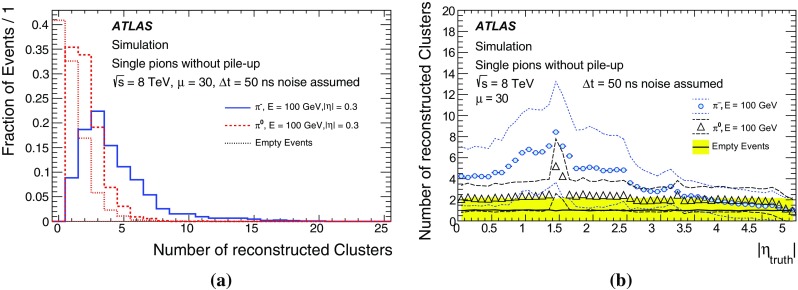
The number of reconstructed clusters for simulated charged and neutral single pions without actual pile-up added but with nominal pile-up noise used in the reconstruction. In **a** the distribution of the number of clusters $N_{\text {clus}}$ is shown for neutral and charged pions injected into the ATLAS calorimeters at $|\eta | = 0.3$ with an energy of $E = 100\,{\text {GeV}}$, together with the $N_{\text {clus}}$ distribution for empty events (topo-clusters generated by electronic noise only). The distributions are individually normalised to unity. The dependence of the average $\langle N_{\text {clus}}\rangle$ on the generated $\eta _{\text {gen}}$ is shown in **b** again for $\pi ^{0}$, $\pi ^{-}$ and empty events. The *shaded area* and the *dashed lines* indicate the spread (in terms of RMS) around the central value

#### Cluster multiplicities in electromagnetic and hadronic showers

One of the original motivations behind any cell clustering is to reconstruct single-particle showers with the highest possible precision in terms of energy and shape. The immediate expectation is that the clustering algorithm should be very efficient in reconstructing one cluster for each particle entering the calorimeter. While this view is appropriate for dense and highly compact electromagnetic showers with relatively small shower-to-shower fluctuations in their longitudinal (along the direction of flight of the incoming particle) and lateral (perpendicular to the direction of flight) extensions, hadronic showers are subject to much larger intrinsic fluctuations leading to large shower-to-shower variations in their shapes and compactness. Hadrons generated in inelastic interactions in the course of the hadronic shower can even travel significant distances and generate sub-showers outside the direct neighbourhood of the calorimeter cell containing the initial hadronic interaction. This means that topo-clusters can contain only a fraction of the hadronic shower.

The distributions of the topo-cluster multiplicity $N_{\text {clus}}$ for single particles which primarily generate electromagnetic showers ($\pi ^{0}$) and hadronic showers ($\pi ^{-}$) in the central (barrel) calorimeter region are shown in Fig. 7a. The dependence of the average $N_{\text {clus}}$ on the pseudorapidity $\eta$ is displayed in Fig. 7b.

Neutral pions with $E_{\pi ^{0}} = 100\,{\text {GeV}}$ injected into the detector at a fixed direction often generate only one topo-cluster from largely overlapping electromagnetic showers, as the angular distance between the two photons from $\pi ^{0}\!\rightarrow \!\gamma \gamma$ is small. This is demonstrated by the $N_{\text {clus}}$ distribution for topo-clusters generated by $\pi ^{0}$ at $|\eta | = 0.3$ in ATLAS in Fig. 7a peaking at $N_{\text {clus}}= 1$, with a probability only slightly larger than the one for $N_{\text {clus}}=2$. In the latter case the two topo-clusters from the $\pi ^{0}$ are generated by (1) resolving the two photon-induced showers, (2) a possible residual imperfect signal collection and proto-cluster splitting in the topo-cluster algorithm, or by (3) accidental inclusion of additional topo-cluster(s) generated by electronic noise. While the particular reason for the second cluster depends on effects introduced by local features including the calorimeter read-out granularity and cell noise levels at a given direction $\eta$, hypothesis (1) is found to be least likely as it is observed that the energy sharing between the two topo-clusters is typically very asymmetric. The leading topo-cluster generated by $\pi ^{0}$ at $100\,{\text {GeV}}$ contains very close to $100\,\%$ of the total energy in this calorimeter region, indicating that the second and any further topo-clusters arise from hypotheses (2) and (3).

Figure 7b shows the average $N_{\text {clus}}$ as a function of the generated particle direction $\eta = \eta _{\text {gen}}$. Especially around transition regions at $|\eta | \approx 1.4$ (central to end-cap calorimeters) and $|\eta | \approx 3.2$ (end-cap to forward calorimeters), which both have reduced calorimetric coverage, $N_{\text {clus}}$ can significantly increase due to reduction or loss of the core signal of the showers.

The number of clusters generated by $\pi ^{-}$ with $E = 100\,{\text {GeV}}$ injected at $\eta = 0.3$ peaks at $N_{\text {clus}}= 3$ and has a more significant tail to higher multiplicities, as shown in Fig. 7a. This is expected for hadronic showers, where the distance between two inelastic interactions with significant energy release is of the order of the nuclear interaction length $\lambda _{\text {nucl}}$, typically $\mathcal {O}(10\,{\text {cm}})$. This can lead to several well-separated topo-clusters. For example, at $100\,{\text {GeV}}$ incident energy the leading topo-cluster generated by $\pi ^{-}$ contains on average $85\,{\text {GeV}}$, while the next-to-leading topo-cluster contains about $10\,{\text {GeV}}$ on average. The remaining energy is distributed among one or more low-energy topo-clusters.

The wider hadronic shower spread introduces a higher sensitivity of $N_{\text {clus}}$ to the calorimeter read-out granularities and transition regions, as can be seen in Fig. 7b. The transition regions at $|\eta | \approx 0.8\text {--}1.0$, $|\eta | \approx 1.4$ and $|\eta | \approx 3.2$ affect the topo-cluster formation more than in the case of electromagnetic showers, not only in terms of the peak $N_{\text {clus}}$ but also in terms of the range in $\eta$. In particular the region around $|\eta | \approx 0.8\text {--}1.0$ has a larger effect on $N_{\text {clus}}$ for hadrons than for electromagnetic interacting particles, as this is the transition from the central to the extended Tile calorimeter introducing reduced calorimetric coverage for hadrons. The central electromagnetic calorimeter provides hermetic coverage here, without any effect on $N_{\text {clus}}$. The sharp drop of $N_{\text {clus}}$ for $\pi ^{-}$ at $|\eta | = 2.5$ corresponds to the reduction in calorimeter cell granularity by a factor of approximately four.

### Cluster kinematics

The cluster kinematics are the result of the recombination of cell energies and directions. The presence of cells with $E_{\text {cell}}^{{{\text {EM}}}}< 0$ requires a special recombination scheme to avoid directional biases.

The cluster directions are calculated as signal-weighted barycentres $(\eta _{\text {clus}},\phi _{\text {clus}})$. Using $E_{\text {cell}}^{{{\text {EM}}}}< 0$ in this scheme leads to distortion of these directions, even projecting them into the wrong hemispheres. Ignoring the contribution of cells with negative signals, on the other hand, biases the cluster directions with contributions from upward noise fluctuations. To avoid both effects, the cluster directions are calculated with absolute signal weights $|E_{\text {cell}}^{{{\text {EM}}}}|$,

9 $$\begin{aligned} \eta _{\text {clus}}= & {} \dfrac{\sum _{i=1}^{N_{\text {cell}}} w_{\text {cell},i}^{\text {geo}} \cdot |E_{\text {cell},i}^{\text {EM}}|\cdot \eta _{\text {cell},i}}{\sum _{i=1}^{N_{\text {cell}}} w_{\text {cell},i}^{\text {geo}} \cdot |E_{\text {cell},i}^{\text {EM}}|} \end{aligned}$$

10 $$\begin{aligned} \phi _{\text {clus}}= & {} \dfrac{\sum _{i=1}^{N_{\text {cell}}} w_{\text {cell},i}^{\text {geo}} \cdot |E_{\text {cell},i}^{\text {EM}}|\cdot \phi _{\text {cell},i}}{\sum _{i=1}^{N_{\text {cell}}} w_{\text {cell},i}^{\text {geo}} \cdot |E_{\text {cell},i}^{\text {EM}}|} . \end{aligned}$$

Here $N_{\text {cell}}$ is the number of cells in the cluster, and $w_{\text {cell},i}^{\text {geo}}$ are the geometrical signal weights introduced by cluster splitting, as given in Eqs. (6)–(8) in Sect. 3.1.3. The direction of each cell is given by $(\eta _{\text {cell}},\phi _{\text {cell}})$, calculated from its location with respect to the centre of ATLAS at $(x=0,y=0,z=0)$ in the detector reference frame. The cluster directions are therefore reconstructed with respect to this nominal detector centre.

The total cluster signal amplitude $E_{\text {clus}}^{{{\text {EM}}}}$ reflects the correct signal contributions from all cells,

11 $$\begin{aligned} E_{\text {clus}}^{{{\text {EM}}}}= \sum _{i=1}^{N_{\text {cell}}} w_{\text {cell},i}^{\text {geo}} E_{\text {cell},i}^{\text {EM}}, \end{aligned}$$

and is calculated using the signed cell signals $E_{\text {cell},i}^{\text {EM}}$ and taking into account the geometrical signal weights. In general, all clusters with $E_{\text {clus}}^{{{\text {EM}}}}> 0$ are used for the reconstruction of physics objects in the ATLAS calorimeters, including the very few ones seeded by cell signals $E_{\text {cell}}^{{{\text {EM}}}}<0$.

Each topo-cluster is interpreted as a massless *pseudo-particle* in physics object reconstruction. The energy and momentum components on the EM scale are calculated from the basic reconstructed kinematic variables $(E_{\text {clus}}^{{{\text {EM}}}},\eta _{\text {clus}},\phi _{\text {clus}})$ as

12 $$\begin{aligned} \text {P}_{\text {clus}}^{\,{{\text {EM}}}}= E_{\text {clus}}^{{{\text {EM}}}}\cdot \left( 1,\sin \theta _{\text {clus}}\cos \phi _{\text {clus}},\sin \theta _{\text {clus}}\sin \phi _{\text {clus}},\cos \theta _{\text {clus}}\right) = \left( E_{\text {clus}}^{{{\text {EM}}}},\vec {p}_{\text {clus}}^{\,\text {EM}}\right) \end{aligned}$$

with terms involving $\theta _{\text {clus}}$, the polar angle calculated from $\eta _{\text {clus}}$, and $\phi _{\text {clus}}$.

The massless pseudo-particle interpretation is appropriate as there is no physically meaningful cluster mass without a specific and valid particle hypothesis for the origin of the signal. Such a hypothesis seems to be impossible to obtain from the calorimeter signals alone, especially for hadrons or hadronically decaying particles, where particle identification often requires a measurement of the charge. A topo-cluster mass could in principle be reconstructed from the cell signals and their spatial distribution, but this observable is dominated by lateral shower spreading, which does not represent a physically meaningful mass. It is also highly affected by the settings for the noise thresholds, which control the lateral and longitudinal spread of the cluster in a given pile-up environment (see Sect. 3.1.1).

In addition, hadronic showers tend to be split more often into two or more topo-clusters, as discussed in Sect. 3.1.4 for single particles. Also, it is very likely in the proton–proton collision environment at the LHC that a given topo-cluster contains signals from several particles, especially when located inside a jet, as a mix of electromagnetic and hadronic showers or shower fragments. These issues make a physical particle hypothesis very unlikely, and any cluster mass measurement would be very hard to interpret or validate in relation to a “real” particle.

## Topo-cluster moments

**Fig. 8:**
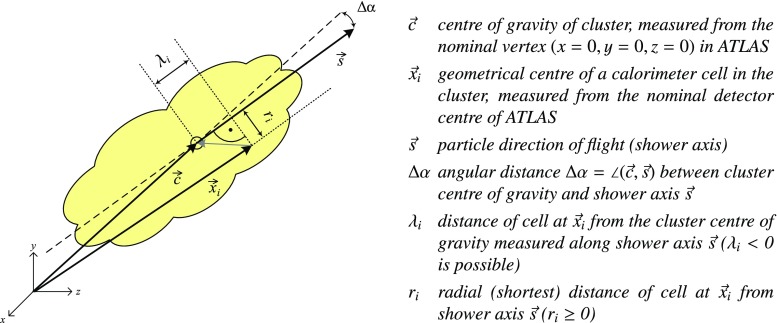
Schematic view of geometrical moments for topo-clusters

The shape of a topo-cluster and its internal signal distribution contain valuable information for signal characterisation with respect to its origin, and therefore cluster-based calibrations. The list of reconstructed observables (“cluster moments”) is long. In this section the focus is on moments used to evaluate the signal quality in data, to determine the cluster location and size, and to calibrate each cluster. The geometry relevant to some of the moments is depicted in Fig. 8. Moments which are useful for purely technical reasons, such as those related to the information about the true energy deposited in the calorimeter in MC simulations, are not discussed in this paper.

Most moments are defined at a given order *n* for a given calorimeter cell variable $\upsilon _{\text {cell}}$ as

13 $$\begin{aligned} \langle \upsilon _{\text {cell}}^{\,n} \rangle = \dfrac{\sum _{\{i\,|E_{\text {cell},i}^{\text {EM}}>0\}} w_{\text {cell},i}^{\text {geo}} E_{\text {cell},i}^{\text {EM}} \upsilon _{\text {cell},i}^{\,n}}{\sum _{\{i\,|E_{\text {cell},i}^{\text {EM}} > 0\}} w_{\text {cell},i}^{\text {geo}} E_{\text {cell},i}^{\text {EM}}} . \end{aligned}$$

All moments use the EM scale cell signals $E_{\text {cell}}^{{{\text {EM}}}}$, thus they do not depend on any refined calibration. The moment calculation is further restricted to in-time signals, meaning only cells with $E_{\text {cell}}^{{{\text {EM}}}}> 0$ are considered. Even though higher-order moments can be reconstructed, only centroids ($n = 1$) and spreads ($n = 2$) are used.

### Geometrical moments

Each topo-cluster with at least three cells with $E_{\text {cell}}^{{{\text {EM}}}}> 0$ has a full set of geometrical moments. Simple directional moments (barycentres in $(\eta ,\phi )$ space) and locations (centres of gravity) are available for all clusters. Not all geometrical moments can be evaluated in a meaningful way for all topo-clusters, mostly due to lack of relevant information in clusters with few cells. In this case, a default value specific to each of these moments is provided.

#### Location

The location of a topo-cluster is defined by its centre of gravity $\vec {c}$ in three-dimensional space, as shown in Fig. 8. This centre is calculated from the first moments of the three Cartesian coordinates specifying the calorimeter cell centres, following the definition given in Eq. (13). These locations are provided in the nominal detector frame of reference defined by the interaction point (IP) being located at $(x=0,y=0,z=0)$.

In addition to the absolute location measured by the centre of gravity, the distance $\lambda _{\text {clus}}$ of the centre of gravity from the calorimeter front face, determined along the shower axis (see below and Fig. 8), is calculated for each topo-cluster.

#### Directions

The direction of a topo-cluster is given by $$(\eta _{\text {clus}},\phi _{\text {clus}})$$, reconstructed as given in Eqs. (9) and (10). In addition, the first- and second-order directional moments using $$\eta _{\text {cell}}$$ and $$\phi _{\text {cell}}$$ are calculated using Eq. (13) with $$n =1$$ and $$n =2 $$, respectively.^7^ The reference for these direction measures is the IP discussed above.

The shower axis is a measure of the direction of flight of the incoming particle. It is defined by a principal value analysis of the energy-weighted spatial correlations between cells with $E_{\text {cell}}^{{{\text {EM}}}}> 0$ with respect to the cluster centre in Cartesian coordinates,

14 $$\begin{aligned} C_{uv} = \dfrac{1}{\mathcal {W}} \sum _{\{i\,|E_{\text {cell},i}^{\text {EM}} > 0\}} \left( w_{\text {cell},i}^{\text {geo}}E_{\text {cell},i}^{\text {EM}}\right) ^{2} (u_{i} - \langle u \rangle )(v_{i} - \langle v \rangle ) , \end{aligned}$$

with all permutations of $u,v \in \{x,y,z\}$. The normalisation $\mathcal {W}$ is given by

15 $$\begin{aligned} \mathcal {W} = \sum _{\{i\,|E_{\text {cell},i}^{\text {EM}} > 0\}} \left( w_{\text {cell},i}^{\text {geo}}E_{\text {cell},i}^{\text {EM}}\right) ^{2} . \end{aligned}$$

The $C_{uv}$ fill a symmetric $3 \times 3$ matrix $\mathbf{C} = \left[ C_{uv}\right]$. The eigenvector of $\mathbf{C}$ closest to the direction $\vec {c}$ from the IP to the centre of gravity of the topo-cluster is taken to be the shower axis $\vec {s}$. If the angular distance $\Delta \alpha$ between $\vec {c}$ and $\vec {s}$ is $\Delta \alpha > 20^{\circ }$, $\vec {c}$ is used as the shower axis. Figure 8 depicts the geometry of the two axis definitions for topo-clusters.

#### Extensions and sizes

The size of the topo-cluster is calculated with respect to the shower axis $\vec {s}$ and the centre of gravity $\vec {c}$. For this, cells are first located with reference to $\vec {s}$ and $\vec {c}$. The distances of a cell at $\vec {x}_{i}$ to the shower axis and the centre of gravity are then given by

16 $$\begin{aligned} r_{i}&= | (\vec {x}_{i} - \vec {c}\,) \times \vec {s}\,|&\text {(radial distance to shower axis)}; \end{aligned}$$

17 $$\begin{aligned} \lambda _{i}&= (\vec {x}_{i} - \vec {c}\,) \cdot \vec {s}&\text {(longitudinal distance from shower centre of gravity)}. \end{aligned}$$

The first moment $\langle \lambda \rangle$ calculated according to Eq. (13) with $\upsilon _{\text {cell},i} = \lambda _{i}$ and $n =1$ is $\langle \lambda \rangle = 0$ by definition. The same equation is used for the first moment $\langle r\rangle$ of $r_{i}$ ($\upsilon _{\text {cell},i} = r_{i}$, $n = 1$). The longitudinal and lateral extensions of a topo-cluster can then respectively be measured in terms of the second moments $\langle \lambda^{2}\rangle$ and $\langle r^{2}\rangle$, again using Eq. (13), but with $n = 2$. Specifying cluster dimensions in this fashion describes a spheroid with two semi-axes of respective lengths $\sqrt{\langle \lambda ^{2}\rangle }$ and $\sqrt{\langle r^{2}\rangle }$.

As calorimeter technologies and granularities change as function of $\eta$ in ATLAS, measures representing the lateral and longitudinal extension of topo-clusters in a more universal and normalised fashion are constructed. These measures are defined in terms of second moments with value ranges from 0 to 1,

18 $$\begin{aligned} \mathfrak {m}_{\text {lat}}^{2}&= \dfrac{\langle r^{2}\rangle _{\text {out}}}{\langle r^{2}\rangle _{\text {out}} + \langle r^{2}\rangle _{\text {core}}}&\text {normalised lateral energy dispersion (width measure)}; \end{aligned}$$

19 $$\begin{aligned} \mathfrak {m}_{\text {long}}^{2}&= \dfrac{\langle \lambda ^{2}\rangle _{\text {out}}}{\langle \lambda ^{2}\rangle _{\text {out}} + \langle \lambda ^{2}\rangle _{\text {core}}}&\text {normalised longitudinal energy dispersion (length measure)}. \end{aligned}$$

The $\langle r^{2}\rangle _{\text {out}}$ term is calculated using Eq. (13) with $n =2$ and $\upsilon _{\text {cell},i} = r_{i}$, but with $r_{i} = 0$ for the two most energetic cells in the cluster. The term $\langle r^{2}\rangle _{\text {core}}$ is calculated with the same equation, but now with a fixed $r_{i} = r_{\text {core}}$ for the two most energetic cells, and $r_{i} = 0$ for the rest. The calculation of the corresponding terms $\langle \lambda ^{2}\rangle _{\text {out}}$ and $\langle \lambda ^{2}\rangle _{\text {core}}$ for $\mathfrak {m}_{\text {long}}^{2}$ follows the same respective rules, now with $\upsilon _{\text {cell},i} = \lambda _{i}$ in Eq. (13) and $\lambda _{\text {core}}$ for the most energetic cells in $\langle \lambda ^{2}\rangle _{\text {core}}$.^8^

The normalised moments $\mathfrak {m}_{\text {long}}^{2}$ and $\mathfrak {m}_{\text {lat}}^{2}$ do not directly provide a measure of spatial topo-cluster dimensions, rather they measure the energy dispersion in the cells belonging to the topo-cluster along the two principal cluster axes. Characteristic values are $\mathfrak {m}_{\text {long}}^{2}\rightarrow 0$ ($\mathfrak {m}_{\text {lat}}^{2}\rightarrow 0$) indicating few highly energetic cells distributed in close proximity along the longitudinal (lateral) cluster extension, and $\mathfrak {m}_{\text {long}}^{2}\rightarrow 1$ ($\mathfrak {m}_{\text {lat}}^{2}\rightarrow 1$) indicating a longitudinal (lateral) distribution of cells with more similar energies. Small values of $\mathfrak {m}_{\text {long}}^{2}$ ($\mathfrak {m}_{\text {lat}}^{2}$) therefore mean short (narrow) topo-clusters, while larger values are indicative of long (wide) clusters.

The effective size of the topo-cluster in $(\eta ,\phi )$ space can in good approximation be estimated as^9^

20 $$\begin{aligned} \sigma _{\eta } \simeq \sigma _{\phi } \simeq \quad {{\mathrm{atan}}}\quad \left( \dfrac{\sqrt{\langle r^{2}\rangle }}{|\vec {c}\,|}\right) \times \cosh (\eta ). \end{aligned}$$

The fact that this approximation holds for both the cluster size in $\eta$ ($\sigma _{\eta }$) and $\phi$ ($\sigma _{\phi }$) is due to the particular granularity of the ATLAS calorimeters.

### Signal moments

Topo-cluster moments related to the distribution of the cell signals inside the cluster are useful in determining the density and compactness of the underlying shower, the significance of the cluster signal itself, and the quality of the cluster reconstruction. These moments thus not only provide an important input to the calibrations and corrections discussed in Sect. 5, but also support data quality driven selections in the reconstruction of physics objects. Additional topo-cluster signal quality moments related to instantaneous, short term, and long term detector defects introducing signal efficiency losses are available but very technical in nature, and very specific to the ATLAS calorimeters. Their discussion is outside of the scope of this paper.

#### Signal significance

The significance of the topo-cluster signal is an important measure of the relevance of a given cluster contribution to the reconstruction of physics objects. Similar to the cell signal significance $\varsigma _{\text {cell}}^{\,{{\text {EM}}}}$ given in Eq. (2) in Sect. 3.1, it is measured with respect to the total noise $\sigma _{\text {noise,clus}}^{\,{{\text {EM}}}}$ in the topo-cluster. The definition of $\sigma _{\text {noise,clus}}^{\,{{\text {EM}}}}$ assumes incoherent noise in the cells contributing to the topo-cluster,^10^

21 $$\begin{aligned} \sigma _{\text {noise,clus}}^{\,{{\text {EM}}}}= \sqrt{\sum _{i=1}^{N_{\text {cell}}} \left( \sigma _{\text {noise,cell},i}^{\,{{\text {EM}}}}\right) ^{2}}. \end{aligned}$$

Here $N_{\text {cell}}$ is the number of cells forming the cluster, including the ones with $E_{\text {cell}}^{{{\text {EM}}}}< 0$. As discussed in Sect. 2.2.2, the individual overall cell noise $\sigma _{\text {noise,cell},i}^{\,{{\text {EM}}}}$ is set according to the nominal pile-up condition for a given data taking period. The topo-cluster signal significance $\varsigma _{\text {clus}}^{\,\text {EM}}$ is then measured using $\sigma _{\text {noise,clus}}^{\,{{\text {EM}}}}$ and $E_{\text {clus}}^{{{\text {EM}}}}$,

22 $$\begin{aligned} \varsigma _{\text {clus}}^{\,\text {EM}}= \dfrac{E_{\text {clus}}^{{{\text {EM}}}}}{\sigma _{\text {noise,clus}}^{\,{{\text {EM}}}}} . \end{aligned}$$

In addition to $\varsigma _{\text {clus}}^{\,\text {EM}}$, $\varsigma _{\text {cell}}^{\,{{\text {EM}}}}$ of the cell with the highest significant signal (the original cluster seed) is available to further evaluate the topo-cluster. A highly significant seed is a strong indication of an important cluster signal, even if $\varsigma _{\text {clus}}^{\,\text {EM}}$ may be reduced by inclusion of a larger number of less significant cell signals.

#### Signal density

The signal density of the topo-cluster is indicative of the nature of the underlying particle shower. It can be evaluated in two different approaches. First, $E_{\text {clus}}^{{{\text {EM}}}}$ can be divided by the volume the cluster occupies in the calorimeter. This volume is the sum of volumes of all cells contributing to the cluster. The signal density reconstructed this way is subject to considerable instabilities introduced by signal fluctuations from noise, as large volume cells can be added with a very small signal due to those fluctuations.

The default for topo-cluster calibration is the second and more stable estimate of the topo-cluster signal density measured by the cell-energy-weighted first moment $\rho _{\text {clus}}= \langle \rho _{\text {cell}}\rangle$ of the signal densities $\rho _{\text {cell},i} = E_{\text {cell},i}^{\text {EM}}/V_{\text {cell},i}$ of cells $i = 1 \ldots N_{\text {cell}}$ forming the cluster. Here $V_{\text {cell},i}$ is the volume of cell *i*. The $\rho _{\text {clus}}$ variable is calculated using Eq. (13) with $\upsilon _{\text {cell},i} = \rho _{\text {cell},i}$ and $n =1$. It is much less sensitive to the accidental inclusion of large volume cells with small signals into the cluster, and is used in the context of topo-cluster calibration. The corresponding second moment is calculated using Eq. (13) with $n = 2$. It indicates the spread of cell energy densities in the topo-cluster, thus its compactness.

#### Signal timing

The topo-cluster signal timing is a sensitive estimator of its signal quality. It is particularly affected by large signal remnants from previous bunch crossings contributing to the cluster, or even exclusively forming it, and can thus be employed as a tag for topo-clusters indicating pile-up activity.

The reconstructed signal $E_{\text {cell}}^{{{\text {EM}}}}$ in all calorimeter cells in ATLAS is derived from the reconstruction of the peak amplitude of the time-sampled analogue signal from the calorimeter shaping amplifiers. In the course of this reconstruction the signal peaking time $t_{\text {cell}}$ with respect to the $40\,\text {MHz}$ LHC bunch crossing clock is determined as well. The timing $t_{\text {clus}}$ of a topo-cluster is then calculated from $t_{\text {cell},i}$ of the clustered cells $i = 1\ldots N_{\text {cell}}$ according to

23 $$\begin{aligned} t_{\text {clus}}= \dfrac{\sum _{\{i|\varsigma _{\text {cell},i}^{{{\text {EM}}}}>2\}} \left( w_{\text {cell},i}^{\text {geo}}E_{\text {cell},i}^{\text {EM}}\right) ^{2} t_{\text {cell},i}}{\sum _{\{i|\varsigma _{\text {cell},i}^{{{\text {EM}}}}>2\}} \left( w_{\text {cell},i}^{\text {geo}}E_{\text {cell},i}^{\text {EM}}\right) ^{2}}, \end{aligned}$$

where only cells with a signal significance $\varsigma _{\text {cell},i}^{{{\text {EM}}}}$ sufficient to reconstruct $E_{\text {cell},i}^{\text {EM}}$ and $t_{\text {cell},i}$ are used ($\varsigma _{\text {cell},i}^{{{\text {EM}}}} > 2$). The particular weight of the contribution of $t_{\text {cell},i}$ to $t_{\text {clus}}$ in Eq. (23) is found to optimise the cluster timing resolution [6].

#### Signal composition

The signal distribution inside a topo-cluster is measured in terms of the energy sharing between the calorimeters contributing cells to the cluster, and other variables measuring the cell signal sharing. The energy sharing between the electromagnetic and hadronic calorimeters is expressed in terms of the signal ratio $f_{\text {emc}}$, and can be used as one of the characteristic observables indicating an underlying electromagnetic shower. The signal fraction $f_{\text {max}}$ carried by the most energetic cell in the cluster is a measure of its compactness. The signal fraction $f_{\text {core}}$ of the summed signals from the highest energetic cell in each longitudinal calorimeter sampling layer contributing to the topo-cluster can be considered as a measure of its *core signal strength*. It is sensitive not only to the shower nature but also to specific features of individual hadronic showers. These fractions are calculated for each topo-cluster with $E_{\text {clus}}^{{{\text {EM}}}}> 0$ as follows (EMC denotes the electromagnetic calorimeters^11^ in ATLAS),

24 $$\begin{aligned} f_{\text {emc}}&= \dfrac{1}{E_{\text {clus},\text {pos}}^{{{\text {EM}}}}}\sum _{\{i\,\in \,\mathrm{EMC};E_{\text {cell},i}^{\text {EM}} > 0\}} w_{\text {cell},i}^{\text {geo}}E_{\text {cell},i}^{\text {EM}}&(\mathrm{EMC{} signal fraction in cluster}); \end{aligned}$$

25 $$\begin{aligned} f_{\text {max}}&= \dfrac{1}{E_{\text {clus},\text {pos}}^{{{\text {EM}}}}}\max \left\{ w_{\text {cell},i}^{\text {geo}}E_{\text {cell},i}^{\text {EM}}\right\}&(\text {most energetic cell signal fraction in cluster}); \end{aligned}$$

26 $$\begin{aligned} f_{\text {core}}&= \dfrac{1}{E_{\text {clus},\text {pos}}^{{{\text {EM}}}}}\sum _{s\,\in \,\{\text {samplings}\}} \max _{i\,\in \,s}\left\{ w_{\text {cell},i}^{\text {geo}}E_{\text {cell},i}^{\text {EM}}\right\}&(\text {core signal fraction in cluster}). \end{aligned}$$

The index *s* steps through the set of calorimeter sampling layers with cells contributing to the topo-cluster. Only cells with $E_{\text {cell}}^{{{\text {EM}}}}> 0$ are used in the calculation of these fractions. Correspondingly, they are normalised to $E_{\text {clus},\text {pos}}^{{{\text {EM}}}}$ given by

27 $$\begin{aligned} E_{\text {clus},\text {pos}}^{{{\text {EM}}}} = \sum _{\{i|E_{\text {cell},i}^{\text {EM}}>0\}} w_{\text {cell},i}^{\text {geo}}E_{\text {cell},i}^{\text {EM}}. \end{aligned}$$

All these moments have a value range of [0,1].

One of the variables that can be considered for further evaluation of the relevance of the cluster signal in the presence of pile-up is the ratio of $E_{\text {clus},\text {pos}}^{{{\text {EM}}}}$ to $E_{\text {clus}}^{{{\text {EM}}}}$. It is sensitive to the negative energy content of a given topo-cluster which is largely injected by out-of-time pile-up dominated by the negative tail of the bipolar signal shaping function discussed in Sect. 3.1.2.

#### Topological isolation

The implicit noise suppression in the topological clustering algorithms leads to signal losses affecting the calorimeter response to particles, as further discussed in Sect. 5.4. As these signal losses appear at the boundary of the topo-cluster, corresponding corrections need to be sensitive to whether the lost signals may be included in another close-by cluster or if they are lost for good. This is particularly important for jets, where the topo-cluster density can be very high.

The degree of isolation is measured by the isolation moment $f_{\text {iso}}$, with $0 \le f_{\text {iso}} \le 1$. A topo-cluster with $f_{\text {iso}} = 1$ is completely isolated, while a cluster with $f_{\text {iso}} = 0$ is completely surrounded by others. The isolation measures the sampling layer energy ($E_{s}^{{{\text {EM}}}}$)-weighted fraction of non-clustered neighbour cells on the outer perimeter of the topo-cluster. Here $E_{s}^{{{\text {EM}}}}$ is defined as the sum of the energies $E_{\text {cell}}^{{{\text {EM}}}}$ of all cells in a topo-cluster located in a given sampling layer *s* of the calorimeter.

The isolation moment is reconstructed by first counting the number of calorimeter cells $N_{\text {cell},s}^{\text {noclus}}$ in sampling layer *s* neighbouring a topo-cluster but not collected into one themselves. Second, the ratio $N_{\text {cell},s}^{\text {noclus}}/N_{\text {cell},s}^{\text {neighbour}}$ of this number to the number of all neighbouring cells ($N_{\text {cell},s}^{\text {neighbour}}$) for each *s* contributing to the cluster is calculated. The per-cluster $E_{s}^{{{\text {EM}}}}$-weighted average of these ratios from all included *s* is the isolation moment $f_{\text {iso}}$,

28 $$\begin{aligned} f_{\text {iso}} = \dfrac{\sum _{s \in \{\text {samplings with }E_{s}^{{{\text {EM}}}}>0\}} E_{s}^{{{\text {EM}}}} N_{\text {cell},s}^{\text {noclus}}/N_{\text {cell},s}^{\text {neighbour}}}{\sum _{s \in \{\text {samplings with }E_{s}^{{{\text {EM}}}}>0\}} E_{s}^{{{\text {EM}}}}}. \end{aligned}$$

## Local hadronic calibration and signal corrections

The motivation for the calibration scheme described in this section arises from the intention to provide a calorimeter signal for physics object reconstruction in ATLAS which is calibrated outside any particular assumption about the kind of object. This is of particular importance for final-state objects with a significant hadronic signal content, such as jets and, to a lesser degree, $\tau$-leptons. In addition to these discrete objects, the precise reconstruction of the missing transverse momentum requires well-calibrated hadronic signals even outside hard final-state objects, to e.g. avoid deterioration of the $E_{\text {T}}^{\text {miss}}$ resolution due to highly fluctuating (fake) $p_{\text {T}}$-imbalances introduced by the non-linear hadronic response on the EM scale.

The topo-cluster moments provide information sensitive to the nature of the shower generating the cluster signal. This information can be explored to apply moment-dependent calibrations cluster-by-cluster, and thus correct for the effects of the non-compensating calorimeter response to hadrons, accidental signal losses due to the clustering strategy, and energy lost in inactive material in the vicinity of the topo-cluster. The calibration strategy discussed in some detail in the following is *local* because it attempts to calibrate highly localised and relatively small (in transverse momentum flow space) topo-clusters.^12^ As the local hadronic calibration includes cell signal weighting, the calibration based on topo-clusters is referred to as “local hadronic cell weighting” (LCW) calibration.

All calibrations and corrections are derived using MC simulations of single pions (charged and neutral) at various energies in all ATLAS calorimeter regions. This fully simulation-based approach requires good agreement between data and these MC simulations for the topo-cluster signals and moments used for any of the applied corrections in terms of distribution shapes and averages. Reconstructed observables which are not well-modelled by simulation are not considered. The data/MC comparisons for most used observables are shown in the context of the discussion of the methods using them.

### General topo-cluster calibration strategy

The LCW calibration aims at the cluster-by-cluster reconstruction of the calorimeter signal on the appropriate (electromagnetic or hadronic) energy scale. In this, the cluster energy resolution is expected to improve by using other information in addition to the cluster signal in the calibration. The basic calorimeter signal inefficiencies that this calibration must address are given below.

- **Non-compensating calorimeter response:** All calorimeters employed in ATLAS are non-compensating, meaning their signal for hadrons is smaller than the one for electrons and photons depositing the same energy ($e/\pi >1$). Applying corrections to the signal locally so that $e/\pi$ approaches unity on average improves the linearity of the response as well as the resolution for jets built from a mix of electromagnetic and hadronic signals. It also improves the reconstruction of full event observables such as $E_{\text {T}}^{\text {miss}}$, which combines signals from the whole calorimeter system and requires balanced electromagnetic and hadronic responses in and outside signals from (hard) particles and jets.
- **Signal losses due to clustering:** The topo-cluster formation applies an intrinsic noise suppression, as discussed in detail in Sect. 3.1. Depending on the pile-up conditions and the corresponding noise thresholds, a significant amount of true signal can be lost this way, in particular at the margins of the topo-cluster. This requires corrections to allow for a more uniform and linear calorimeter response.
- **Signal losses due to energy lost in inactive material:** This correction is needed to address the limitations in the signal acceptance in active calorimeter regions due to energy losses in nearby inactive material in front, between, and inside the calorimeter modules.

**Fig. 9:**
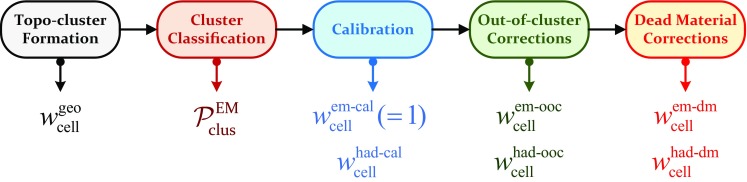
Overview of the local hadronic cell-weighting (LCW) calibration scheme for topo-clusters. Following the topo-cluster formation, the likelihood for a cluster to be generated by electromagnetic energy deposit ($\mathcal {P}_{\text {clus}}^{\,{{\text {EM}}}}$) is calculated. After this, the sequence of calibration and corrections indicated in the schematics is executed, each yielding cell signal weights for the two possible interpretations of the cluster signals. These weights are indicated in the figure. They are then used together with $\mathcal {P}_{\text {clus}}^{\,{{\text {EM}}}}$ to calculate the topo-cluster energy and barycentre from the contributing calorimeter cells, as described in the text

The corrections collected in the LCW calibration address these three main sources of signal inefficiency. The specifics of the calibrations and corrections applied to correct for these signal inefficiencies depend on the nature of the energy deposit – hadronic (HAD) or electromagnetic (EM). Therefore, the first step of the topo-cluster calibration procedure is to determine the probability $0 \le \mathcal {P}_{\text {clus}}^{\,{{\text {EM}}}}\le 1$ that a given topo-cluster is generated by an electromagnetic shower. This approach provides straightforward dynamic scales (cluster-by-cluster) for the application of specific electromagnetic ($\mathcal {P}_{\text {clus}}^{\,{{\text {EM}}}}$) and hadronic ($1-\mathcal {P}_{\text {clus}}^{\,{{\text {EM}}}}$) calibrations and corrections. For topo-clusters with $\mathcal {P}_{\text {clus}}^{\,{{\text {EM}}}}= 1$, it suppresses the application of a hadronic calibration mostly addressing the non-compensating response to hadrons, and applies the electromagnetic-signal-specific corrections for the losses introduced by clustering and inactive material mentioned above. Reversely, very hadronic topo-clusters with $\mathcal {P}_{\text {clus}}^{\,{{\text {EM}}}}= 0$ receive the appropriate hadronic calibration and hadronic-signal-specific signal loss corrections.

The main differences in the hadronic and electromagnetic calibration of topo-clusters are the magnitudes of the applied corrections, which in the EM case are significantly smaller than for HAD. Applying an exclusive categorisation based on the probability distributions described in Sect. 5.2 can lead to inconsistent calibrations especially for low-energy or small (few cells only) clusters, as misclassification for these kinds of topo-clusters is more likely than for clusters with higher energies or larger sizes. To allow for smooth transitions and reduce the dependency on the classification, the signal weights $w_{\text {cell}}^{\text {cal}}$ applied to cell signals in the topo-cluster at any of the calibration and correction steps are calculated as

29 $$\begin{aligned} w_{\text {cell}}^{\text {cal}}= \mathcal {P}_{\text {clus}}^{\,{{\text {EM}}}}\cdot w_{\text {cell}}^{\text {em-cal}}+ ( 1 - \mathcal {P}_{\text {clus}}^{\,{{\text {EM}}}})\cdot w_{\text {cell}}^{\text {had-cal}}. \end{aligned}$$

The weights $w_{\text {cell}}^{\text {em-cal}}$ and $w_{\text {cell}}^{\text {had-cal}}$ represent the factors applied by the EM or HAD calibration to the cell signal. The effective representation of all calibration steps in terms of these cell-level signal weights implements a consistent approach independent of the nature of the actual correction applied at any given step. As detailed in Sects. 5.3–5.5, the weights can depend on the cell signal itself, thus yielding a different weight for each cell. They can also represent cluster-level corrections generating the same weight for all cells, or a subset of cells, of the topo-cluster. This cell weighting scheme therefore provides not only the corrected overall cluster energy after each calibration step by weighted cell signal re-summation, but also the corresponding (possibly modified) cluster barycentre. Thus the cumulative effect on the topo-cluster energy and direction can be validated after each step. The steps of the general LCW calibration are schematically summarised in Fig. 9, and the individual steps are described in more detail below.

The EM calibrations and corrections and their respective parameters are determined with single-particle MC simulations of neutral pions for a large set of energies distributed uniformly in terms of $\log (E)$ between $200\,\text {MeV}$ and $2\,{\text {TeV}}$, at various directions $\eta$. The same energy and $\eta$ phase space is used for the corresponding simulations of charged pions to determine the HAD calibrations and corrections. The signals in these simulations are reconstructed with thresholds corresponding to the nominal $\sigma _{\text {noise}}^{\,{{\text {EM}}}}$ for a given run period, which reflects the pile-up conditions according to Eq. (1) in Sect. 2.2.2. Only electronic noise is added into the signal formation in the MC simulation, so that the derived calibrations and corrections effectively correct for signal losses introduced by the clustering itself. In particular, additional signal from pile-up and modifications of the true signal by out-of-time pile-up are not considered, as these are expected to cancel on average.

### Cluster classification

**Fig. 10:**
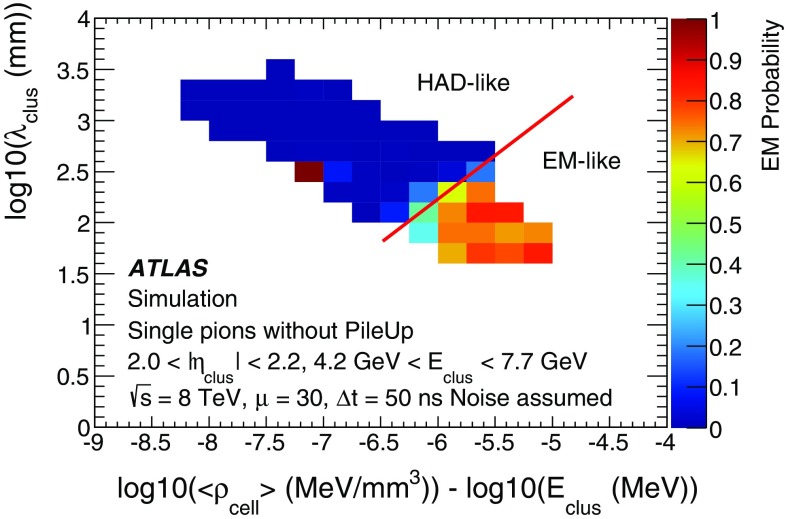
Distribution of the likelihood $\mathcal {P}_{\text {clus}}^{\,{{\text {EM}}}}(\rho _{\text {clus}}/E_{\text {clus}}^{{{\text {EM}}}},\lambda _{\text {clus}})$ for reconstructed topo-clusters to originate from an electromagnetic shower as a function of the shower depth $\lambda _{\text {clus}}$ and the normalised cluster signal density $\rho _{\text {clus}}/E_{\text {clus}}^{{{\text {EM}}}}$, with $\rho _{\text {clus}}= \langle \rho _{\text {cell}}\rangle$ being the energy-weighted average of $\rho _{\text {cell}}$. The shown distribution is determined as described in the text, in a selected bin of the cluster energy $E_{\text {clus}}^{{{\text {EM}}}}$ and the cluster direction $\eta _{\text {clus}}$. The *red line* indicates the boundary of the $\mathcal {P}_{\text {clus}}^{\,{{\text {EM}}}}> 50\,$% selection, below which the topo-cluster is classified as mostly electromagnetic (“EM-like”) and above which it is classified as mostly hadronic (“HAD-like”). The small EM-like area at the edge of the HAD-like region stems from neutral pions showering late. These areas are typical in regions of the detector where the second layer of the EM calorimeter is thinner and substantial parts of the shower are deposited in its last layer. The larger volume of the cells in this last layer leads to the reduced energy density while the position at the back of the EM calorimeter means a larger $\lambda _{\text {clus}}$

As discussed in Sect. 4, most topo-clusters provide geometrical and signal moments sensitive to the nature of the shower producing the cluster signal. In particular, electromagnetic showers with their compact shower development, early starting point and relatively small intrinsic fluctuations can generate cluster characteristics very different from those generated by hadronic showers. The latter are in general subjected to larger shower-by-shower fluctuations in their development and can be located deeper into the calorimeter. In addition, the hadronic showers show larger variations of their starting point in the calorimeter. A classification of each topo-cluster according to its likely origin determines the most appropriate mix of EM and HAD calibration and correction functions to be applied.

**Fig. 11:**
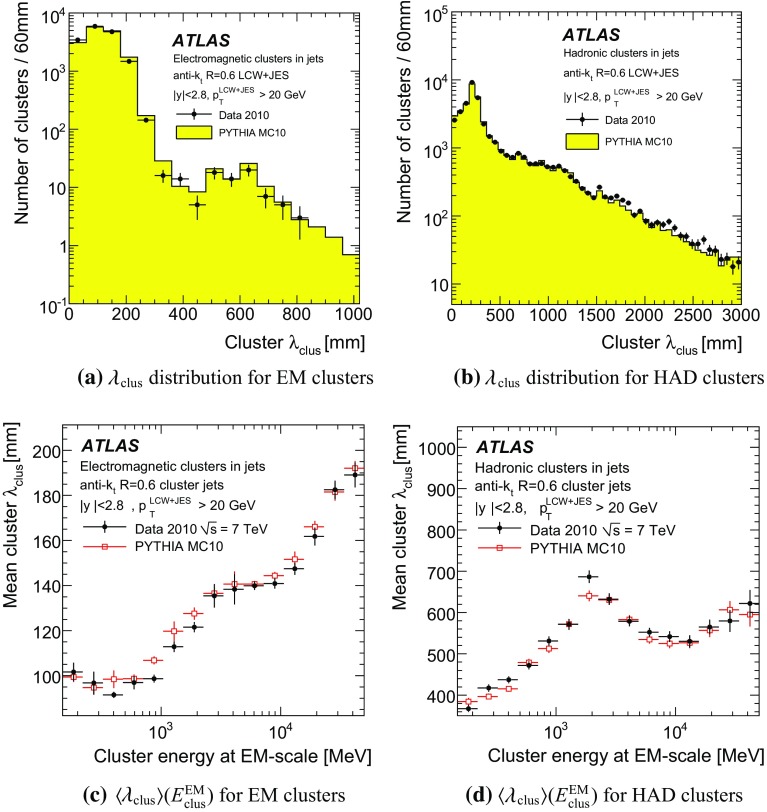
The distribution of the longitudinal depth $\lambda _{\text {clus}}$ of topo-cluster inside anti-$k_{t}$ jets with $R = 0.6$, $|y|<2.8$, and $p_{\text {T}}> 20$ GeV, for clusters classified as (**a**) electromagnetic (EM) and (**b**) hadronic (HAD), in 2010 data and MC simulations (no pile-up). Also shown is the average topo-cluster depth $\langle \lambda _{\text {clus}}\rangle$ as function of the cluster energy $E_{\text {clus}}^{{{\text {EM}}}}$ for the same topo-clusters classified as (**c**) EM and (**d**) HAD, respectively. The figures are adapted from Ref. [38]

The depth $\lambda _{\text {clus}}$ of the topo-cluster (Sect. 4.1.1) and its average cell signal density $\rho _{\text {clus}}$ (Sect. 4.2.2), both determined in bins of the cluster energy $E_{\text {clus}}^{{{\text {EM}}}}$ and the cluster direction $\eta _{\text {clus}}$, are found to be most efficient in classifying the topo-clusters. Using the MC simulations of single charged and neutral pions entering the calorimeters at various pseudorapidities and at various momenta, the probability for a cluster to be of electromagnetic origin ($\mathcal {P}_{\text {clus}}^{\,{{\text {EM}}}}$) is then determined by measuring the efficiency for detecting an EM-like cluster in bins of four topo-cluster observables,

30 $$\begin{aligned} \mathfrak {O}_{\text {clus}}^{\text {class}} = \left\{ \;E_{\text {clus}}^{{{\text {EM}}}},\;\eta _{\text {clus}},\;\log _{10}(\rho _{\text {clus}}/\rho _{0})-\log _{10}(E_{\text {clus}}^{{{\text {EM}}}}/E_{0}),\log _{10}(\lambda _{\text {clus}}/\lambda _{0})\;\right\} , \end{aligned}$$

in this sequence mapped to bin indices $ijkl$ in the full accessible phase space. The density scale is $\rho _{0} = 1\,\text {MeV}\,{\text {mm}}^{-3}$, the signal normalisation is $E_{0} = 1\,\text {MeV}$, and longitudinal depth is measured in terms of $\lambda _{0} = 1\,{\text {mm}}$. Here the density $\rho _{\text {clus}}$ is divided by the cluster signal $E_{\text {clus}}^{{{\text {EM}}}}$. This provides a necessary reference scale for its evaluation. As an absolute measure, $\rho _{\text {clus}}$ is less powerful in separating electromagnetic from hadronic energy deposits, as the same densities can be generated by electromagnetically and hadronically interacting particles of different incident energies.

The likelihood $\mathcal {P}_{\text {clus}}^{\,{{\text {EM}}}}$ is defined in each bin $ijkl$ as

31 $$\begin{aligned} \mathcal {P}_{\text {clus}}^{\,{{\text {EM}}}}(E_{\text {clus}}^{{{\text {EM}}}},\eta _{\text {clus}},\rho _{\text {clus}}/E_{\text {clus}}^{{{\text {EM}}}},\lambda _{\text {clus}}) \mapsto \mathcal {P}_{\text {clus}, ijkl }^{\,{{\text {EM}}}} = \dfrac{\varepsilon ^{\pi ^{0}}_{ ijkl }}{\varepsilon ^{\pi ^{0}}_{ ijkl }+2\varepsilon ^{\pi ^{\pm }}_{ ijkl }}, \end{aligned}$$

with $0 \le \mathcal {P}_{\text {clus}, ijkl }^{\,{{\text {EM}}}} \le 1$. The efficiencies $\varepsilon _{ ijkl }^{\pi ^{0}(\pi ^{\pm })}$ are calculated as

32 $$\begin{aligned} \varepsilon _{ ijkl }^{\pi ^{0}(\pi ^{\pm })} = \dfrac{N_{ ijkl }^{\pi ^{0}(\pi ^{\pm })}}{N_{ ij }^{\pi ^{0}(\pi ^{\pm })}}. \end{aligned}$$

Here $N_{ ijkl }^{\pi ^{0}(\pi ^{\pm })}$ is the number of topo-clusters from $\pi ^{0}$ ($\pi ^{\pm }$) in a given bin $ijkl$, while $N_{ ij }^{\pi ^{0}(\pi ^{\pm })}$ is the number of $\pi ^{0}$ ($\pi ^{\pm }$) found in bin $ij$ of the $(E_{\text {clus}}^{{{\text {EM}}}},\eta _{\text {clus}})$ phase space. On average there is no detectable difference in the development of $\pi ^{+}$ and $\pi ^{-}$ initiated hadronic showers affecting the topo-cluster formation. The distributions of the observables in $\mathfrak {O}_{\text {clus}}^{\text {class}}$ as well as the correlations between them are the same. Therefore topo-clusters from $\pi ^{+}$ and $\pi ^{-}$ showers occupy the same bins in the $\mathfrak {O}_{\text {clus}}^{\text {class}}$ phase space, yielding $N_{ ijkl }^{\pi ^{\pm }} = N_{ ijkl }^{\pi ^{+}} = N_{ ijkl }^{\pi ^{-}}$, $N_{ ij }^{\pi ^{\pm }} = N_{ ij }^{\pi ^{+}} = N_{ ij }^{\pi ^{-}}$, and $\varepsilon ^{\pi ^{-}}_{ ijkl } + \varepsilon ^{\pi ^{+}}_{ ijkl } = 2\varepsilon ^{\pi ^{\pm }}_{ ijkl }$ in the definition of $\mathcal {P}_{\text {clus}}^{\,{{\text {EM}}}}$ in Eq. (31). This normalisation reflects the use of all three pion charges at equal probability in MC simulations, thus maintaining the correct isospin-preserving ratio.

For performance evaluation purposes, any topo-cluster with the set of observables $\mathfrak {O}_{\text {clus}}^{\text {class}}$ from Eq. (30) located in a bin $ijkl$ with $\mathcal {P}_{\text {clus}, ijkl }^{\,{{\text {EM}}}} \ge 0.5$ is classified as EM and with $\mathcal {P}_{\text {clus}, ijkl }^{\,{{\text {EM}}}} < 0.5$ is classified as HAD. In the rare case where a topo-cluster has too few cells or too little signal to meaningfully reconstruct the observables in $\mathfrak {O}_{\text {clus}}^{\text {class}}$, the cluster is likely generated by noise or insignificant energy deposits and is thus neither classified nor further corrected nor calibrated. An example of a $\mathcal {P}_{\text {clus}}^{\,{{\text {EM}}}}$ distribution in a given phase space bin $ij$ is shown in Fig. 10. All distributions and their bin contents are accessed as lookup tables to find $\mathcal {P}_{\text {clus}}^{\,{{\text {EM}}}}$ for a given cluster.

The distributions of $\lambda _{\text {clus}}$ for topo-clusters in jets reconstructed with the anti-$k_{t}$ algorithm with $R = 0.6$ are shown for clusters respectively classified as electromagnetic or hadronic, in 2010 data and MC simulations (no pile-up) in Figs. 11a and b. The specific structure of each distribution reflects the longitudinal segmentation of the electromagnetic and hadronic calorimeters in ATLAS. The average cluster depth $\langle \lambda _{\text {clus}}\rangle$ as a function of the cluster energy is shown in Figs. 11c and d for the same EM and HAD topo-clusters, respectively. The EM topo-clusters show the expected linear dependence of $\langle \lambda _{\text {clus}}\rangle$ on $\log {E_{\text {clus}}^{{{\text {EM}}}}}$ in Fig. 11c, with some modulations introduced by the read-out granularity of the EMC. The $\langle \lambda _{\text {clus}}\rangle$ dependence on $E_{\text {clus}}^{{{\text {EM}}}}$ shown for HAD topo-clusters in Fig. 11d features a similar shape up to $E_{\text {clus}}^{{{\text {EM}}}}\approx 2\,{\text {GeV}}$. This energy range is dominated by topo-clusters from low-energy hadrons, in addition to clusters from less-energetic hadronic shower fragments created by the splitting algorithm described in Sect. 3.1.3. For $E_{\text {clus}}^{{{\text {EM}}}}> 2\,{\text {GeV}}$ the average $\lambda _{\text {clus}}$ is increasingly dominated by higher-energy clusters produced by splitting and located in the electromagnetic calorimeter, thus pulling it to lower values. The rise of $\langle \lambda _{\text {clus}}\rangle$ for topo-clusters with $E_{\text {clus}}^{{{\text {EM}}}}\gtrsim 10\,{\text {GeV}}$ reflects increasing contributions from energetic hadrons with dense showers generating high-energy clusters deeper in the hadronic calorimeter. The good agreement between data and MC simulations for both classes of topo-clusters supports the use of $\lambda _{\text {clus}}$ for the cluster classification derived from MC simulations for data [38].

### Hadronic calibration

**Fig. 12:**
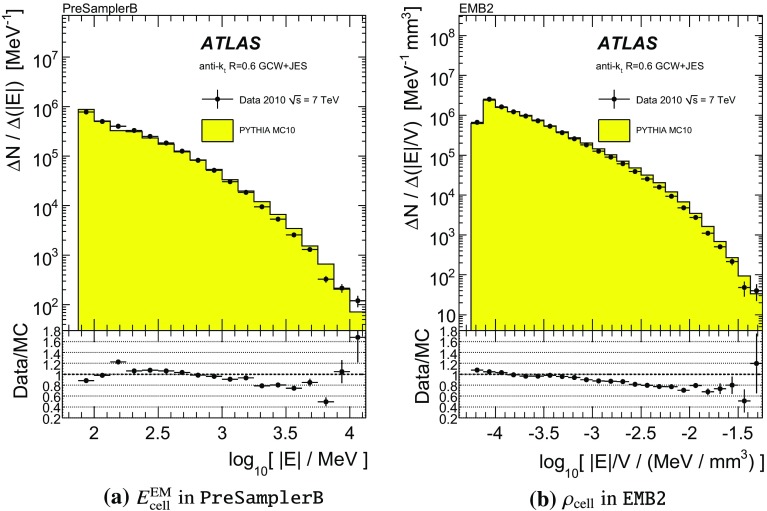
Distributions of the cell energy $E_{\text {cell}}^{{{\text {EM}}}}$ in the **a** central pre-sampler (PreSamplerB) and the cell energy density $\rho _{\text {cell}}$ in the second sampling of **b** the central (EMB2) electromagnetic calorimeter in ATLAS, as observed inside anti-$k_{t}$ jets with $R = 0.6$, calibrated with the global sequential (GCW+JES) calibration scheme described in Ref. [38], in 2010 data (no pile-up) and the corresponding MC simulations. The data/MC ratio of the spectra is shown below the corresponding distributions. The figure uses plots from Ref. [38]

The hadronic calibration for topo-clusters attempts to correct for non-compensating calorimeter response, meaning to establish an average $e/\pi = 1$ for the cluster signal. The calibration reference is the locally deposited energy in the cells of a given topo-cluster, which is defined as the sum of all energies released by various shower processes in these cells. In each of the cells, the signal $E_{\text {cell}}^{{{\text {EM}}}}$ from this deposited energy $E_{\text {cell}}^{\text {dep}}$ is reconstructed on the electromagnetic energy scale. This yields cell signal weights defined as

33 $$\begin{aligned} w_{\text {cell}}^{} = \dfrac{E_{\text {cell}}^{\text {dep}}}{E_{\text {cell}}^{{{\text {EM}}}}}. \end{aligned}$$

In the case of electromagnetic signals, $w_{\text {cell}}^{} = w_{\text {cell}}^{\text {em-cal}}{} \equiv 1$ by construction of the electromagnetic scale. In hadronic showers, $E_{\text {cell}}^{\text {dep}}$ has contributions from energy loss mechanisms which do not contribute to the signal, including nuclear binding energies and escaping energy carried by neutrinos. In this case, $w_{\text {cell}}^{} = w_{\text {cell}}^{\text {had-cal}}\ne 1$ with $w_{\text {cell}}^{\text {had-cal}}> 1$ for hadronic inelastic interactions within the cell volume, and $w_{\text {cell}}^{\text {had-cal}}< 1$ for deposits by ionisations.^13^ The appropriate value of $w_{\text {cell}}^{\text {had-cal}}$ reflecting on average the energy loss mechanism generating $E_{\text {cell}}^{{{\text {EM}}}}$ in a given cell is determined by the hadronic calibration as a function of a set of observables $\mathfrak {O}_{\text {cell}}^{\text {had-cal}}$ associated with the cell and the topo-cluster it belongs to. It is then applied to $E_{\text {cell}}^{{{\text {EM}}}}$ according to Eq. (29) in the signal reconstruction.

**Fig. 13:**
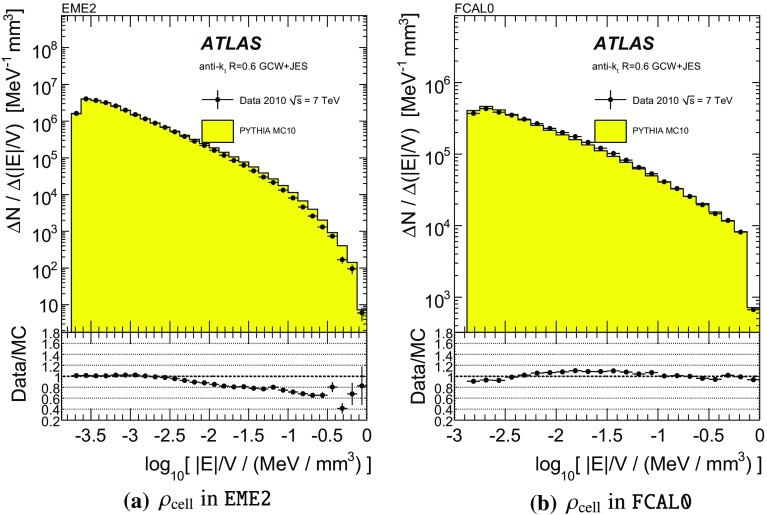
Distributions of the cell energy density $\rho _{\text {cell}}$ in the **a** second sampling of the end-cap (EME2) electromagnetic calorimeter, and the **b** first module of the forward calorimeter (FCAL0) in ATLAS, as observed inside anti-$k_{t}$ jets with $R = 0.6$, calibrated with the GCW + JES scheme described in Ref. [38], in 2010 data and MC simulations (no pile-up). The data/MC ratio of the spectra is shown below the corresponding distributions. The figure uses plots from Ref. [38]

Simultaneously using all simulations of charged single pions for all energies and directions, lookup tables are constructed from binned distributions relating $\mathfrak {O}_{\text {cell}}^{\text {had-cal}}$, defined as

34 $$\begin{aligned} \mathfrak {O}_{\text {cell}}^{\text {had-cal}} = \left\{ \;S_{\text {calo}},\;\eta _{\text {cell}},\;\log _{10}(\rho _{\text {cell}}/\rho _{0}),\;\log _{10}(E_{\text {clus}}^{{{\text {EM}}}}/E_{0})\;\right\} , \end{aligned}$$

to the hadronic signal calibration weight $w_{\text {cell}}^{\text {had-cal}}$. The cell location is defined by one of the sampling layer identifiers $S_{\text {calo}}$ listed in Table 1 in Sect. 2.1 and the direction of the cell centre $\eta _{\text {cell}}$ extrapolated from the nominal detector centre of ATLAS. The cell signal density $\rho _{\text {cell}}$ is measured as discussed in Sect. 4.2.2, and $E_{\text {clus}}^{{{\text {EM}}}}$ is the signal of the topo-cluster to which the cell contributes to. The lookup tables are binned in terms of $\mathfrak {O}_{\text {cell}}^{\text {had-cal}}$ such that $w_{\text {cell}}^{\text {had-cal}}$ in each bin in the filled table is the average over all cells with observables fitting into this bin, with each contributing weight calculated as given in Eq. (33). These average weights are then retrieved for any cell in a topo-cluster as a function of $\mathfrak {O}_{\text {cell}}^{\text {had-cal}}$. The cluster signal and directions are re-summed as discussed in Sect. 5.6. The scales $\rho _{0}$ and $E_{0}$ in Eq. (34) are the same as the ones used in Eq. (30).

**Fig. 14:**
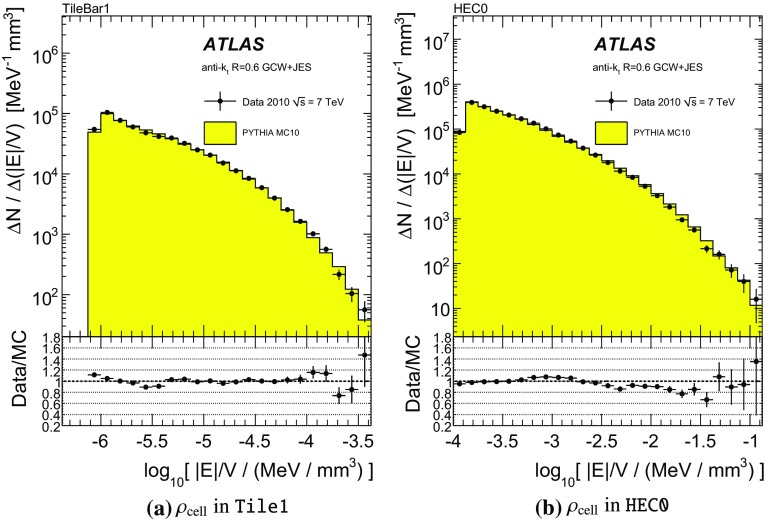
Distributions of the cell energy density $\rho _{\text {cell}}$ in the central **a** and end-cap **b** hadronic calorimeters in ATLAS, as observed inside anti-$k_{t}$ jets with $R = 0.6$ calibrated with the GCW + JES scheme described in Ref. [38], in 2010 data and MC simulations (no pile-up). The data/MC ratio of the spectra is shown below the corresponding distributions. The figure uses plots from Ref. [38]

The $E_{\text {cell}}^{{{\text {EM}}}}$ distribution in the PreSampler and the $\rho _{\text {cell}}$ distribution in the the EMB2 sampling of the central electromagmetic calorimeter are shown in Fig. 12 for cells in topo-clusters inside jets reconstructed with the anti-$k_{t}$ algorithm using a distance parameter $R = 0.6$. Discrepancies between data and MC simulations mostly in the high-end tails of the distributions indicate more compact electromagnetic showers in the simulation. This is also seen in Fig. 13a for the $\rho _{\text {cell}}$ distribution for the same kind of jets in the EME2 sampling of the electromagnetic end-cap calorimeter. Better agreement between data and MC simulations over the whole spectrum is observed for the $\rho _{\text {cell}}$ distributions in the first module (FCAL0) of the forward calorimeter shown in Fig. 13b, and in the second sampling of the central hadronic (Tile1) and the first sampling of the hadronic end-cap (HEC0) calorimeters shown in Fig. 14. Overall, the quality of the modelling of the cell signal densities is sufficient for topo-cluster calibration purposes. The figures are taken from Ref. [38].

### Correction for out-of-cluster signal losses

In the process of applying the noise suppression described in Sect. 3.1, cells with small true deposited energy generated by EM or HAD showers may not be collected into a topo-cluster, either due to lack of significance of their small signal, or due to the absence of a neighbouring cell with a significant signal. The energy losses introduced by this effect are estimated using single-particle MC simulations. A corresponding *out-of-cluster* correction is determined and applied to nearby topo-clusters. The cells with true energy not included into clusters are referred to as *lost cells*.

**Fig. 15:**
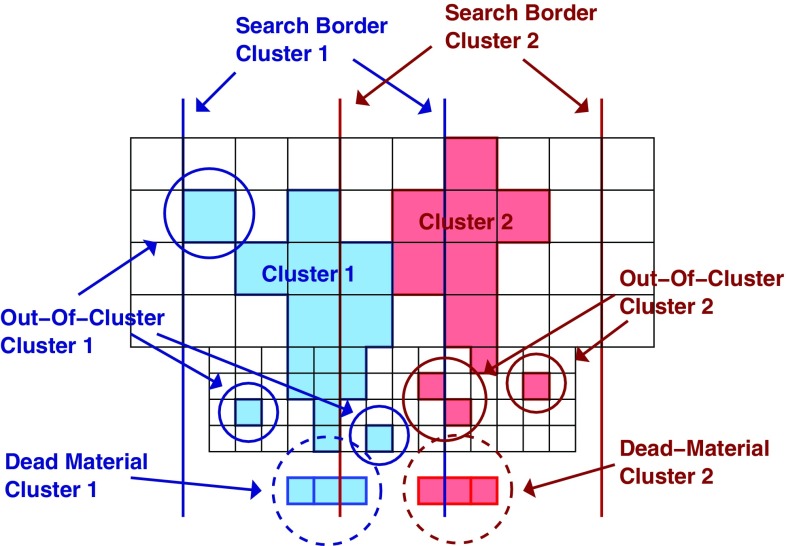
Illustration of the assignment scheme for cells inside the calorimeter with true signal not captured in a topo-cluster in the context of the out-of-cluster correction (see Sect. 5.4) and for dead material cells outside the calorimeter for the dead material correction discussed in Sect. 5.5. The deposited energy in cells inside the topo-cluster is used to determine the hadronic calibration described in Sect. 5.3. A schematic depiction of a typical section of the ATLAS end-cap calorimeter with four highly granular electromagnetic samplings and four coarser hadronic samplings is shown in a view with $\eta$ as the *horizontal* and the depth *z* as the *vertical* coordinate. The *boxes* at small *z* in front of the EM calorimeter symbolise upstream energy losses collected into dead material cells

The challenge in determining this correction is the assignment of the energy deposited in a lost cell to a certain cluster. As discussed in Sect. 3.1.3 and seen in Fig. 7, hadronic showers in particular can generate more than one topo-cluster. An algorithm defining an *out-of-cluster neighbourhood* to search for the lost cells has been developed for this assignment. This is depicted schematically in Fig. 15. The actual size of the neighbourhood for a given topo-cluster is determined by the maximum angular distance between the cluster and the lost cells. This distance depends on $\eta _{\text {clus}}$, and thus reflects granularity changes and shower size variations. It varies from approximately $\pi /3\,{{\mathrm{rad}}}$ ($60^{\circ }$) at $\eta _{\text {clus}}= 0$ to $7\pi /90\,{{\mathrm{rad}}}$ ($14^{\circ }$) for $\eta _{\text {clus}}> 3.2$. The energy $E_{\text {clus}}^{\text {ooc}}$ deposited in all lost cells associated with a given topo-cluster is then used to derive the out-of-cluster correction factor $w_{\text {clus}}^{\text {ooc}}$,

35 $$\begin{aligned} w_{\text {clus}}^{\text {ooc}}= \dfrac{E_{\text {clus}}^{\text {ooc}}+ E_{\text {clus}}^{\text {dep}}}{E_{\text {clus}}^{\text {dep}}} \quad \text {and}\quad E_{\text {clus}}^{\text {ooc}}= \sum _{i \in {\{ \mathrm{lost cells} \}}} E_{\text {cell},\text {lost},i}^{\text {dep}}. \end{aligned}$$

Here $E_{\text {clus}}^{\text {dep}}$ is the summed deposited energy of all cells inside the cluster. The out-of-cluster correction is a cluster-level correction featuring $w_{\text {clus}}^{\text {ooc}}\ge 1$.

Figure 15 shows that a lost cell can be located in the two overlapping out-of-cluster neighbourhoods of two close-by topo-clusters. In this case $E_{\text {cell},\text {lost}}^{\text {dep}}$ of this lost cell is assigned to both clusters, with a weight proportional to their respective deposited energies $E_{\text {clus},1(2)}^{\text {dep}}$. The out-of-cluster correction takes into account shared and non-shared lost cells and is derived for each of the two clusters separately using Eq. (35) with36There are no spatial distance criteria applied to the sharing.

The scheme for the out-of-cluster correction ignores lost energy deposited in inactive areas of the detector, outside calorimeter cells. This effect is corrected for later in the calibration sequence (see Sect. 5.5) such that this component is not double-counted.

The out-of-cluster correction is different for electromagnetic and hadronic showers and is therefore separately determined with neutral and charged pion single-particle simulations. The three-dimensional set of observables $\mathfrak {O}_{\text {clus}}^{\text {ooc}}$

37 $$\begin{aligned} \mathfrak {O}_{\text {clus}}^{\text {ooc}} = \left\{ \;\eta _{\text {clus}},\;\log _{10}(E_{\text {clus}}^{{{\text {EM}}}}/E_{0}),\;\log _{10}(\lambda _{\text {clus}}/\lambda _{0})\;\right\} \end{aligned}$$

is used to bin $w_{\text {clus}}^{\text {ooc}}$. The weight is applied to the signal of nearly all cells of the topo-cluster receiving the out-of-cluster correction such that $w_{\text {cell}}^{\text {ooc}}= w_{\text {clus}}^{\text {ooc}}$. The exceptions are cells located in the LAr pre-samplers PreSamplerB and PreSamplerE, and the Tile scintillators located between the barrel and end-cap cryostats, where $w_{\text {cell}}^{\text {ooc}}= 1$ always. The normalisations $E_{0}$ and $\lambda _{0}$ in Eq. (37) are the same as used in Eq. (30).

**Fig. 16:**
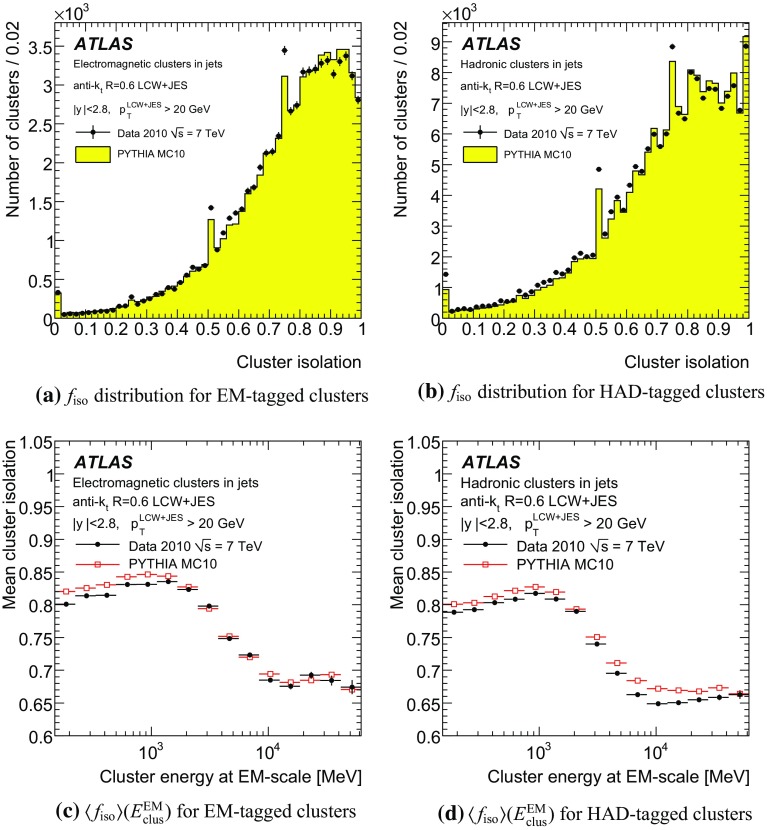
The distribution of the isolation moment $f_{\text {iso}}$ in **a** clusters classified as electromagnetic, and **b** clusters classified as hadronic. The average isolation $\langle f_{\text {iso}}\rangle$ as a function of the cluster signal $E_{\text {clus}}^{{{\text {EM}}}}$ is shown in **c** for electromagnetic and in **d** for hadronic topo-clusters. The figures are taken from Ref. [38]

While the determination of the out-of-cluster correction depends on this assignment algorithm, the application of the correction is context dependent. A topo-cluster in a jet is likely to have directly neighbouring clusters which can capture its out-of-cluster signal loss. It is therefore expected that topo-clusters in jets need less out-of-cluster corrections than isolated topo-clusters away from other clusters. The degree of isolation is measured by the isolation moment $f_{\text {iso}}$ introduced in Sect. 4.2.5. The out-of-cluster correction is effectively $f_{\text {iso}} w_{\text {clus}}^{\text {ooc}}(\mathfrak {O}_{\text {clus}}^{\text {ooc}})$. This correction can change the barycentre and centre of gravity of topo-clusters containing cells from the LAr pre-samplers or the Tile scintillators.

Figure 16 shows $f_{\text {iso}}$ for topo-clusters classified as either electromagnetic or hadronic in jets reconstructed with the anti-$k_{t}$ algorithm and $R = 0.6$ [38]. A good agreement between data and MC simulations is observed, both for the details of the respective $f_{\text {iso}}$ in Figs. 16a and b and the average as a function of $E_{\text {clus}}^{{{\text {EM}}}}$ in Figs. 16c and d. The $E_{\text {clus}}^{{{\text {EM}}}}$ dependence of $f_{\text {iso}}$ is very similar for both kinds of topo-clusters.

The peak structure in the $f_{\text {iso}}$ distributions shown in Figs. 16a and b is indicative of topo-clusters which have a large fraction of their energy in one sampling layer in the (regular) ATLAS calorimeter read-out segmentation with at least 16 cells around the perimeter of clustered cells in a sampling layer. The isolation of this layer then dominates the overall $f_{\text {iso}}$, as given by Eq. (28) in Sect. 4.2.5. This dominance of just one sampling layer with the minimal number of cells is typical for topo-clusters seeded by a cell barely above the seed threshold defined in Eq. (3) and too little energy in the neighbouring samplings to further expand the cluster. Neighbouring cells then limit $f_{\text {iso}}$ to the multiples of 1 / 16 visible in Figs. 16a and b. Even multiples of 1 / 16 occur more often than odd multiples since they can be produced more easily by topo-clusters with a different number of neighbours. The fact that clusters close to the noise threshold are mainly responsible for the peaks explains the mismatch between data and MC simulations observed in the peak heights, and points to non-perfect modelling of noise and very small signals. The overall structure of the $f_{\text {iso}}$ spectrum in data is well reproduced in terms of the peak locations by MC simulations.

### Dead material corrections

**Fig. 17:**
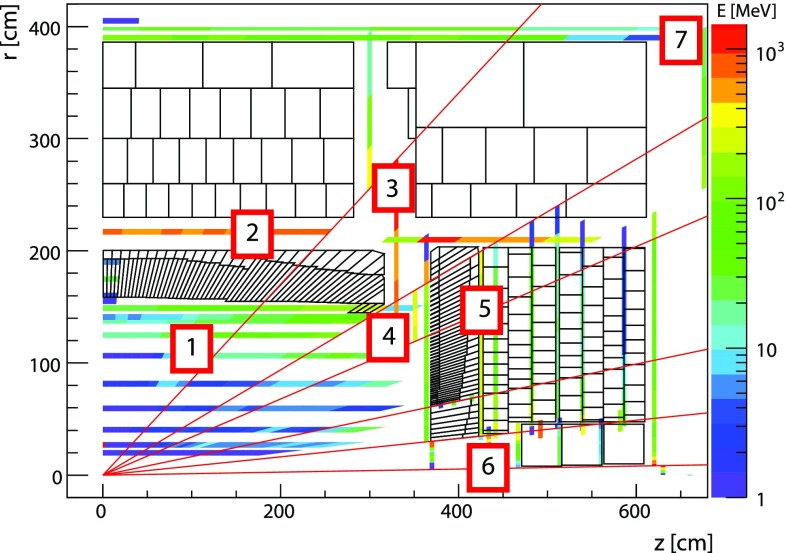
The average energy loss in the virtual dead material cells for charged 100 GeV pions. The numbers 1–7 indicate the different regions, with region 8 (not displayed) being everywhere outside regions 1–7. The dead material cells are superimposed on a schematic (*r*, *z*) view showing a quarter of the ATLAS calorimeter system with its read-out segmentation

Particles traversing the inactive (dead) material in front of or between calorimeter modules can deposit energy in it, thus reducing the measurable energy. This energy loss is addressed on average by the dead material correction. It is derived with single-particle MC simulations, where the deposited energy in the dead material outside of the active calorimeter can be calculated. This material is divided into virtual cells with a pointing geometry in $(\eta ,\phi )$. These cells are similar to the ATLAS calorimeter cells, but typically larger in size. Depending on the particle’s direction of flight, eight distinct regions are mapped out, as summarised in Table 2. The energy deposited in the dead material cells is determined for charged and neutral pions at various energies and directions, and almost everywhere correlated with measurable signals.

Figure 17 shows a projection of the dead material cells where energy loss is recorded to determine the dead material correction. The assignment to a topo-cluster is based on the same search-border strategy used for the determination of the out-of-cluster correction and illustrated in Fig. 15, with a refinement of the assignment procedure specific for the determination of dead material corrections. Instead of using the full deposited energy $E_{\text {clus}}^{\text {dep}}$ in the topo-cluster as input for sharing in Eq. (35), the energy $E_{\text {clus}}^{\text {dep}}(s)$ deposited in a selected sampling layer *s* is used to assign the dead material energy to topo-clusters. For a given cluster *k* out of $N_{\text {clus}}$ topo-clusters which have cells from *s* included, the assignment weight *w* is calculated using

38 $$\begin{aligned} w = \dfrac{\sqrt{E_{\text {clus},k}^{\text {dep}}(s)}\times \exp (-\Delta R_{k}/R_{0})}{\displaystyle \sum _{i=1}^{N_{\text {clus}}}\sqrt{E_{\text {clus},i}^{\text {dep}}(s)}\times \exp (-\Delta R_{i}/R_{0})}, \quad \text {with}\quad \Delta R_{k(i)} = \sqrt{(\Delta \eta _{k(i)})^{2}+(\Delta \phi _{k(i)})^{2}} \quad \text {and}\quad R_{0} = 0.2. \end{aligned}$$

The choice of *s* depends on the dead material regions indicated in Fig. 17. The distances $\Delta \eta$ and $\Delta \phi$ are measured between the topo-cluster direction and the dead material cell direction. The normalisation of *w* is calculated using all $N_{\text {clus}}$ clusters such that $0 \le w \le 1$. It is rare that two clusters are close to the same dead material cell, most often $w = 1$ is found for the closest topo-cluster, and $w = 0$ for the next closest ones.

This weighted energy loss is collected as a function of observables of the associated topo-cluster given in Table 2. Lost energy deposited in front of the calorimeter is compensated for by applying a correction proportional to the pre-sampler signals in topo-clusters which contain these signals. In the forward region the signal in the first module FCAL0 of the FCAL is used for this purpose.

**Table 2:** Overview of the signals used to correct for dead material losses in the various regions around the ATLAS calorimeters. The numbered regions are shown in Fig. 17. The parameter values used for the dead material correction are extracted from lookup tables. Region 8 comprises all dead material volumes with energy loss outside regions 1–7. These are mostly small volumes located between and behind the active calorimeters

Regions	Description	Cluster signals for dead material correction
1	In front of EMB	Energy in PreSamplerB
2	Between EMB and Tile	Energies in last layer of EMB and first layer of Tile
3	In front of Tile gap scintillators	Energy in Tile gap scintillators
4	In front of EMEC	Energy in PreSamplerE
5	Between EMEC and HEC	Energies in last layer of EMEC and first layer of HEC
6	In front of FCAL	Energy in first FCAL module
7	Behind calorimeters	Energy in last layer of hadronic calorimeters and $\mathfrak {O}_{\text {clus}}^{\text {dm}}$ given in Eq. (39)
8	Everywhere else	

Energy lost between an electromagnetic and a hadronic calorimeter module (regions 2 and 5 in Table 2; Fig. 17) is found to be proportional to $\sqrt{E_{\text {l}}^{{{\text {EM}}}} \cdot E_{\text {f}}^{{{\text {EM}}}}}$, where $E_{\text {l}}^{{{\text {EM}}}}$ is the energy in the last sampling layer of the electromagnetic calorimeter, and $E_{\text {f}}^{{{\text {EM}}}}$ is the energy in the first sampling layer of the hadronic calorimeter. Both $E_{\text {l}}^{{{\text {EM}}}}$ and $E_{\text {f}}^{{{\text {EM}}}}$ are reconstructed on the electromagnetic energy scale. This correction is only applied to topo-clusters which span the material between the two calorimeters.

Dead material corrections for longitudinal leakage (region 7 in Table 2; Fig. 17) are applied to topo-clusters that contain cells from the very last (hadronic) calorimeter sampling layer. These corrections are calculated in three-dimensional bins of a set of observables $\mathfrak {O}_{\text {clus}}^{\text {dm}}$, with

39 $$\begin{aligned} \mathfrak {O}_{\text {clus}}^{\text {dm}} = \left\{ \;\eta _{\text {clus}},\;\log _{10}(E_{\text {clus}}^{{{\text {EM}}}}/E_0),\;\lambda _{\text {clus}}\;\right\} , \end{aligned}$$

and $E_{0}$ from Eq. (30) in Sect. 5.2. The same set of observables is used as input to correct dead material energy losses in topo-clusters that are located in the direct neighbourhood of inactive material categorised as region 8 and that have no other dead material correction applied.

Like the out-of-cluster correction, the dead material correction is a cluster-based correction. It is expressed in terms of a weight $w_{\text {clus}}^{\text {dm}}$, which is determined from the various correction functions or lookup tables. The corresponding cell signal weight is the same for all cells of the given cluster ($w_{\text {cell}}^{\text {dm}}= w_{\text {clus}}^{\text {dm}}$). This correction therefore does not affect the topo-cluster barycentre or centre of gravity.

### Fully calibrated cluster kinematics

The reconstructed and fully calibrated topo-cluster energy $E_{\text {clus}}^{{{\text {LCW}}}}$ depends on the EM likelihood of the cluster, as discussed in Sect. 5.2, and is characterised by $E_{\text {clus}}^{{{\text {LCW}}}}\ge E_{\text {clus}}^{{{\text {EM}}}}$. The cluster direction changes due to the calibration, because it is calculated from energy-weighted cell directions using Eqs. (9) and (10) with $w_{\text {cell}}^{\text {geo}}\rightarrow w_{\text {cell}}^{\text {cal}}$.

The effective cell calibration weight $w_{\text {cell}}^{\text {cal}}$ from Eq. (29) after any of the calibrations or corrections are applied yields the cluster energy $E_{\text {clus}}^{\text {cal}}$ after the calibration

40 $$\begin{aligned} E_{\text {clus}}^{\text {cal}}= \sum _{i\,\in \mathrm{cluster}} w_{\text {cell},i}^{\text {cal}} E_{\text {cell},i}^{\text {EM}}. \end{aligned}$$

While the signal weights determined for each calibration and correction are independently derived, the overall effect of the calibration sequence leads to a factorised accumulation of $w_{\text {cell}}^{\text {cal}}$ in the reconstruction of the cell energies. This is summarised in Table 3. The overall weight $w_{\text {cell}}^{\,{{\text {LCW}}}}$ given in item (5) of the table is used cell-by-cell in Eq. (40) to calculate the final cluster energy $E_{\text {clus}}^{{{\text {LCW}}}}$ by setting $w_{\text {cell},i}^{\text {cal}} = w_{\text {cell},i}^{\,{{\text {LCW}}}}$ and thus yielding $E_{\text {clus}}^{{{\text {LCW}}}}= E_{\text {clus}}^{\text {cal}}$. As discussed earlier, $w_{\text {cell}}^{\,{{\text {LCW}}}}$ is also used to recalculate the cluster directions $\eta _{\text {clus}}$ and $\phi _{\text {clus}}$. The final fully calibrated four-momentum reconstructed for any topo-cluster is given by replacing $E_{\text {clus}}^{{{\text {EM}}}}$ in Eq. (12) in Sect. 3.2 with $E_{\text {clus}}^{{{\text {LCW}}}}$.

**Table 3:** Summary of the calibration and correction sequence applied to topo-clusters from the EM to the final LCW scale.

Procedure	Parameters	Effective cell signal weight after each step
(1) Cluster formation	$w_{\text {cell}}^{\text {geo}}$	$w_{\text {cell}}^{\text {geo}}$
(2) Classification	$\mathcal {P}_{\text {clus}}^{\,{{\text {EM}}}}$	$w_{\text {cell}}^{\text {geo}}$
(3) Calibration	$w_{\text {cell}}^{\text {em-cal}}(= 1)$	$w_{\text {cell}}^{\text {geo}}\left[ \mathcal {P}_{\text {clus}}^{\,{{\text {EM}}}}\,w_{\text {cell}}^{\text {em-cal}}+ ( 1-\mathcal {P}_{\text {clus}}^{\,{{\text {EM}}}})\, w_{\text {cell}}^{\text {had-cal}}\right]$
	$$w_{\text {cell}}^{\text {had-cal}}$$	
(4) Out-of-cluster	$w_{\text {cell}}^{\text {em-ooc}}$	$\displaystyle {w_{\text {cell}}^{\text {geo}}\prod _{\kappa \,\in \{\text {cal},\text {ooc}\}} \left[ \mathcal {P}_{\text {clus}}^{\,{{\text {EM}}}}\cdot w_{\text {cell}}^{\text {em}-\kappa }+ ( 1-\mathcal {P}_{\text {clus}}^{\,{{\text {EM}}}})\cdot w_{\text {cell}}^{\text {had-}\kappa }\right] }$
	$$w_{\text {cell}}^{\text {had-ooc}}$$	
(5) Dead material	$w_{\text {cell}}^{\text {em-dm}}$	$w_{\text {cell}}^{\,{{\text {LCW}}}}= \displaystyle {w_{\text {cell}}^{\text {geo}}\prod _{\kappa \,\in \{\text {cal},\text {ooc},\text {dm}\}} \left[ \mathcal {P}_{\text {clus}}^{\,{{\text {EM}}}}\,w_{\text {cell}}^{\text {em}-\kappa }+ ( 1-\mathcal {P}_{\text {clus}}^{\,{{\text {EM}}}})\,w_{\text {cell}}^{\text {had-}\kappa }\right] }$
	$$w_{\text {cell}}^{\text {had-dm}}$$	

**Fig. 18:**
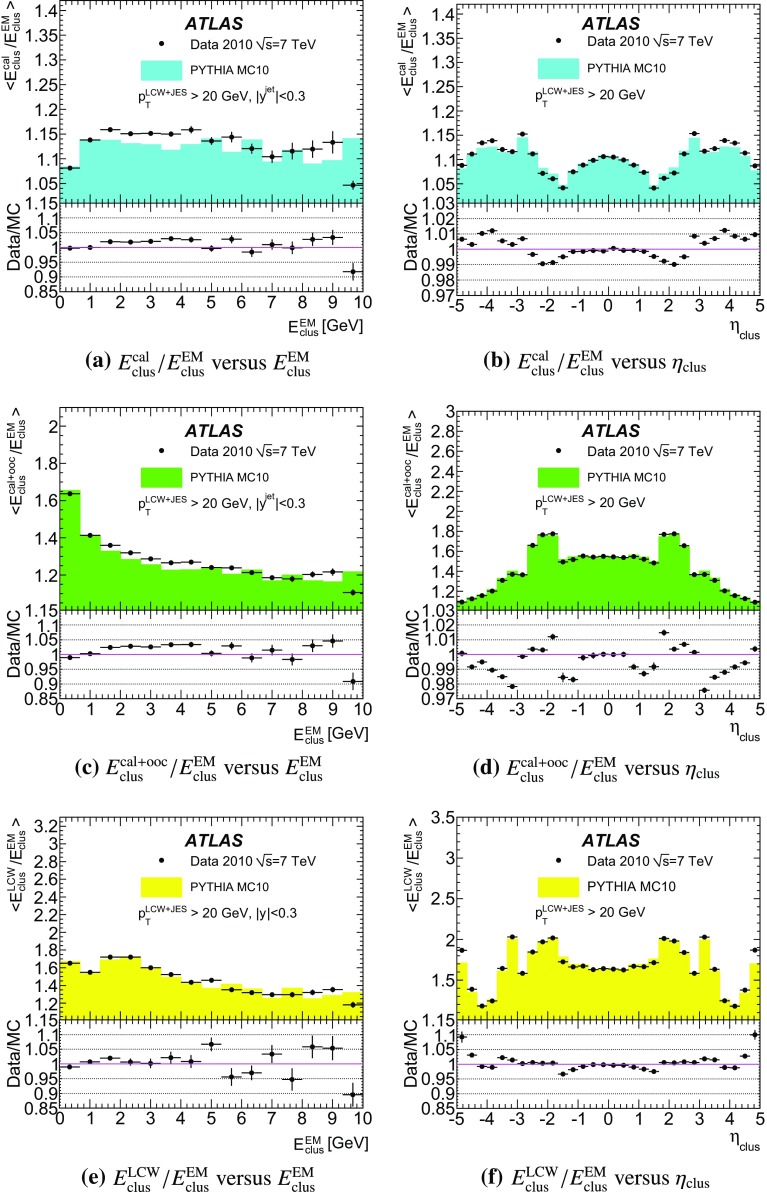
The average ratio of reconstructed to EM-scale energy after each calibration step, as a function of the cluster energy $E_{\text {clus}}^{{{\text {EM}}}}$ (**a**, **c**, **e**) for topo-clusters in anti-$k_{t}$ jets with $R =0.6$ and $p_{\text {T}}> 20$ GeV and with rapidities $|y_{\text {jet}}| < 0.3$. The corresponding average ratios as a function of $\eta _{\text {clus}}$ are shown in **b**, **d**, and **f**. Data recorded in 2010 is compared to the corresponding MC simulations. The figures are adapted from Ref. [38]

All input parameter values used in the LCW calibration are derived from dedicated single-particle MC simulations. The validity of this calibration is confirmed with data, where the cumulative effect of the hadronic calibration and the out-of-cluster and dead material corrections on the signal of topo-clusters found in jets is analysed and compared to corresponding MC simulations. Figure 18 summarises the quality of the LCW calibration for these clusters, both as a function of the basic cluster signal $E_{\text {clus}}^{{{\text {EM}}}}$ and the cluster direction $\eta _{\text {clus}}$ [38]. Data are compared to MC simulations after the application of the hadronic cell weights ($E_{\text {clus}}^{\text {cal}}/E_{\text {clus}}^{{{\text {EM}}}}$ in Fig. 18a, b), followed by the out-of-cluster correction ($E^{\text {cal}+\text {ooc}}_{\text {clus}}/E_{\text {clus}}^{{{\text {EM}}}}$ in Fig. 18c, d), and at the LCW scale after applying the dead material correction ($E_{\text {clus}}^{{{\text {LCW}}}}/E_{\text {clus}}^{{{\text {EM}}}}$ in Fig. 18e, f). The differences between data and MC simulations are determined from these results as functions of $E_{\text {clus}}^{{{\text {EM}}}}$ and $\eta _{\text {clus}}$ using the respective double-ratio

$$\begin{aligned} \dfrac{\left\langle E_{\text {clus}}^{\text {cal}}/E_{\text {clus}}^{{{\text {EM}}}}\right\rangle _{\text {data}}}{\left\langle E_{\text {clus}}^{\text {cal}}/E_{\text {clus}}^{{{\text {EM}}}}\right\rangle _{\mathrm{MC}}},\quad \dfrac{\left\langle E^{\text {cal}+\text {ooc}}_{\text {clus}}/E_{\text {clus}}^{{{\text {EM}}}}\right\rangle _{\text {data}}}{\left\langle E^{\text {cal}+\text {ooc}}_{\text {clus}}/E_{\text {clus}}^{{{\text {EM}}}}\right\rangle _{\mathrm{MC}}}, \quad \text {and} \quad \dfrac{\left\langle E_{\text {clus}}^{{{\text {LCW}}}}/E_{\text {clus}}^{{{\text {EM}}}}\right\rangle _{\text {data}}}{\left\langle E_{\text {clus}}^{{{\text {LCW}}}}/E_{\text {clus}}^{{{\text {EM}}}}\right\rangle _{\mathrm{MC}}}. \end{aligned}$$

These double-ratios are shown in Fig. 18 as well, and indicate generally good agreement between data and MC simulations. The particular structures shown in the $\eta _{\text {clus}}$ dependence of the magnitude of the various calibration steps indicate the cumulative effects of transition regions between calorimeters in ATLAS, due to not only technology changes but also to changes in the read-out granularity. Especially Fig. 18f shows the large correction factors applied by the LCW calibration in the attempt to recover signal losses introduced by (1) the transition between the central and the end-cap calorimeters at $|\eta | \approx 1.45$, (2) the transition between end-cap and forward calorimeters at $|\eta |\approx 3.2$, and (3) the upper limit of the ATLAS calorimeter acceptance at $|\eta |\approx 4.9$.

## Performance of the simulation of topo-cluster kinematics and properties

The reconstruction performance of the topological cell clustering algorithm in ATLAS can be evaluated in the context of reconstructed physics objects such as jets or (isolated) single particles. In addition, features of the topo-cluster signal outside these physics objects can be studied with exclusive samples of low-multiplicity final states without jets. These are preferably selected by muons as those leave only small signals in the calorimeter, nearly independent of their $p_{\text {T}}$ ($W \!\rightarrow \!\ell \nu$ or $Z \!\rightarrow \!\mu \mu$ without jets). The topo-clusters not used in reconstructing hard physics objects reflect the calorimeter sensitivity to small and dispersed energy flows generated by the proton–proton collisions in the LHC, including pile-up. The level of agreement between data and MC simulations is used in all cases as a metric for the reconstruction performance.

### Single-particle response

The calorimeter response to single isolated charged hadrons with well-measured momentum in the ID was determined using proton–proton collision data at $\sqrt{s} = 900$ GeV in 2009 [45]. The single-hadron response at higher centre-of-mass energies was determined in 2010 at $\sqrt{s} = 7\,{\text {TeV}}$ and in 2012 at $\sqrt{s} = 8\,{\text {TeV}}$ [46]. Due to the relatively low luminosities in the 2009 and 2010 run periods, pile-up contributions are insignificant in the corresponding data. These measurements provide important validations of the topo-cluster algorithm and the calorimeter acceptance in general.

**Fig. 19:**
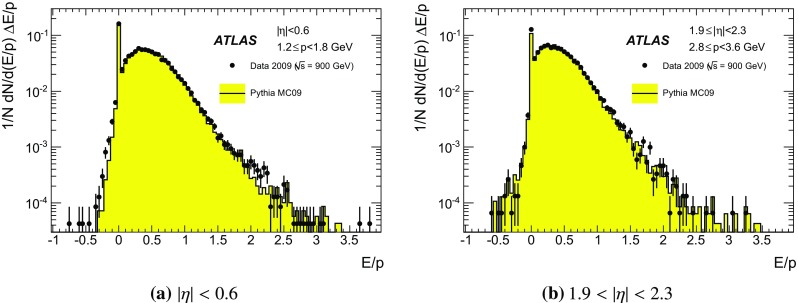
The distribution of $E/p$, the ratio of the calorimeter energy *E* and the track momentum *p*, for (**a**) central tracks with $1.2\,{\text {GeV}}< p < 1.8\,{\text {GeV}}$ and (**b**) forward-going tracks with $2.8\,{\text {GeV}}< p < 3.6\,{\text {GeV}}$, for data and MC simulations of proton–proton collisions at $\sqrt{s} = 900\,{\text {GeV}}$ and no pile-up (from Ref. [38])

The principal observable is the energy-to-momentum ratio $E/p$. The calorimeter energy *E* is reconstructed using the topo-clusters located around the direction of the track of the incoming charged particle with momentum *p*, including the ones with $E_{\text {clus}}^{{{\text {EM}}}}< 0$. The effect of the axial magnetic field is taken into account by extrapolating the reconstructed tracks into the calorimeter. The energy *E* is then calculated by summing the EM-scale energies from all sampling layers *s* of topo-clusters which have a barycentre $(\eta _{s},\phi _{s})$ within $\Delta R = 0.2$ of the track direction extrapolated to each *s*, as described in more detail in Ref. [45]. The sampling layer energies are summed irrespective of their sign, i.e. $E<0$ is possible.

The results of the measurement of $E/p$ are shown in Figs. 19a and b for reconstructed isolated tracks in proton–proton collisions at $\sqrt{s} = 900$ GeV. The distributions reflect the acceptance of the calorimeter for charged particles in the given momentum ranges. Entries for $E/p< 0$ indicate that the incoming track is matched with a topo-cluster generated by significant electronic noise. The number of tracks with no matching calorimeter signal ($E = 0 \Rightarrow E/p= 0$) is indicative of none or only a small fraction of the particle energy reaching the calorimeter, and the signal generated by this energy fraction is not sufficiently significant to survive the implicit noise suppression in the topo-cluster formation described in Sect. 3.1.

**Fig. 20:**
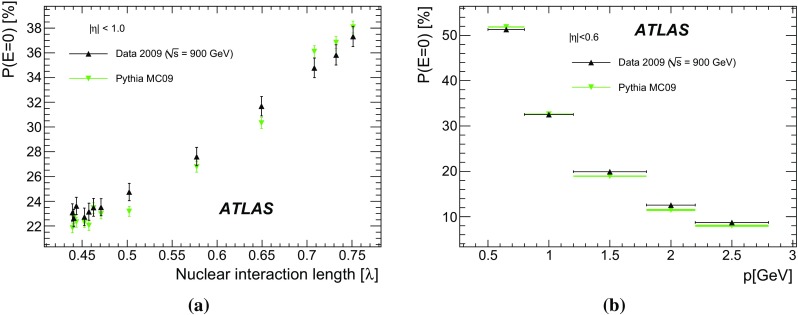
In **a**, the likelihood $\mathcal {P}_{E = 0}(d_{\text {dm}})$ to find no matching energy in the calorimeter ($E = 0$) for reconstructed isolated charged-particle tracks is shown as a function of the thickness $d_{\text {dm}}$ of the inactive material in front of the calorimeter, for data and MC simulations in proton–proton collisions at $\sqrt{s} = 900\,{\text {GeV}}$. The thickness of the inactive material is measured in terms of the nuclear interaction length $\lambda _{\text {nucl}}$. The tracks are reconstructed within $|\eta | < 1$. The likelihood to reconstruct $E = 0$ as a function of the incoming track momentum is shown for the same data and MC simulations in **b**, for reconstructed tracks within $|\eta | < 0.6$. Both figures are taken from Ref. [38]

The likelihood $\mathcal {P}_{E=0}(d_{\text {dm}})$ to find $E = 0$ for a charged particle passing through inactive material of various thicknesses $d_{\text {dm}}$, measured in terms of the nuclear interaction length $\lambda _{\text {nucl}}$, is shown in Fig. 20a for isolated tracks within $|\eta | < 1.0$ in proton–proton collisions at $\sqrt{s} = 900\,{\text {GeV}}$. The various values of $d_{\text {dm}}$ are extracted from the detector description in the MC simulation using the direction $|\eta |$ of the incoming tracks. The data and MC simulations agree well, indicating an appropriate description of the actual detector geometry in the MC simulation. The likelihood to have no matching signal in the calorimeter shows the expected increase with increasing inactive material.

The dependence of $\mathcal {P}_{E=0}$ on the track momentum is shown in Fig. 20b for isolated tracks with $|\eta | < 0.6$. Good agreement between data and MC simulations is observed, which together with the results displayed in Figs. 19 and 20a indicates a good description of the data by the QGSP_BERT hadronic shower model used by the MC simulation.

**Fig. 21:**
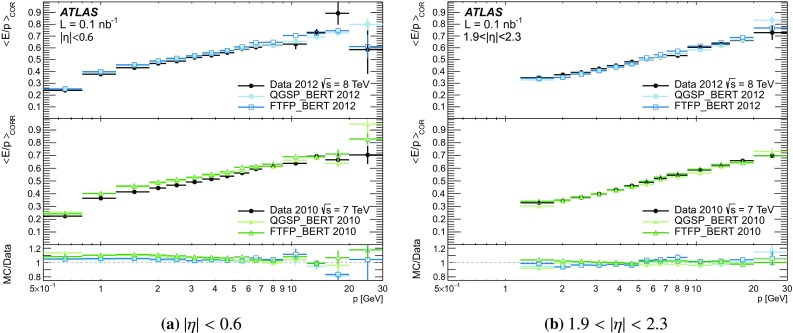
The average $\langle E/p\rangle$ ratio as a function of the track momentum *p*, for **a** tracks within $|\eta |<0.6$ and **b** tracks within $1.9< |\eta | < 2.3$. Data from isolated tracks recorded in 2010 and 2012 with insignificant pile-up are shown together with MC simulations employing two different hadronic shower models

The dependence of $E/p$ on the track momentum has been evaluated for two different hadronic shower models in Geant4. In addition to the default QGSP_BERT model introduced in Sect. 2.3.4, the Fritiof model [47, 48] is considered together with the Bertini intra-nuclear cascade to simulate hadronic showers (FTFP_BERT). The results for 2012 data from a dedicated sample with insignificant pile-up ($\mu \approx 0$) are presented in Fig. 21 and show good agreement between data and MC simulations without indicating a strong preference for one of the hadronic shower models. More results of the full systematic evaluation of the topo-cluster response to single charged hadron tracks, including for selected tracks from identified charged mesons and baryons, are available in Ref. [45].

### Effect of pile-up on topo-cluster observables

**Fig. 22:**
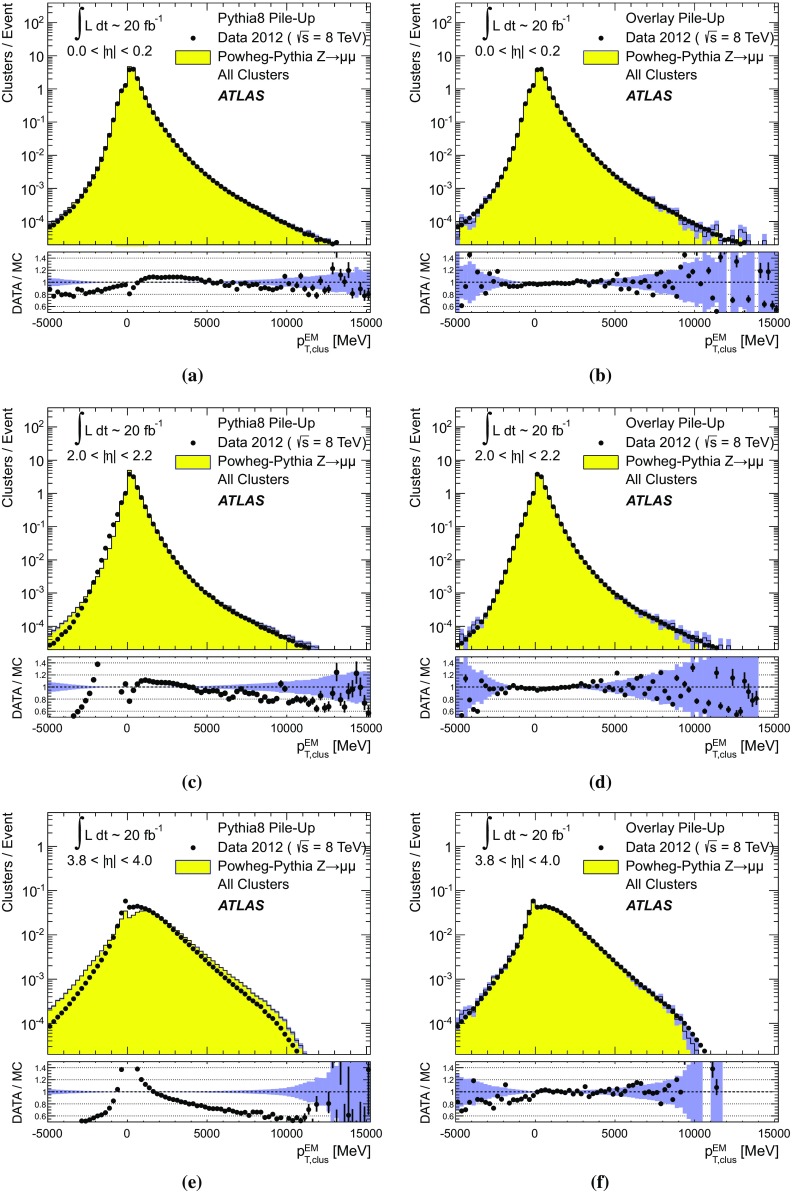
The distribution of the transverse momentum of topo-clusters reconstructed on the EM scale ($p_{\text {T},\text {clus}}^{{{\text {EM}}}}$) for an inclusive $Z \!\rightarrow \!\mu \mu$ event sample recorded in 2012. Data are compared to distributions from MC simulations (**a**, **c**, **e**) including fully simulated pile-up and (**b**, **d**, **f**) with pile-up overlaid from data for all topo-clusters within (**a**, **b**) $|\eta _{\text {clus}}|<0.2$, (**c**, **d**) $2.0<|\eta _{\text {clus}}|<2.2$ , (**e**, **f**) $3.8< |\eta _{\text {clus}}| < 4.0$. The ratio of the distribution from data to the one from MC simulation is evaluated bin-by-bin and shown below the respective distribution. The *shaded bands* indicate the statistical uncertainties from MC simulations for both the spectra and the ratios

The topo-cluster reconstruction performance is affected by in-time and out-of-time pile-up. While in-time pile-up is expected to usually increase the number of topo-clusters with increasing number of reconstructed vertices ($N_{\text {PV}}$), the out-of-time pile-up leads to cluster signal and shape modifications introduced by the calorimeter signal shaping functions described in Sect. 2.2.1.

The high density of very significant cell signals generated inside jets in the calorimeter increases the likelihood of low-energy pile-up signals to survive in the topo-cluster formation, according to the formation rules given in Sect. 3.1. Cell signals generated by the energy flow of relatively isolated particles entering the calorimeter outside jets or (stochastic) jet-like flow structures^14^ often have less significant neighbouring cells and thus contribute less often to topo-clusters. Consequently, the acceptance of the calorimeter for these particles, many of which are produced by pile-up, is lower than for particles in or around a jet.

In this section the modelling of the pile-up effects on the kinematics and moments used for the LCW calibration is compared to data for topo-clusters formed inside and outside jets for the conditions during 2012 running. The effect of pile-up on jets reconstructed from topo-clusters is discussed in Sect. 6.3, together with the stability of topo-cluster-based observables associated with the jet and its composition.

#### Event selection

The data used for the evaluation of the pile-up effects on topo-cluster kinematics and moments are collected from $Z \!\rightarrow \!\mu \mu$ events recorded in 2012. As indicated in Sect. 2.1, the corresponding sample is defined by a muon-based trigger. The additional event selection, applied to both data and the corresponding MC simulations, requires two muons with $p_{\text {T}}> 25\,{\text {GeV}}$ within $|\eta | < 2.4$ and an invariant mass $m_{\mu \mu }$ of the muon pair of $80\,{\text {GeV}}< m_{\mu \mu } < 100\,{\text {GeV}}$ for the inclusive sample. For the analysis of an exclusive sample with softer hadronic recoil against the $Z$ boson transverse momentum ($p_{\text {T}}^{Z}$), events with at least one jet reconstructed with the anti-$k_{t}$ algorithm and a distance parameter $R = 0.4$ and $p_{\text {T}}> 20\,{\text {GeV}}$ are removed. This sample is characterised by a final state dominated by in-time pile-up signal contributions, with only a small number of topo-clusters associated with the hadronic recoil.

Another exclusive sample for the analysis of topo-cluster features in jets is selected by requiring at least one anti-$k_{t}$ jet with $p_{\text {T}}> 20\,{\text {GeV}}$ in the event. Like in the selection applied to collect the exclusive sample without jets, all jets are fully calibrated and corrected, including a correction for pile-up (see Sect. 2.4). All inclusive and exclusive samples are thus characterised by their stability against pile-up.

#### Modelling of topo-cluster kinematics in events with pile-up

Detailed data/MC comparisons of topo-cluster kinematics yield significant differences between the measured and the modelled spectra. The transverse momentum spectra of topo-clusters reconstructed on the EM scale ($p_{\text {T},\text {clus}}^{{{\text {EM}}}}$) for the final state of an inclusive $Z \!\rightarrow \!\mu \mu$ sample, are shown in Figs. 22a and b for the central, in Figs. 22c and d for the end-cap, and in Figs. 22e and f for the forward detector region. The comparison between the $p_{\text {T},\text {clus}}^{{{\text {EM}}}}$ spectra from MC simulations with fully modelled pile-up and data in the various $\eta _{\text {clus}}$ ranges shows significant disagreements. Possible sources are an imperfect detector simulation or the modelling of the underlying soft physics processes in the MC generator.

Using the data overlay method described in Sect. 2.3.3 improves the data/MC comparison of the $p_{\text {T},\text {clus}}^{{{\text {EM}}}}$ spectra significantly, especially in the low-$p_{\text {T}}$ regime, where pile-up is expected to have a large effect. This improvement can be seen in Fig. 22b, d and f for the respective $\eta _{\text {clus}}$ ranges.

#### Transverse momentum flow in the presence of pile-up

**Fig. 23:**
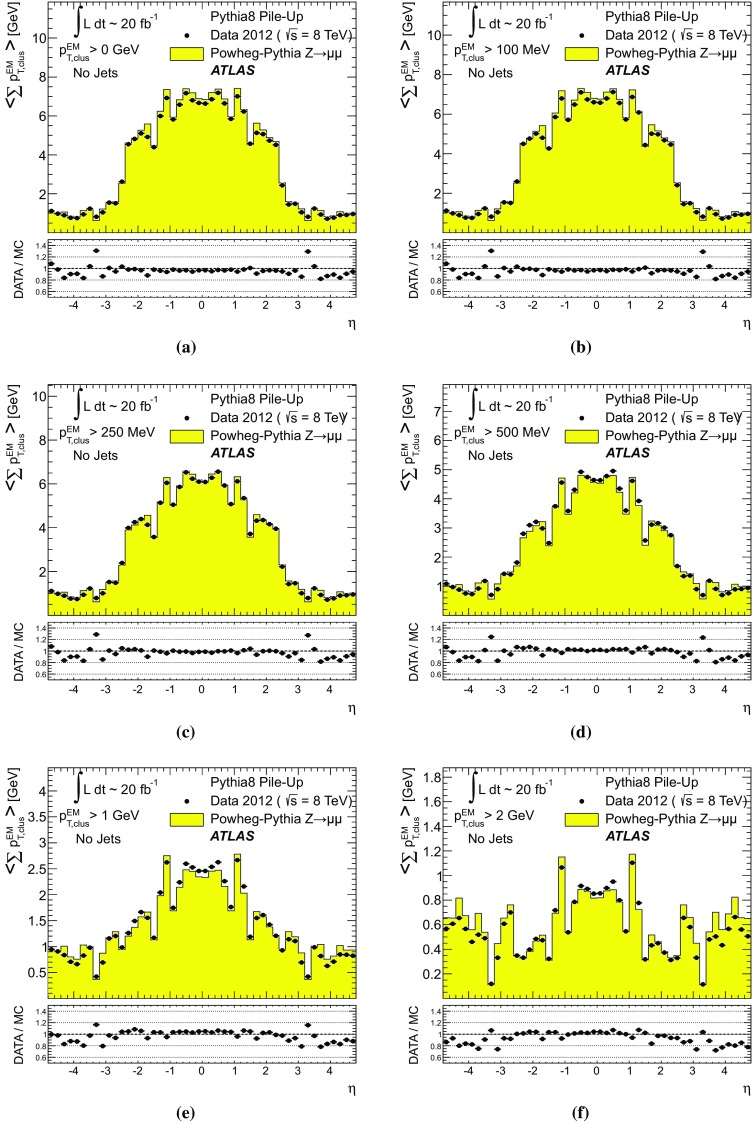
The average $\langle \Sigma p_{\text {T},\text {clus}}^{{{\text {EM}}}}\rangle$ of clusters at the EM scale, calculated as function of $\eta$ using Eq. (41), for clusters with **a** $p_{\text {T},\text {clus}}^{{{\text {EM}}}}> 0$, **b** $p_{\text {T},\text {clus}}^{{{\text {EM}}}}> 100\,\text {MeV}$, **c** $p_{\text {T},\text {clus}}^{{{\text {EM}}}}> 250\,\text {MeV}$, **d** $p_{\text {T},\text {clus}}^{{{\text {EM}}}}> 500\,\text {MeV}$, **e** $p_{\text {T},\text {clus}}^{{{\text {EM}}}}> 1\,{\text {GeV}}$, and **f** $p_{\text {T},\text {clus}}^{{{\text {EM}}}}> 2\,{\text {GeV}}$. Results are obtained from a 2012 $Z \!\rightarrow \!\mu \mu$  sample without jets with $p_{\text {T}}> 20\,{\text {GeV}}$ in data and MC simulation. The ratios of $\langle \Sigma p_{\text {T},\text {clus}}^{{{\text {EM}}}}\rangle (\eta )$ from data and MC simulations are shown below each plot

The transverse momentum flow in the $Z \!\rightarrow \!\mu \mu$ sample without jets with $p_{\text {T}}> 20\,{\text {GeV}}$ is reconstructed using the exclusive selection defined in Sect. 6.2.1. Topo-clusters are selected by $p_{\text {T},\text {clus}}^{{{\text {EM}}}}> p_{\text {T},\text {min}}$, where $p_{\text {T},\text {min}} \in \{ 0, 100\,\text {MeV}, 250\,\text {MeV}, 500\,\text {MeV}, 1\,{\text {GeV}}, 2\,{\text {GeV}} \}$. The flow is measured by the average total transverse momentum $\langle \Sigma p_{\text {T},\text {clus}}^{{{\text {EM}}}}\rangle$, carried by all or selected topo-clusters in any given direction $\eta _{k} \le \eta _{\text {clus}}< \eta _{k+1}$, and averaged over a given number of events $N_{\text {evts}}$:

41 $$\begin{aligned} \langle \Sigma p_{\text {T},\text {clus}}^{{{\text {EM}}}}\rangle (\eta _{\text {clus}}) = \dfrac{1}{N_{\text {evts}}} \sum _{i=1}^{N_{\text {evts}}}\left[ \sum _{\{j\,|\eta _{k}<\eta _{\text {clus},j}<\eta _{k+1}\}} p_{\text {T},\text {clus},j}^{{{\text {EM}}}}\right] _{i} . \end{aligned}$$

Here $\eta _{k}$ denotes the lower boundary of the *k*-th $\eta$-bin used to sum the transverse momentum of the selected topo-clusters in each event. Figure 23 shows $\langle \Sigma p_{\text {T},\text {clus}}^{{{\text {EM}}}}\rangle$ as a function of $\eta _{\text {clus}}$ for the various topo-cluster selections for this $Z \!\rightarrow \!\mu \mu$ data sample and the corresponding MC simulations.

**Fig. 24:**
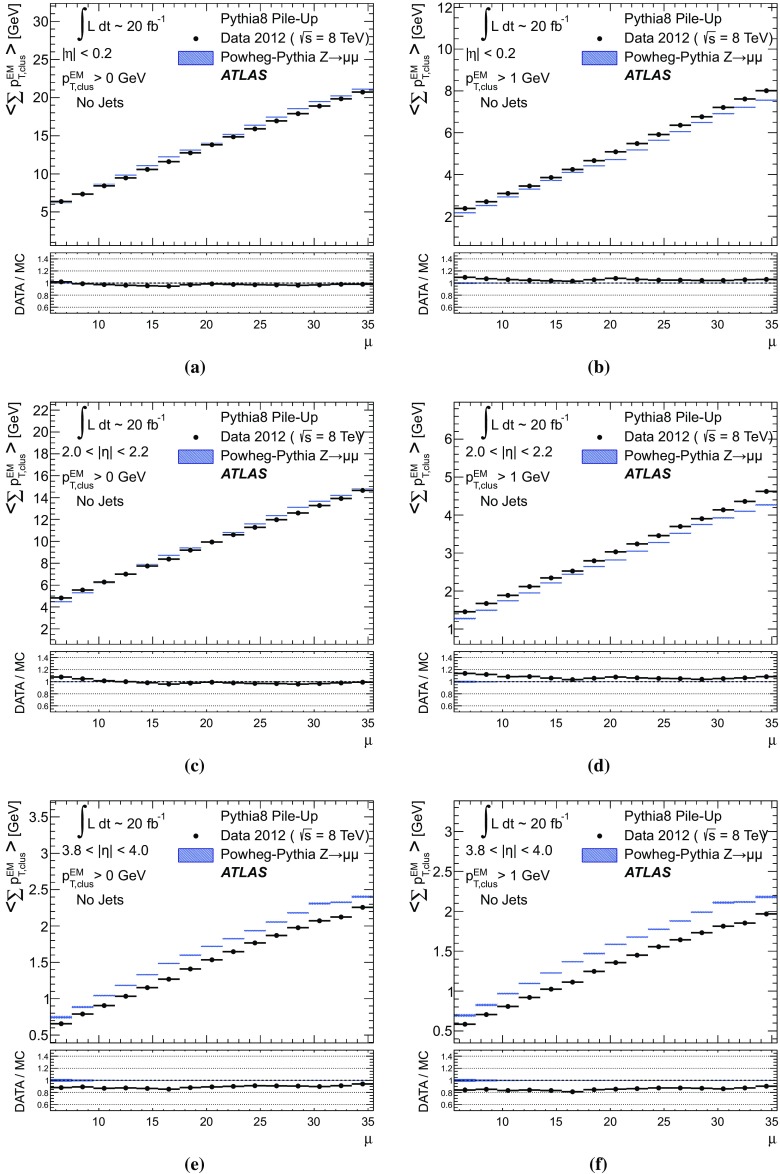
The average transverse momentum flow $\langle \Sigma p_{\text {T},\text {clus}}^{{{\text {EM}}}}\rangle$ evaluated as function of the pile-up activity measured by the number of proton–proton interactions per bunch crossing $\mu$, in several calorimeter regions. In **a**, **c**, and **e**, $\langle \Sigma p_{\text {T},\text {clus}}^{{{\text {EM}}}}\rangle (\mu )$ is shown in the central ($|\eta | < 0.2$), end-cap ($2.0<|\eta |<2.2$), and the forward ($3.8<|\eta |<4.0$) region, respectively, using topo-clusters with $p_{\text {T},\text {clus}}^{{{\text {EM}}}}> 0$. The corresponding results using topo-clusters with $p_{\text {T},\text {clus}}^{{{\text {EM}}}}> 1\,{\text {GeV}}$ are presented in **b**, **d**, and **f**. Results are obtained from a 2012 $Z \!\rightarrow \!\mu \mu$ sample without jets with $p_{\text {T}}> 20\,{\text {GeV}}$ in data and MC simulations. The narrow *shaded bands* around the results for MC simulations indicate statistical uncertainties, both for $\langle \Sigma p_{\text {T},\text {clus}}^{{{\text {EM}}}}\rangle (\mu )$ and the corresponding data-to-MC simulation ratios shown below each plot

The pile-up dependence of the average transverse momentum flow in various detector regions, as expressed by $\langle \Sigma p_{\text {T},\text {clus}}^{{{\text {EM}}}}\rangle (\mu )$, is shown in Fig. 24 for an inclusive ($p_{\text {T},\text {clus}}^{{{\text {EM}}}} > 0$) and a exclusive ($p_{\text {T},\text {clus}}^{{{\text {EM}}}} > 1\,{\text {GeV}}$) topo-cluster selection. The MC simulations predict the flow in the detector regions $|\eta | < 0.2$ and $2.0<|\eta |<2.2$ well, in particular for the more pile-up-sensitive cluster selection shown in Figs. 24a and c. Larger deviations are observed for these two regions with the exclusive selection in Figs. 24b and d. In the forward region, MC simulations predict higher $p_{\text {T}}$-flow for both topo-cluster selections, as can be seen in Figs. 24e and f. The slope of the $\langle \Sigma p_{\text {T},\text {clus}}^{{{\text {EM}}}}\rangle (\mu )$ dependence in this region is very similar for data and MC simulations.

The observations in Figs. 22, 23, 24 indicate that in the case of the fully simulated pile-up the simulation of the topo-cluster response to the underlying transverse energy flow outside jets suffers from MC simulation deficiencies. The use of overlaid pile-up from data, while not demonstrated here in all details, promises significant improvements for the modelling of the soft-event signals.

#### Topo-cluster multiplicity in the presence of pile-up

**Fig. 25:**
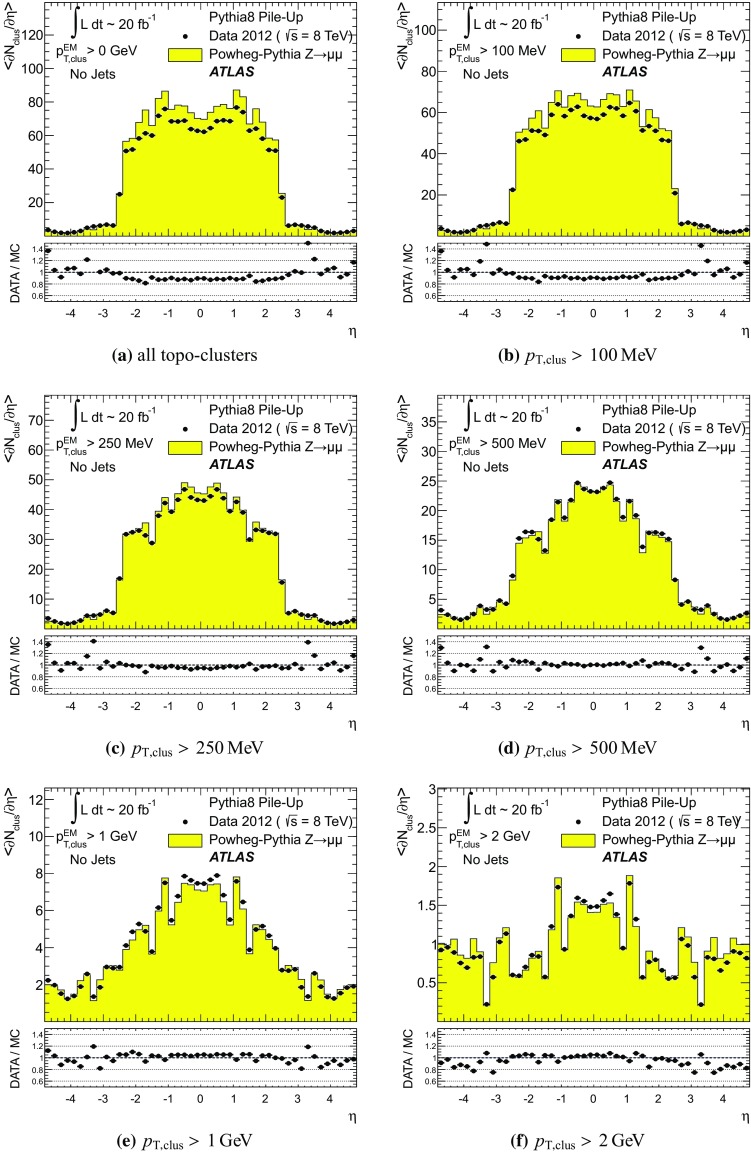
Average topo-cluster number density $\langle \partial N/\partial \eta \rangle$ as a function of $\eta _{\text {clus}}$, for clusters with $p_{\text {T},\text {clus}}^{{{\text {EM}}}}> p_{\text {T},\text {min}}$, for various $p_{\text {T},\text {min}}$ values. Results are obtained from a 2012 $Z \!\rightarrow \!\mu \mu$ sample without jets with $p_{\text {T}}> 20\,{\text {GeV}}$ in data and MC simulations. The corresponding data-to-MC simulation ratios are shown below each figure

**Fig. 26:**
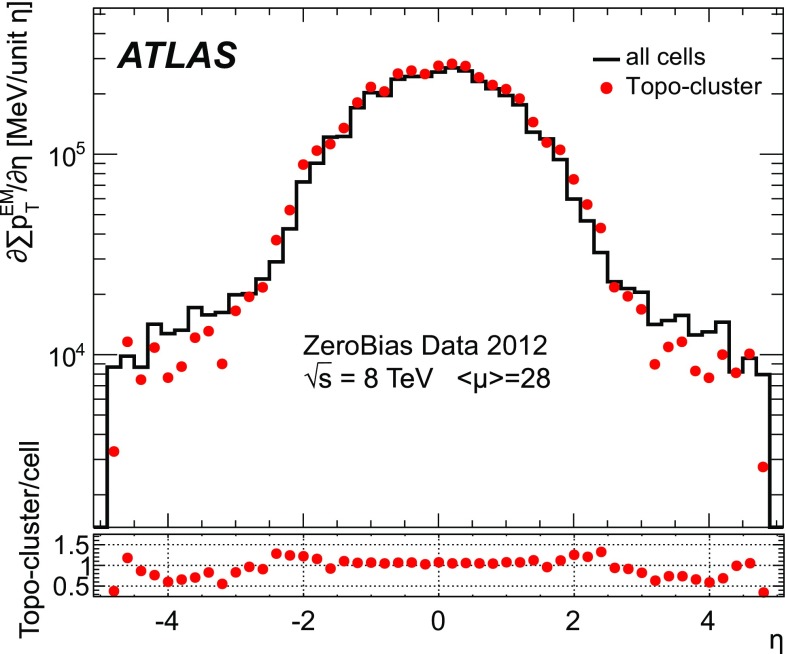
The reconstructed average transverse momentum flow on EM scale, measured with topo-clusters in bins of $\eta$ using $\langle \Sigma p_{\text {T},\text {clus}}^{{{\text {EM}}}}\rangle (\eta )$ in Eq. (41) and with all calorimeter cells in the same $\eta$-bins using ${\Sigma p_{\text {T},\text {cell}}^{{{\text {EM}}}}}(\eta )$ given in Eq. (42), in 2012 MB data

**Fig. 27:**
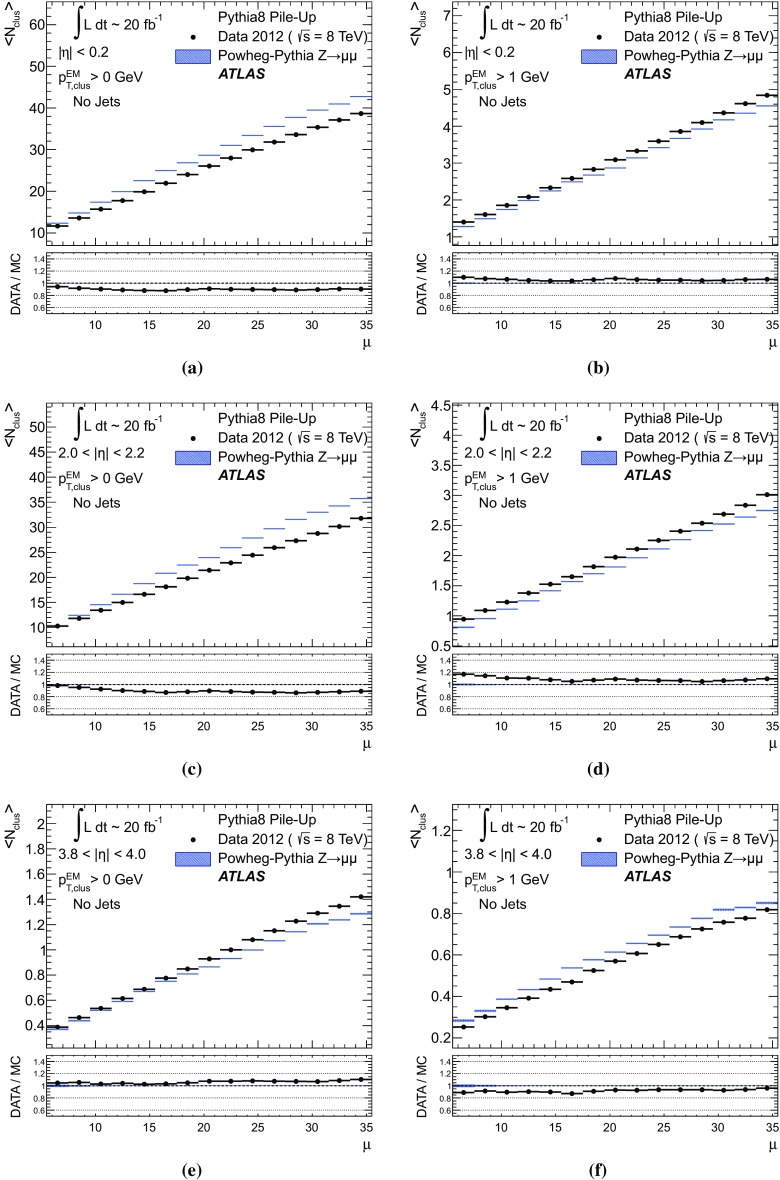
The dependence of the average number of positive-energy topo-clusters on the pile-up activity measured by the number of proton–proton collisions per bunch crossings $\mu$ in several regions of the detector is shown in **a**, **b** for $|\eta | < 0.2$, in **c**, **d** for $2.0<|\eta |<2.2$, and in **e**, **f** for $3.8<|\eta |<4.0$. Plots **a**, **c** and **e** show the results for counting all clusters with $p_{\text {T},\text {clus}}^{{{\text {EM}}}}> 0$, while **b**, **c** and **f** show the results for only counting clusters with $p_{\text {T},\text {clus}}^{{{\text {EM}}}}> 1\,{\text {GeV}}$. The corresponding ratio of data to MC simulations is shown below each plot. All results are obtained from a 2012 $Z \!\rightarrow \!\mu \mu$  sample without jets with $p_{\text {T}}> 20\,{\text {GeV}}$ in data and MC simulations. The narrow *shaded bands* indicate the statistical uncertainties associated with the results from MC simulations for the mean values and the ratios

The calorimeter signal occupancy in the exclusive $Z \!\rightarrow \!\mu \mu$ sample is determined using selected topo-clusters with $p_{\text {T},\text {clus}}^{{{\text {EM}}}}> p_{\text {T},\text {min}}$ and the $p_{\text {T},\text {min}}$ values used in Sect. 6.2.3. The relevant observable is the cluster number density, which is given by the number of topo-clusters per unit $\eta$ ($\partial N_{\text {clus}}/\partial \eta$). Figure 25 shows the average $\langle \partial N_{\text {clus}}/\partial \eta \rangle (\eta _{\text {clus}})$ for these topo-cluster selections, for data and MC simulations with fully simulated pile-up. The shape observed especially for the less restrictive selections with $p_{\text {T},\text {min}} \le 500\,\text {MeV}$ in Fig. 25a–d, reflects the variations of the calorimeter segmentation and the effect of sub-detector transition regions on the topo-cluster formation across the full ATLAS acceptance $|\eta _{\text {clus}}| < 4.9$. Generally, MC simulations describe the $p_{\text {T}}$-flow better than the number of clusters. This is expected as the description of the summed $p_{\text {T}}$-flow is constrained with more weight in the numerical fits for the ATLAS tunes than the particle number density.

The topo-cluster number density changes rapidly at $|\eta _{\text {clus}}|=2.5$. This is a consequence of the reduction of the calorimeter cell granularity by about a factor of four in terms of pseudorapidity and azimuth ($\Delta \eta \times \Delta \phi$), which reduces the number of potential topo-cluster seeds. The granularity change also introduces more signal overlap between individual particles in any given cell and thus less spatial resolution for the reconstruction of the corresponding energy flow due to this merging of particle signals. In addition, the larger cells increase the noise thresholds, as shown in Figs. 4b and c, which changes the calorimeter sensitivity. This change of sensitivity can be evaluated by comparing $\langle \Sigma p_{\text {T},\text {clus}}^{{{\text {EM}}}}\rangle$ with the corresponding quantity

42 $$\begin{aligned} \langle \Sigma p_{\text {T},\text {cell}}^{{{\text {EM}}}}\rangle (\eta _{\text {cell}}) = \dfrac{1}{N_{\text {evts}}}\sum _{i=1}^{N_{\text {evts}}}\left[ \sum _{\{j\,|\eta _{k}<\eta _{\text {cell},j}<\eta _{k+1}\}} p_{\text {T},\text {cell},j}^{{{\text {EM}}}}\right] _{i}, \end{aligned}$$

reconstructed from all calorimeter cell signals in each $\eta$ bin, similar to Eq. (41) for clusters. The cell-based $p_{\text {T}}$-flow expressed by $\langle \Sigma p_{\text {T},\text {cell}}^{{{\text {EM}}}}\rangle (\eta _{\text {cell}})$ is unbiased with respect to noise suppression as none is applied. Consequently, it is subject to larger fluctuations. Figure 26 shows this measurement for a 2012 MB data sample with pile-up close to the nominal $\mu = 30$ used for the noise thresholds (see Sect. 2.2.2). It indicates signal losses due to clustering up to about $50\,\%$ for $2.5< |\eta | < 4.5$, and some signal increase due to suppression of cells with $E < 0$, in particular in the end-cap region $1.5< |\eta | < 2.5$. All topo-clusters and calorimeter cell signals are accepted for this study.

The geometry effect yields the steep drop in topo-cluster number density at this boundary. Raising the transverse momentum threshold for accepted topo-clusters increasingly mitigates the geometrical and noise effects on the cluster number density. The data/MC comparison shows larger deficiencies for more inclusive topo-cluster selections, which capture more signals from pile-up. It improves as the $p_{\text {T},\text {min}}$ threshold increases, when the selections are dominated by clusters that are generated by harder emissions than those due to pile-up.

The dependence of the average number of topo-clusters in a given calorimeter region on the pile-up activity, expressed in terms of $\mu$, is shown for clusters with $p_{\text {T},\text {clus}}^{{{\text {EM}}}}> 0$ and $p_{\text {T},\text {clus}}^{{{\text {EM}}}}> 1\,{\text {GeV}}$ in Fig. 27. Applying the (inclusive) $p_{\text {T},\text {clus}}^{{{\text {EM}}}}> 0$ selection yields more topo-clusters in MC simulations than in data in the selected central ($|\eta |<0.2$) and end-cap ($2.0<|\eta |<2.2$) regions, with the difference rising with increasing $\mu$ in Figs. 27a and c. In the forward region the number of topo-clusters in MC simulations is closer to the number in data for low $\mu$, but tends to be lower than data at higher $\mu$, as seen in Fig. 27e.

These qualitative differences between the observations for the central and end-cap regions and the forward region can arise from the modelling of soft physics, which is tuned with reconstructed charged tracks in the detector region $|\eta | < 2.5$ but is not experimentally constrained in the forward region. In addition, imperfections in the description of the inactive material in front of the calorimeter in the detector simulation can change the acceptance for low-energy particles significantly in different ways in the various $\eta$-regions. Also, mismodelling in the simulation of the (mostly hadronic) lateral and longitudinal shower spreads in the calorimeters, as e.g. documented in Refs. [49, 50], can lead to different topo-cluster splitting behaviour in the different calorimeter regions. In particular the increased signal overlap between particles in the forward region is suspected to introduce a higher sensitivity of the cluster splitting to the detector simulation.

As can be seen in Figs. 27b and f, counting only topo-clusters with $p_{\text {T},\text {clus}}^{{{\text {EM}}}}> 1\,{\text {GeV}}$ introduces a more similar slope in the cluster number density as a function of $\mu$. The qualitative behaviour of $\langle \partial N_{\text {clus}}/\partial \eta \rangle (\eta _{\text {clus}})$ in the various detector regions is different than for the more inclusive topo-cluster selection, with MC simulation predicting fewer clusters in the central and end-cap regions shown in Figs. 27b and d. In the forward region, data shows overall fewer clusters than MC simulation, as can be seen in Fig. 27f, with larger differences at any given $\mu$, but a very similar number of additional clusters per additional proton–proton interaction.

#### Modelling of the topo-cluster depth location in the presence of pile-up

**Fig. 28:**
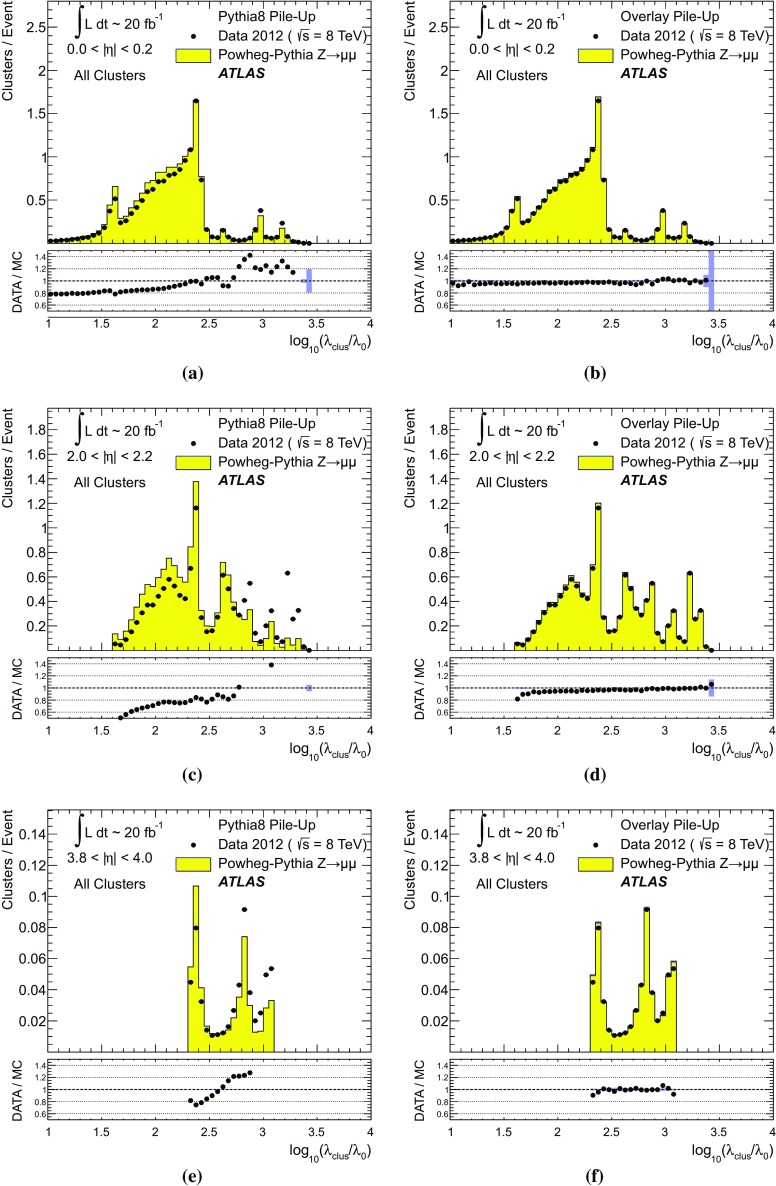
The distribution of the topo-cluster depth location, measured in terms of $\log _{10}(\lambda _{\text {clus}}/\lambda _{0})$, for clusters in various bins of $\eta _{\text {clus}}$ for an inclusive $Z \!\rightarrow \!\mu \mu$ event sample recorded in 2012. Data is compared to distributions from MC simulations including fully simulated pile-up for all topo-clusters within **a** $|\eta _{\text {clus}}| < 0.2$, **c** $2.0< |\eta _{\text {clus}}| < 2.2$, and **e** $3.8< |\eta _{\text {clus}}| < 4.0$. The corresponding distributions for MC simulations with pile-up from data overlaid are depicted in **b**, **d**, and **f**. The ratios of the distributions for data and MC simulations are shown below the respective distributions. The *shaded bands* indicate the statistical uncertainties for MC simulations

**Fig. 29:**
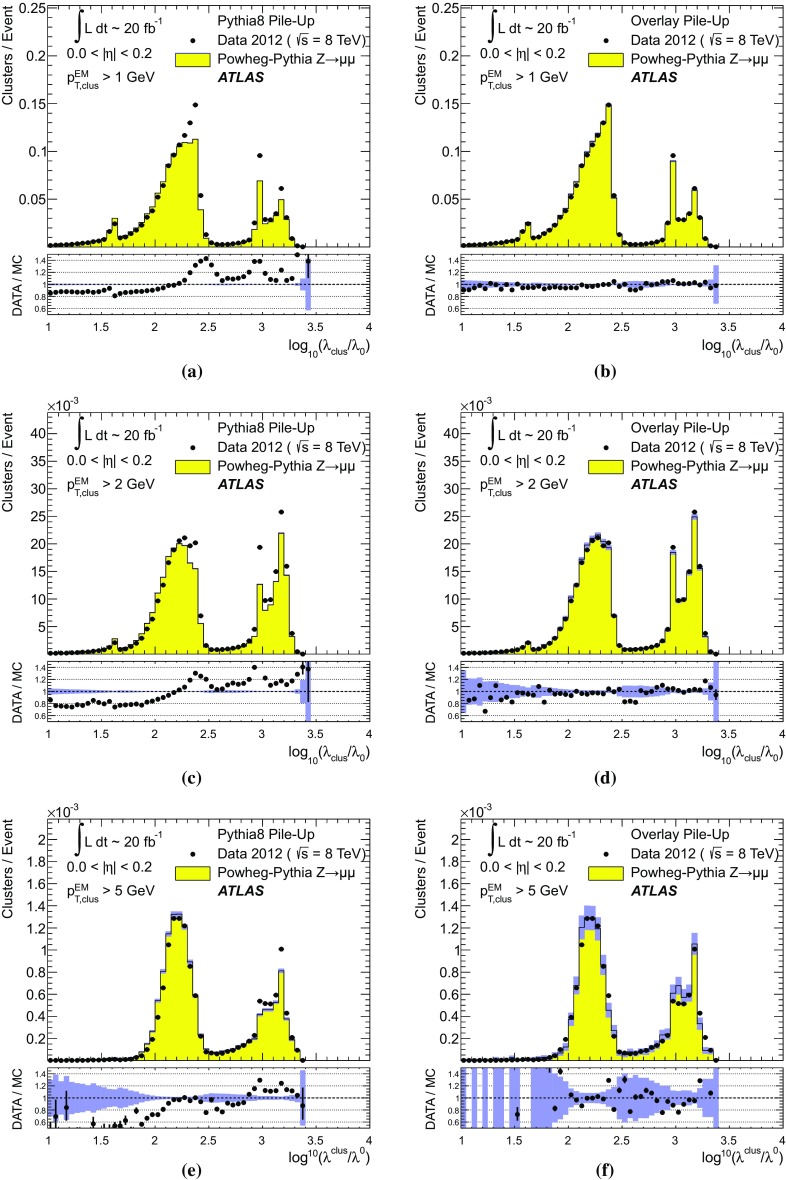
The distribution of the topo-cluster depth location, measured in terms of $\log _{10}(\lambda _{\text {clus}}/\lambda _{0})$, for selected topo-clusters within $|\eta _{\text {clus}}| < 0.2$ and with a transverse momentum $p_{\text {T},\text {clus}}^{{{\text {EM}}}}$, evaluated on the EM scale, larger than various thresholds. Results are shown for an inclusive $Z \!\rightarrow \!\mu \mu$ event sample recorded in 2012. Data are compared to distributions from MC simulations including fully simulated pile-up for all topo-clusters with **a** $p_{\text {T},\text {clus}}^{{{\text {EM}}}}> 1\,{\text {GeV}}$, **c** $p_{\text {T},\text {clus}}^{{{\text {EM}}}}> 2\,{\text {GeV}}$, and **e** $p_{\text {T},\text {clus}}^{{{\text {EM}}}}> 5\,{\text {GeV}}$. The corresponding distributions for MC simulations with pile-up from data overlaid are depicted in **b**, **d**, and **f**. The *shaded bands* indicate the statistical uncertainties for the distributions obtained from MC simulations and the corresponding uncertainties in the ratio plots

Pile-up is expected to affect cluster moments as well as the overall cluster kinematics. Its diffuse energy emission can not only produce additional topo-clusters, but also change the centre of gravity, the barycentre, and other cluster shapes. In some cases, pile-up can actually increase the cluster splitting, as additional local signal maxima can be inserted into a topo-cluster by pile-up. In addition, the increased cell noise can produce additional signal minima in groups of previously connected cells in the topo-cluster. This last effect can be more important for topo-clusters in jets and is discussed in Sect. 6.3. The topo-cluster depth location $\lambda _{\text {clus}}$ discussed here serves as an example for the quality of modelling cluster moments in the presence of pile-up. Other moments are investigated in the context of jets.

The modelling of $\lambda _{\text {clus}}$ in the calorimeter is compared to data in Fig. 28 for the inclusive $Z \!\rightarrow \!\mu \mu$ sample in the same bins of $\eta _{\text {clus}}$ used for the study of $p_{\text {T},\text {clus}}$ in Fig. 22. The fully simulated events with pile-up from the minimum-bias simulations show significant differences from the data, while the MC simulations overlaid with pile-up from data show good agreement with respect to all features of these distributions. The complex structure of the distributions reflects the longitudinal calorimeter segmentation in the various regions defined by $\eta _{\text {clus}}$. For example, the forward direction $3.8< |\eta _{\text {clus}}| < 4.0$ is covered by the FCAL, which has three coarse and deep longitudinal segments (approximately $2.5/3.5/3.5\,\lambda _{\text {nucl}}$). This structure generates topo-clusters preferably in the depth centre of each module, as can be seen in Figs. 28e and f. These distributions are dominated by low-energy clusters associated with pile-up interactions such that the improvement seen by using data overlay is expected.

Similarly to the studies of the kinematic and flow properties of topo-clusters discussed in Sects. 6.2.2 and 6.2.3, more exclusive topo-cluster selections are also investigated. Figure 29 shows data/MC comparisons of the $\lambda _{\text {clus}}$ distributions for clusters within $|\eta _{\text {clus}}| < 0.2$ for $p_{\text {T},\text {clus}}^{{{\text {EM}}}}> p_{\text {T},\text {min}}$ with $p_{\text {T},\text {min}} \in \{ 1, 2, 5 \}\,{\text {GeV}}$, for MC simulations with fully simulated pile-up and for MC simulations with pile-up from data overlaid. The MC simulation with overlaid pile-up agrees better with data than the one with fully simulated pile-up, particularly in the case of the least restrictive $p_{\text {T},\text {min}} = 1\,{\text {GeV}}$ topo-cluster selection.

### Topo-clusters in jets

Jets are important in many analyses at the LHC. Therefore, the performance of the simulation of their constituents is important, in particular for analyses employing jet substructure techniques or relying on the jet mass. In order to study the topo-cluster features in jets and the jet topo-cluster composition, exclusive jet samples are extracted from data and MC simulation using the $Z \!\rightarrow \!\mu \mu$ and jet selection described in Sect. 6.2.1. As the jets are globally corrected for pile-up [16], they form a stable kinematic reference for the evaluation of pile-up effects on the topo-clusters used to reconstruct them. Jets include only topo-clusters with $E > 0$, as required by the kinematic recombination.

The full evaluation of the reconstruction performance for jets formed with topo-clusters on both EM and LCW scale is presented in Refs. [16, 38]. The evaluation of the jet energy resolution can be found in Ref. [51].

#### Jet energy scale and topo-cluster-based response in pile-up

**Fig. 30:**
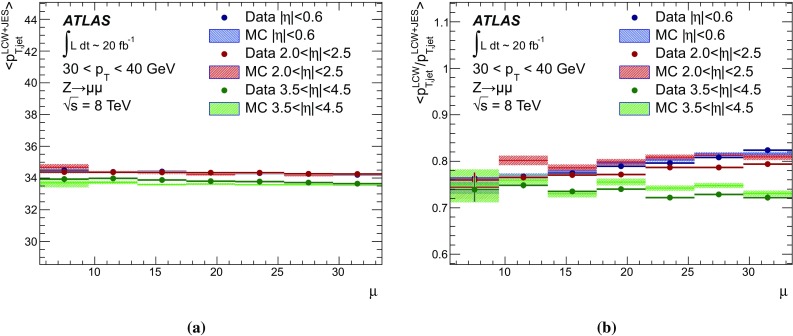
In **a**, the fully calibrated and corrected jet $p_{\text {T}}$ response measured by $p_{\text {T},\text {jet}}^{{\,{\text {LCW}}+\text {JES}}}$ is shown as a function of the pile-up activity measured by $\mu$, in three different detector regions for $Z \!\rightarrow \!\mu \mu$ events with one anti-$k_{t}$ jet with $R = 0.4$ with $30\,{\text {GeV}}< p_{\text {T},\text {jet}}^{{\,{\text {LCW}}+\text {JES}}}< 40\,{\text {GeV}}$, for 2012 data and MC simulations with fully simulated pile-up. The $\mu$ dependence of the uncorrected jet $p_{\text {T}}$ response is shown in **b**. It is measured in terms of its ratio to the fully calbrated jet response, $p_{\text {T},\text {jet}}^{{\,{\text {LCW}}}}/p_{\text {T},\text {jet}}^{{\,{\text {LCW}}+\text {JES}}}(\mu )$, for the same events and in the same detector regions. The *shaded bands* shown for the results from MC simulations indicate statistical uncertainties

As mentioned above, the fully calibrated four-momentum $\text {P}_{\text {jet}}$ of jets reconstructed from topo-clusters is corrected for pile-up effects. Therefore, the corresponding transverse momentum $p_{\text {T},\text {jet}}$ provides a stable signal for event selections and the kinematics of the true particle flow. The basic jet four-momentum is reconstructed on two different scales, the EM scale and the LCW scale using locally calibrated topo-clusters with $E > 0$:

43 $$\begin{aligned} \text {P}_{\text {jet}}^{\,{{\text {EM}}}}= & {} \sum _{i=1}^{N_{\text {clus}}^{\text {jet}}} \text {P}_{\text {clus}}^{\,{{\text {EM}}}} \end{aligned}$$

44 $$\begin{aligned} \text {P}_{\text {jet}}^{\,{{\text {LCW}}}}= & {} \sum _{i=1}^{N_{\text {clus}}^{\text {jet}}} \text {P}_{\text {clus}}^{\,{{\text {LCW}}}} \end{aligned}$$

The sum runs over the number $N_{\text {clus}}^{\text {jet}}$ of topo-clusters in a given jet. Both $\text {P}_{\text {jet}}^{\,{{\text {EM}}}}$ and $\text {P}_{\text {jet}}^{\,{{\text {LCW}}}}$ are not corrected further. The corresponding $p_{\text {T}}$ responses $p_{\text {T},\text {jet}}^{{\,{\text {EM}}}}$ and $p_{\text {T},\text {jet}}^{{\,{\text {LCW}}}}$ are therefore affected by pile-up. A full jet energy scale (JES) calibration is applied to both scales, yielding $\text {P}_{\text {jet}}^{{\,{\text {EM}}+\text {JES}}}$ and $\text {P}_{\text {jet}}^{{\,{\text {LCW}}+\text {JES}}}$, respectively. This JES calibration includes pile-up corrections, response calibration, direction corrections and refinements from *in situ* transverse momentum balances, similar to those outlined for 2011 data in Ref. [16]. The respective fully calibrated transverse momentum is then $p_{\text {T},\text {jet}}^{{\,{\text {EM}}+\text {JES}}}$ and $p_{\text {T},\text {jet}}^{{\,{\text {LCW}}+\text {JES}}}$.

**Fig. 31:**
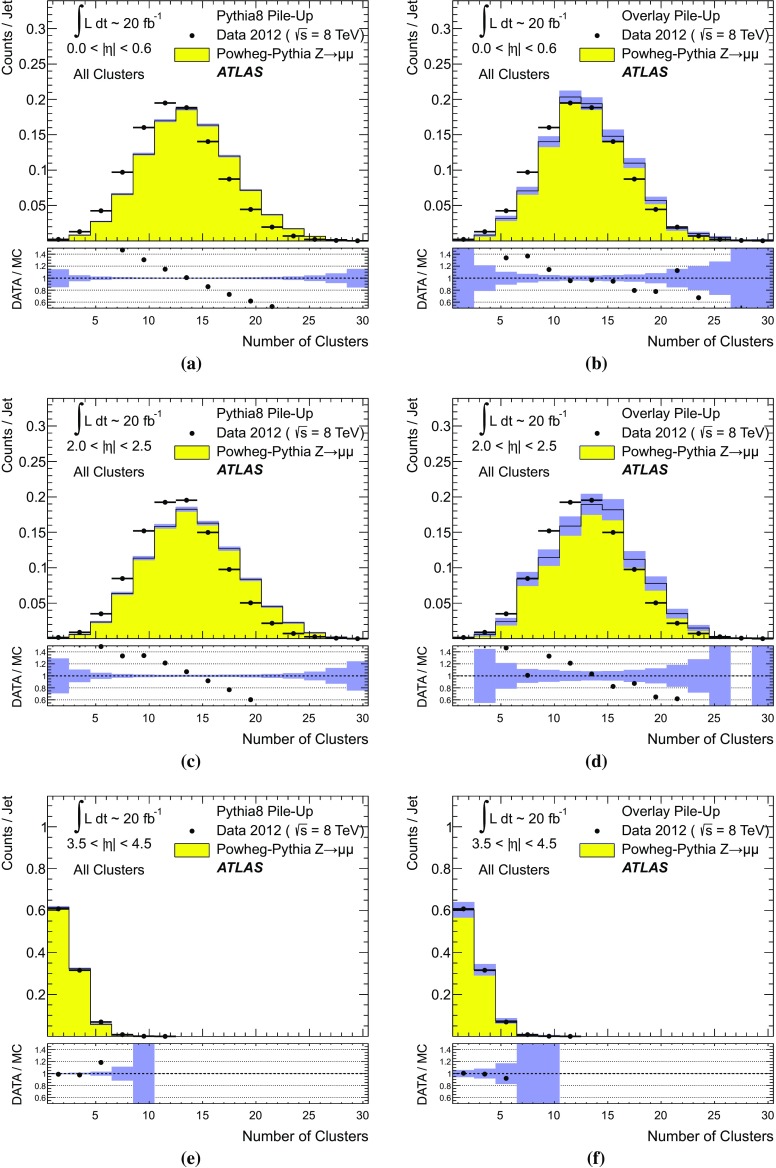
The distribution of the number of topo-clusters inside anti-$k_{t}$ jets formed with $R = 0.4$ in the (**a**, **b**) central ($|\eta |<0.6$), the (**c**, **d**) end-cap ($2.0<|\eta |<2.5$), and the (**e**, **f**) forward detector region ($3.5<|\eta |<4.5$) of ATLAS. Shown are results from the analysis of $Z \!\rightarrow \!\mu \mu$ events with at least one jet with $30\,{\text {GeV}}< p_{\text {T}}< 40\,{\text {GeV}}$ in 2012 data and MC simulations with fully simulated pile-up in **a**, **c** and **e**, and with pile-up from data overlaid in **b**, **d** and **f**. The ratios of results for data and MC simulations are shown below the distributions. The *shaded bands* show the statistical uncertainties for the distributions obtained from MC simulations and the corresponding uncertainty bands in the ratio plots

Figure 30 shows the pile-up dependence of the fully calibrated $p_{\text {T},\text {jet}}^{{\,{\text {LCW}}+\text {JES}}}$ and the uncorrected $p_{\text {T},\text {jet}}^{{\,{\text {LCW}}}}$ on the pile-up activity in the event, measured by $\mu$. Results are obtained from a $Z \!\rightarrow \!\mu \mu$ sample of events with one jet with $30\,{\text {GeV}}< p_{\text {T},\text {jet}}^{{\,{\text {LCW}}+\text {JES}}}< 40\,{\text {GeV}}$ in data and MC simulations. While Fig. 30a shows the stability of the corrected jet $p_{\text {T}}$ scale, Fig. 30b indicates the different sensitivities of the uncorrected response to pile-up in the various detector regions. The different shapes seen in this figure are mostly related to the calorimeter granularity and the specific shaping functions in the different LAr calorimeters. While the general expectation that every pile-up interaction adds energy to the jet is indicated in the rise of $p_{\text {T},\text {jet}}^{{\,{\text {LCW}}}}/p_{\text {T},\text {jet}}^{{\,{\text {LCW}}+\text {JES}}}$ with increasing $\mu$, the dependence of this ratio on $\mu$ is less pronounced for jets with $3.5< |\eta _{\text {jet}}| < 4.5$ in the FCAL calorimeter. This observation is related to the much coarser calorimeter geometry in this region, in addition to the different (faster) shaping function in the FCAL, yielding a better average online in-time pile-up suppression by the out-of-time pile-up signal history in 2012 running conditions ($50\,{\text {ns}}$ bunch crossings).

#### Topo-cluster multiplicity in jets

Figure 31 shows the distributions of the number of topo-clusters ($N_{\text {clus}}^{\text {jet}}$) in central, end-cap and forward jets. Distributions are shown using fully simulated pile-up and using data overlay. The discrepancies between MC simulations and data, while slightly reduced in the simulations employing the pile-up overlaid from data, generally persist.

**Fig. 32:**
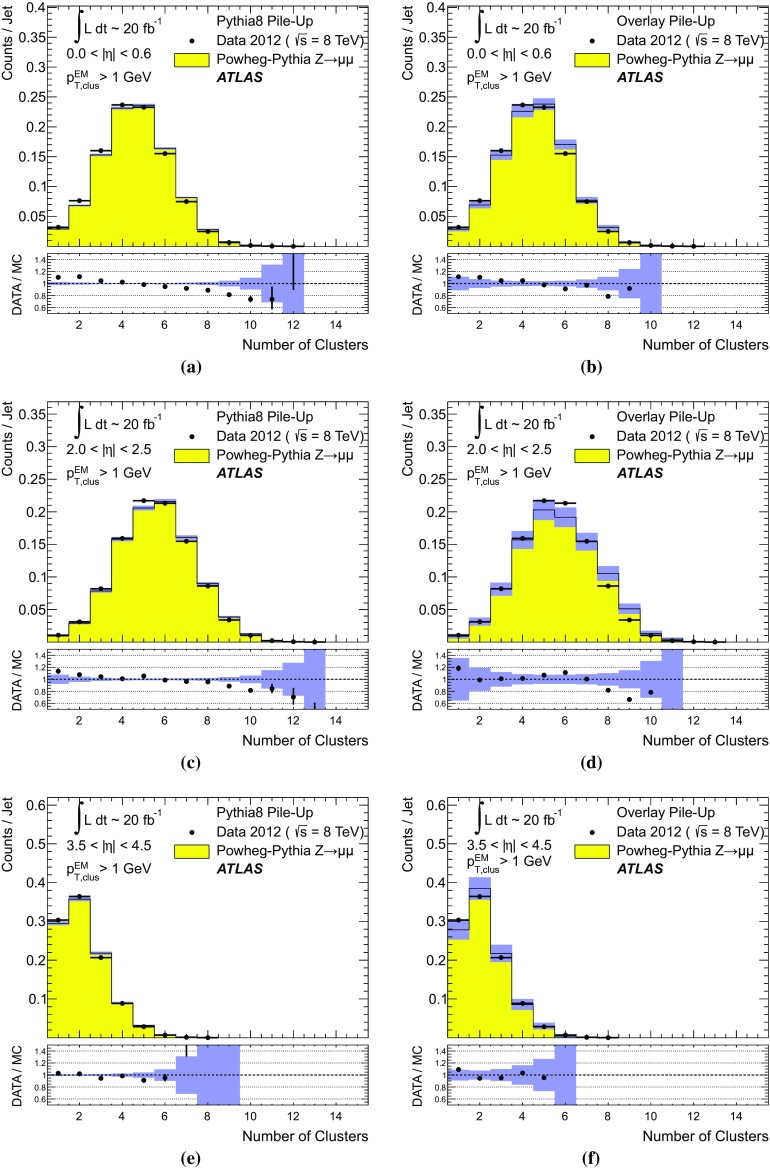
The distribution of the number of topo-clusters with $p_{\text {T},\text {clus}}^{{{\text {EM}}}}> 1\,{\text {GeV}}$ inside anti-$k_{t}$ jets with $R = 0.4$ in the (**a**, **b**) central ($|\eta |<0.6$), the (**c**, **d**) end-cap ($2.0<|\eta |<2.5$), and the (**e**, **f**) forward detector region ($3.5<|\eta |<4.5$) of ATLAS. Shown are results from the analysis of $Z \!\rightarrow \!\mu \mu$ events with at least one jet with $30\,{\text {GeV}}< p_{\text {T}}< 40\,{\text {GeV}}$ in 2012 data and MC simulations with fully simulated pile-up in **a**, **c** and **e**, and with pile-up from data overlaid in **b**, **d** and **f**. The data-to-MC simulation ratios are shown below the respective distributions. The *shaded bands* indicate statistical uncertainties for the distributions from MC simulations and the corresponding statistical uncertainty bands for the ratios

The data/MC comparisons of the cluster multiplicity distributions counting only topo-clusters with $p_{\text {T},\text {clus}}^{{{\text {EM}}}}> 1\,{\text {GeV}}$ for the same $Z \!\rightarrow \!\mu \mu$ data and MC simulations are shown in Fig. 32. This comparison is significantly improved with respect to Fig. 31, indicating that the number of low-energy topo-clusters in jets is poorly simulated. The comparison of data to MC simulations with fully simulated pile-up and with pile-up overlaid from data for the more inclusive cluster multiplicities in Fig. 31 indicates that pile-up is likely not the main source for the deficiencies in the MC simulation, as the comparison does not improve significantly when pile-up is taken from the data. This observation, together with the insensitivity of the data/MC comparison of the multiplicity of harder topo-clusters to the choice of pile-up modelling in MC simulations shown in Fig. 32, suggests that the deficiencies in the simulation of the low-energy topo-cluster multiplicity arise from imperfections in the detector model, response or tuning of the parton shower and other sources of soft emissions, including multiple parton interactions in the underlying event, rather than from the modelling of pile-up or electronic noise. Further investigations concerning the distribution of the topo-cluster location in jets confirm this interpretation and are presented in Sect. 6.3.3.

**Fig. 33:**
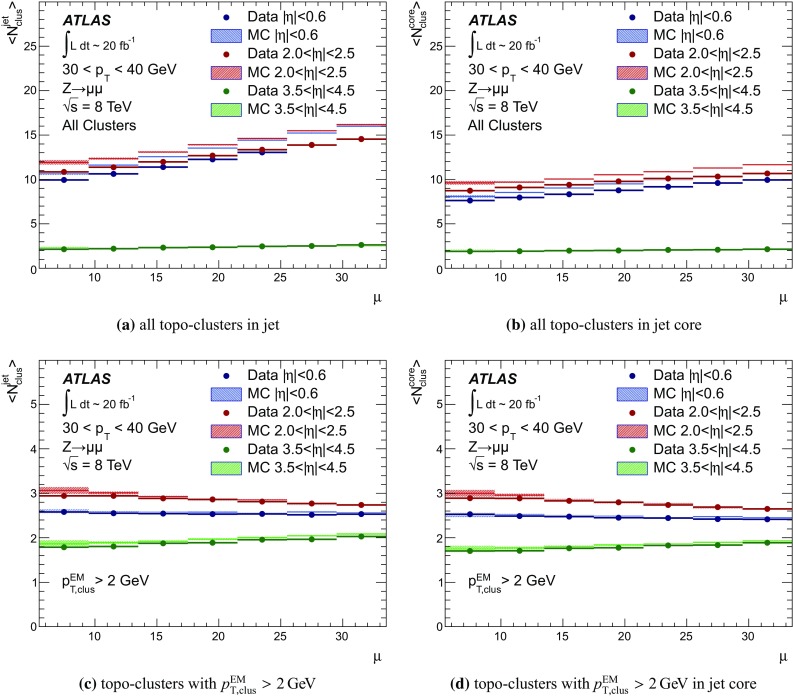
The average number of topo-clusters $\langle N_{\text {clus}}^{\text {jet}}\rangle$ in anti-$k_{t}$ jets reconstructed with $R = 0.4$ within $30\,{\text {GeV}}< p_{\text {T},\text {jet}}^{{\,{\text {LCW}}+\text {JES}}}< 40\,{\text {GeV}}$ as a function of $\mu$, in $Z \!\rightarrow \!\mu \mu$ events in 2012 data and MC simulations (**a**). The pile-up dependence of the average number of topo-clusters $\langle N_{\text {clus}}^{\text {core}}\rangle$ in the core of the jet, defined by the distance to jet axis $\Delta R < 0.3$, is shown in **b**. Selecting topo-clusters by $p_{\text {T},\text {clus}}^{{{\text {EM}}}}> 2\,{\text {GeV}}$ inside jets and in the core of the jet yields the $\mu$ dependencies shown in **c** and **d**. The *shaded bands* shown for the results obtained from MC simulations indicate statistical uncertainties

The dependence of the number of clusters $N_{\text {clus}}^{\text {jet}}$ forming the anti-$k_{t}$ jets of size $R = 0.4$ and with $30\,{\text {GeV}}< p_{\text {T},\text {jet}}^{{\,{\text {LCW}}+\text {JES}}}< 40\,{\text {GeV}}$ as a function of the pile-up activity, measured by $\mu$, is shown in Fig. 33. As indicated in Fig. 33a, $N_{\text {clus}}^{\text {jet}}$ rises approximately linearly with increasing $\mu$ in the central and end-cap detector regions. The gradient of this rise is much smaller in the forward region, where the coarser read-out geometry and the signal shaping effects already discussed in Sect. 6.3.1 in the context of Fig. 30b lead to merging and suppression of pile-up signals. Figure 33a also confirms the already mentioned deficiencies in the MC simulation of the absolute values of the most inclusive $\langle N_{\text {clus}}^{\text {jet}}\rangle$ in any given $\mu$ range, except for the forward detector region. The slope of $\langle N_{\text {clus}}^{\text {jet}}\rangle (\mu )$, on the other hand, compares well with data.

The number of topo-clusters in the core of the jet ($N_{\text {clus}}^{\text {core}}$) is defined by counting the clusters at distances $\Delta R < 0.3$ around the jet axis. Figure 33b shows a residual dependence of the average $\langle N_{\text {clus}}^{\text {core}}\rangle$ on $\mu$ in the central and end-cap regions, with significant differences between data and the predictions from MC simulations. The figure shows good data/MC agreement for $\langle N_{\text {clus}}^{\text {core}}\rangle$ in the forward region. Comparing $\langle N_{\text {clus}}^{\text {jet}}\rangle (\mu )$ in Fig. 33a with $\langle N_{\text {clus}}^{\text {core}}\rangle (\mu )$ in Fig. 33b shows a steeper slope for $\langle N_{\text {clus}}^{\text {jet}}\rangle (\mu )$ than for $\langle N_{\text {clus}}^{\text {core}}\rangle (\mu )$ in the central and end-cap calorimeter regions. Pile-up interactions add more topo-clusters at the margin of the jet than in the core. For forward jets, $\langle N_{\text {clus}}^{\text {jet}}\rangle$ rises only slightly with increasing $\mu$, while $\langle N_{\text {clus}}^{\text {core}}\rangle$ shows no observable dependency on pile-up.

Calculating $N_{\text {clus}}^{\text {jet}}$ and $N_{\text {clus}}^{\text {core}}$ with only considering topo-clusters with $p_{\text {T},\text {clus}}^{{{\text {EM}}}}> 2\,{\text {GeV}}$ yields the result for the pile-up dependence of $\langle N_{\text {clus}}^{\text {jet}}\rangle$ and $\langle N_{\text {clus}}^{\text {core}}\rangle$ displayed in Figs. 33c and d, respectively. While both $\langle N_{\text {clus}}^{\text {jet}}\rangle$ and $\langle N_{\text {clus}}^{\text {core}}\rangle$ are nearly independent of $\mu$ in the central detector region, they show more complex dependencies on the pile-up activity in the end-cap region. The loss of hard topo-clusters in both the overall jet and in its core with increasing $\mu$ reflects additional cluster splitting induced by the diffuse energy flow from pile-up in the end-cap calorimeters. The observations in both the central and the end-cap regions are well described by MC simulations.

A good quality of the MC predictions is also achieved when comparing the number of hard topo-clusters above the $p_{\text {T},\text {clus}}^{{{\text {EM}}}}$ threshold in forward jets. This number shows only a small increase with increasing $\mu$, as shown in Fig. 33c. This is due to the fact that the cluster splitting behaviour does not change with increasing pile-up in the coarse granularity of the FCAL. In this module, the residual signal contribution from pile-up shifts a small number of additional clusters above the $2\,{\text {GeV}}$ threshold, yielding an increase of about $10\,\%$ for both $\langle N_{\text {clus}}^{\text {jet}}\rangle$ and $\langle N_{\text {clus}}^{\text {core}}\rangle$ for $\mu < 10$ to $\mu > 30$. A comparison of $\langle N_{\text {clus}}^{\text {jet}}\rangle (\mu )$ with and without the $p_{\text {T},\text {clus}}^{{{\text {EM}}}}> 2\,{\text {GeV}}$ selection shows that the cut occasionally removes a topo-cluster from a forward jet such that $\langle N_{\text {clus}}^{\text {jet}}\rangle$ is reduced by not more than $15\,\%$ for any given $\mu$. The selection affects $\langle N_{\text {clus}}^{\text {core}}\rangle (\mu )$ in a different way. While $\langle N_{\text {clus}}^{\text {core}}\rangle (\mu ) \approx {{\mathrm{const}}}$ without the cut, the average number of topo-clusters in the jet core passing the $p_{\text {T},\text {clus}}^{{{\text {EM}}}}$ selection is smaller by approximately $15\,\%$ in the region of lower pile-up activity, where $\langle N_{\text {clus}}^{\text {jet}}\rangle (\mu< 10) \approx \langle N_{\text {clus}}^{\text {core}}\rangle (\mu < 10)$ both with and without the selection. It is only about $5\,\%$ smaller for higher pile-up, where $\langle N_{\text {clus}}^{\text {jet}}\rangle (\mu> 30)> \langle N_{\text {clus}}^{\text {core}}\rangle (\mu > 30)$ independent of the cut, as can be seen by comparing Fig. 33b with 33d for forward jets.

#### Topo-cluster location in jets

**Fig. 34:**
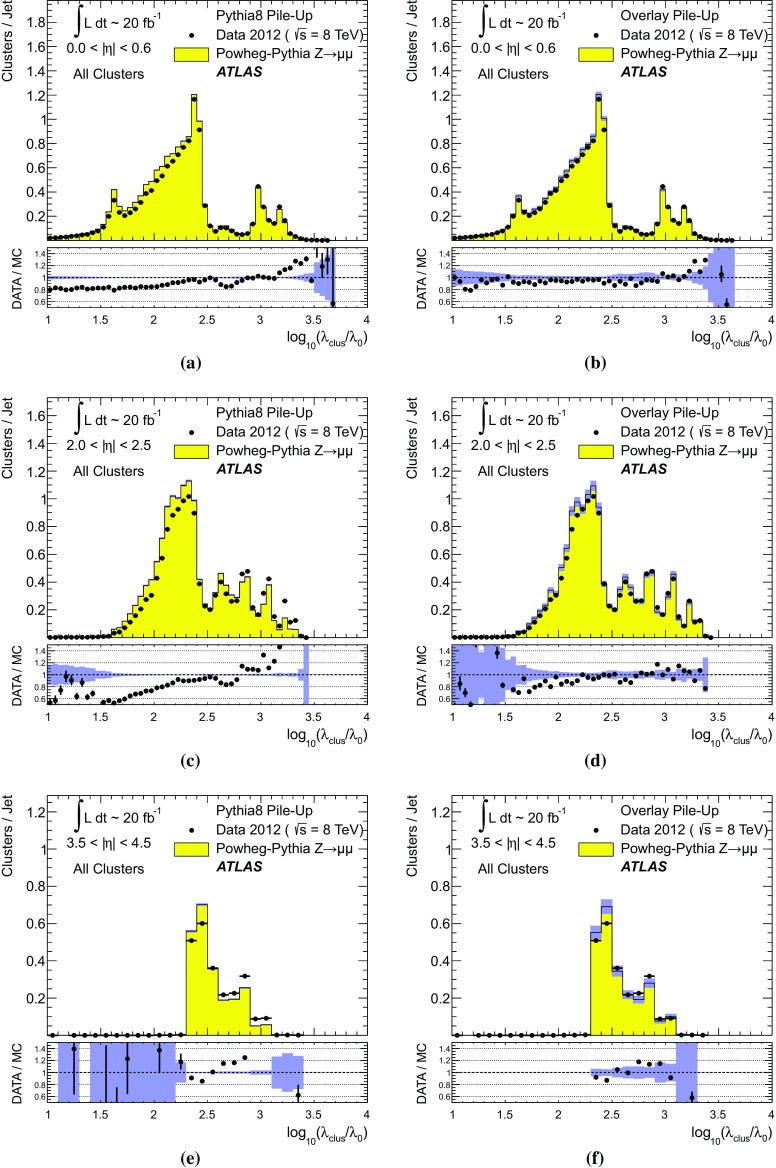
The distribution of the depth location, measured in terms of $\log _{10}(\lambda _{\text {clus}}/\lambda _{0})$ with $\lambda _{0} = 1\ {\text {mm}}$, of all topo-clusters in jets reconstructed with the anti-$k_{t}$ algorithm with $R = 0.4$ and with $30\,{\text {GeV}}< p_{\text {T},\text {jet}}^{{\,{\text {LCW}}+\text {JES}}}< 40\,{\text {GeV}}$ in $Z \!\rightarrow \!\mu \mu$ events in 2012 data and MC simulations with (**a**, **c**, **e**) fully simulated pile-up and with (**b**, **d**, **f**) overlaid pile-up from data. Distributions are shown for jets in the (**a**, **b**) central ($|\eta |<0.6$), the (**c**, **d**) end-cap ($2.0<|\eta |<2.5$), and the (**e**, **f**) forward detector region ($3.5<|\eta |<4.5$). The bin-by-bin ratios of the distributions from data and MC simulations are shown below the plots. The *shaded bands* indicate statistical uncertainties for the distributions from MC simulations and the corresponding uncertainty bands in the ratio plots

**Fig. 35:**
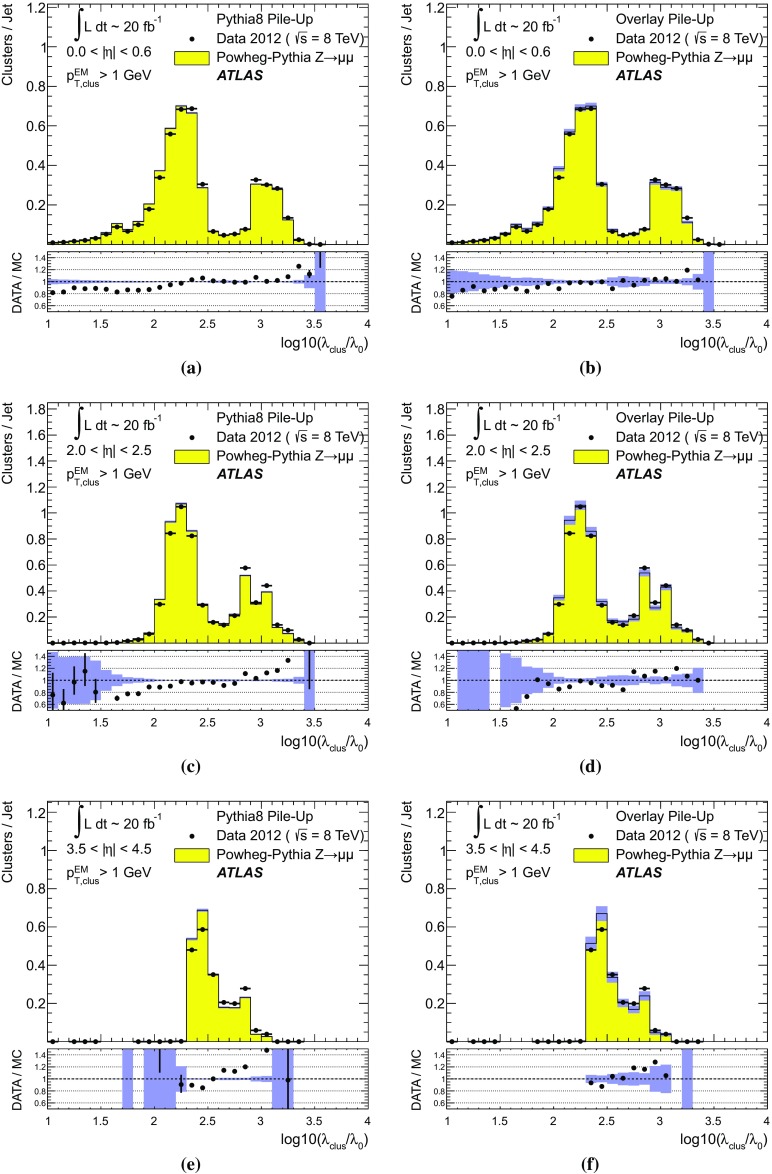
The distribution of the depth location, measured in terms of $\log _{10}(\lambda _{\text {clus}}/\lambda _{0})$ with $\lambda _{0} = 1\,{\text {mm}}$, of topo-clusters with $p_{\text {T},\text {clus}}^{{{\text {EM}}}}> 1\,{\text {GeV}}$ in jets reconstructed with the anti-$k_{t}$ algorithm with $R = 0.4$ and with $30\,{\text {GeV}}< p_{\text {T},\text {jet}}^{{\,{\text {LCW}}+\text {JES}}}< 40\,{\text {GeV}}$ in $Z \!\rightarrow \!\mu \mu$ events in 2012 data and MC simulations with (**a**, **c**, **e**) fully simulated pile-up and with (**b**, **d**, **f**) overlaid pile-up from data. Distributions are shown for jets in the (**a**, **b**) central ($|\eta |<0.6$), the (**c**, **d**) end-cap ($2.0<|\eta |<2.5$), and the (**e**, **f**) forward detector region ($3.5<|\eta |<4.5$). The data-to-MC simulation ratios are shown below the distributions. The *shaded bands* shown for the distributions obtained from MC simulations indicate statistical uncertainties and the corresponding uncertainty bands in the ratio plots

The distribution of the depth location of all topo-clusters inside anti-$k_{t}$ jets reconstructed with $R = 0.4$ and with $30\,{\text {GeV}}< p_{\text {T},\text {jet}}^{{\,{\text {LCW}}+\text {JES}}}< 40\,{\text {GeV}}$ in $Z \!\rightarrow \!\mu \mu$ events in 2012 data and MC simulations is shown in Fig. 34. Like for the depth distribution of topo-clusters in the inclusive $Z \!\rightarrow \!\mu \mu$ sample presented in Fig. 28, the MC simulations with overlaid pile-up data show better agreement with data than the ones with fully simulated pile-up. The differences in the jet context are significantly smaller than observed for the inclusive selection.

Applying a $p_{\text {T},\text {clus}}^{{{\text {EM}}}}> 1\,{\text {GeV}}$ cut to the topo-clusters in the jets results in the depth distributions shown in Fig. 35. This selection also shows better data/MC agreement for the sample with fully simulated pile-up, an indicator consistent with the better simulation of harder signals observed in e.g. Fig. 29. A noticeable difference from the depth distributions obtained from the inclusive sample in Fig. 29a is that for topo-clusters in jets the data/MC agreement in the case of the fully simulated pile-up is already better for the $p_{\text {T},\text {clus}}^{{{\text {EM}}}}> 1\,{\text {GeV}}$ selection, as can be seen in Fig. 35a. In addition, comparing Figs. 34 and 35 shows that the $p_{\text {T},\text {clus}}^{{{\text {EM}}}}> 1\,{\text {GeV}}$ selection predominantly removes topo-clusters at small depth $\lambda _{\text {clus}}$, as the distributions are depopulated more for smaller values of $\lambda _{\text {clus}}$ than for larger ones. This means that mostly topo-clusters generated by soft particles with little penetration depth into the calorimeters, including those consistent with pile-up, are removed. The data/MC comparisons are thus less sensitive to pile-up modelling issues, and therefore show better agreement.

#### Calibration and signal features of the leading topo-cluster

**Fig. 36:**
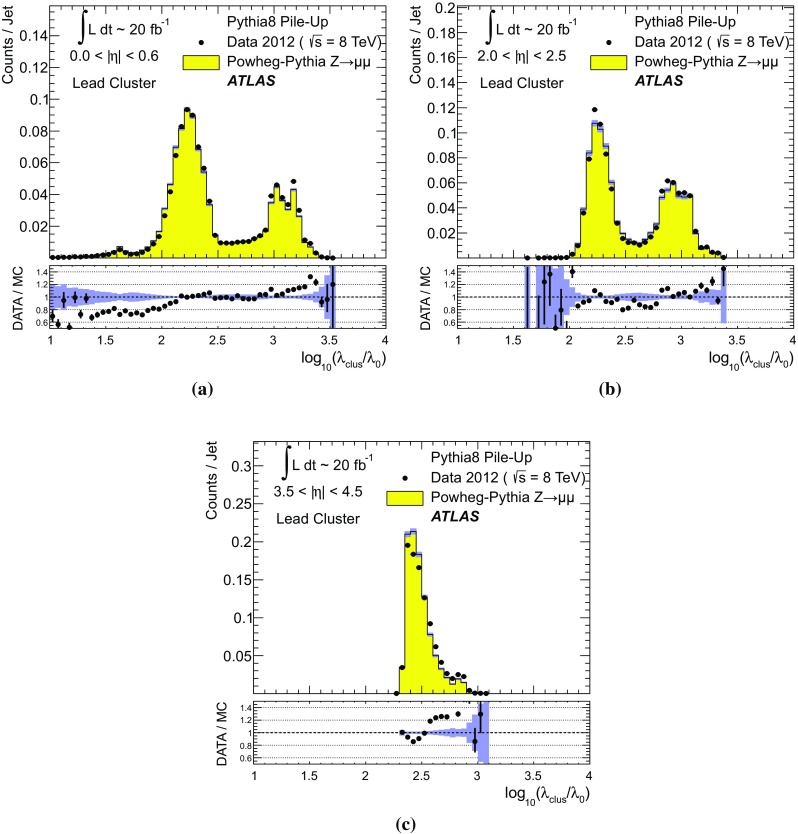
The distribution of the leading topo-cluster depth location measure $\log _{10}(\lambda _{\text {clus}}/\lambda _{0})$ in fully calibrated jets reconstructed with the anti-$k_{t}$ algorithm with $R = 0.4$ and $30\,{\text {GeV}}< p_{\text {T},\text {jet}}^{{\,{\text {LCW}}+\text {JES}}}< 40\,{\text {GeV}}$ in regions of **a** the central ($|\eta _{\text {jet}}|<0.6$), **b** the end-cap ($2.0<|\eta _{\text {jet}}|<2.5$), and the **c** forward ($3.5<|\eta _{\text {jet}}|<4.5$) calorimeters in ATLAS. Data is compared to MC simulations with fully simulated pile-up for $Z \!\rightarrow \!\mu \mu$ events recorded in 2012. The ratio of the distribution from data to the one from MC simulations is shown below each plot. The *shaded bands* show statistical uncertainties for the distributions from MC simulations and the corresponding uncertainty bands in the ratio plots. The reference scale for $\lambda _{\text {clus}}$ is $\lambda _{0} = 1\,{\text {mm}}$

**Fig. 37:**
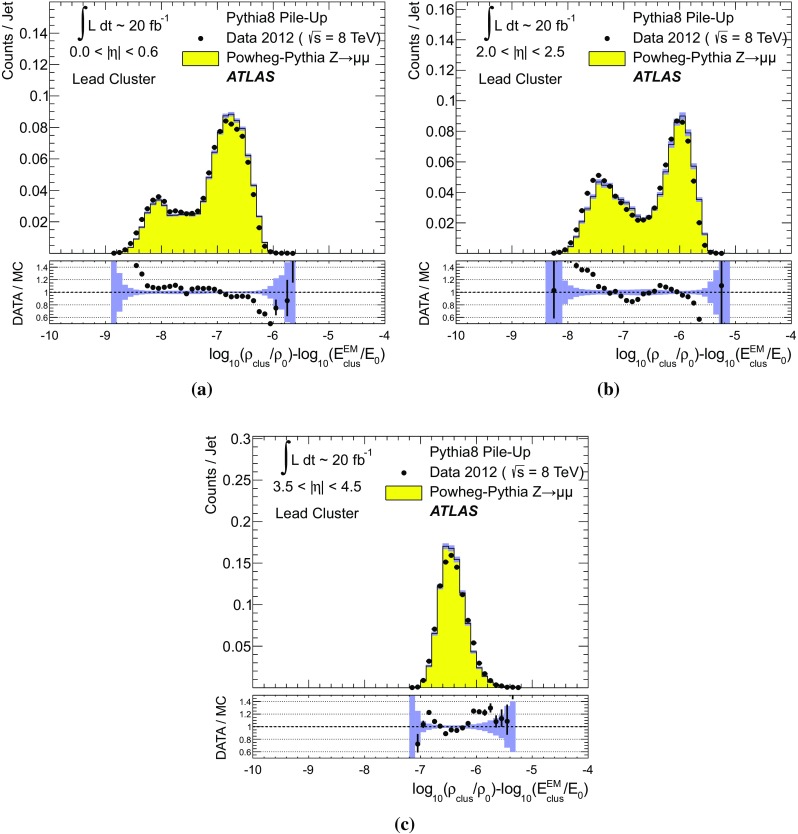
The distribution of the leading topo-cluster signal density measure $\log _{10}(\rho _{\text {clus}}/\rho _{0})-\log _{10}(E_{\text {clus}}^{{{\text {EM}}}}/E_{0})$ in fully calibrated jets reconstructed with the anti-$k_{t}$ algorithm with $R = 0.4$ and $30\,{\text {GeV}}< p_{\text {T},\text {jet}}^{{\,{\text {LCW}}+\text {JES}}}< 40\,{\text {GeV}}$ in regions of **a** the central ($|\eta _{\text {jet}}|<0.6$), **b** the end-cap ($2.0<|\eta _{\text {jet}}|<2.5$), and **c** the forward ($3.5<|\eta _{\text {jet}}|<4.5$) calorimeters in ATLAS. Data is compared to MC simulations with fully simulated pile-up for $Z \!\rightarrow \!\mu \mu$ events recorded in 2012. The ratio of the distribution from data to the one from MC simulations is shown below each plot. The *shaded bands* show statistical uncertainties for the distributions from MC simulations and the corresponding uncertainty bands in the ratio plots. The reference scale for $\rho _{\text {clus}}$ is $\rho _{0} = 1\,\text {MeV}/{\text {mm}}^{3}$, and for the energy $E_{0} = 1\,\text {MeV}$

**Fig. 38:**
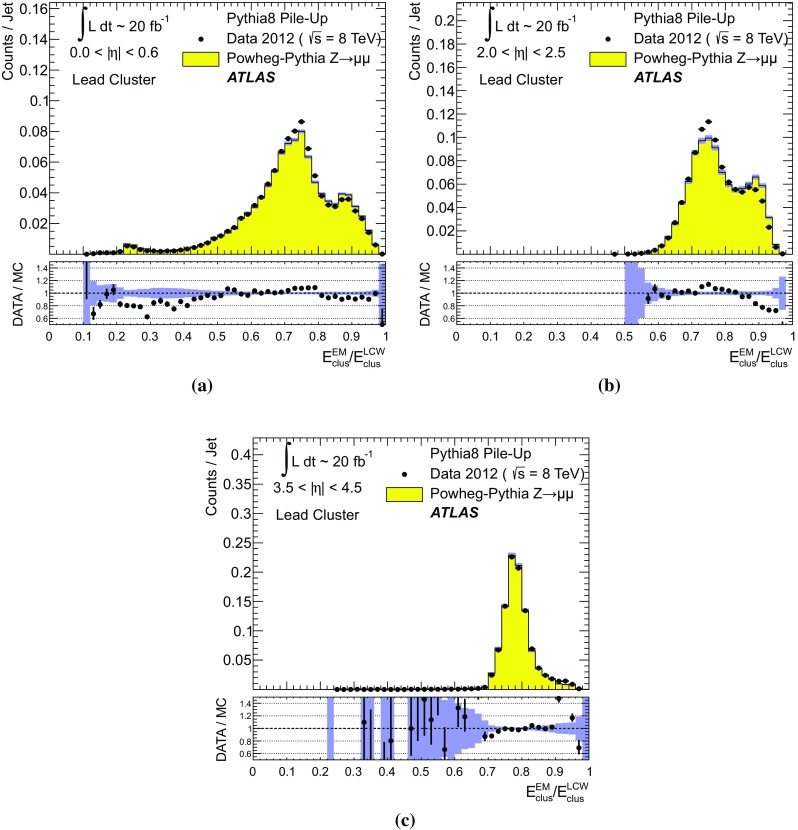
The distribution of the ratio of the cluster signal reconstructed on EM scale $E_{\text {clus}}^{{{\text {EM}}}}$ to the fully calibrated signal $E_{\text {clus}}^{{{\text {LCW}}}}$ for the leading topo-cluster in fully calibrated jets reconstructed with the anti-$k_{t}$ algorithm with $R = 0.4$ and $30\,{\text {GeV}}< p_{\text {T},\text {jet}}^{{\,{\text {LCW}}+\text {JES}}}< 40\,{\text {GeV}}$ in regions of **a** the central ($|\eta _{\text {jet}}|<0.6$), **b** the end-cap ($2.0<|\eta _{\text {jet}}|<2.5$), and **c** the forward ($3.5<|\eta _{\text {jet}}|<4.5$) calorimeters in ATLAS. Data is compared to MC simulations with fully simulated pile-up for $Z \!\rightarrow \!\mu \mu$ events recorded in 2012. The ratio of the distribution from data to the one from MC simulations is shown below each plot. The *shaded bands* show statistical uncertainties for the distributions from MC simulations and the corresponding uncertainty bands in the ratio plots. The reference scale for $\rho _{\text {clus}}$ is $\rho _{0} = 1\,\text {MeV}/{\text {mm}}^{3}$, and for the energy $E_{0} = 1\,\text {MeV}$

The leading topo-cluster in a jet is defined as the one with the highest $p_{\text {T},\text {clus}}^{{{\text {EM}}}}$. Its moments and its signal contribution to the jet provide a good reference for the dependence of important topo-cluster calibration inputs on pile-up. The leading cluster is found in the anti-$k_{t}$ jets reconstructed with $R = 0.4$ and with $30\,{\text {GeV}}< p_{\text {T},\text {jet}}^{{\,{\text {LCW}}+\text {JES}}}< 40\,{\text {GeV}}$ in the 2012 $Z \!\rightarrow \!\mu \mu$ sample in data and MC simulations with full pile-up simulation. The distributions of the topo-cluster moments relevant to the LCW calibration for the leading cluster in the jet are shown in Figs. 36 and 37. The distribution of the overall LCW calibration weight described in Sect. 5.6 is shown in Fig. 38.

The distribution of the depth location of the leading topo-cluster, already discussed for all and selected topo-clusters in the inclusive $Z \!\rightarrow \!\mu \mu$ sample in Sect. 6.2.5 and the $Z \!\rightarrow \!\mu \mu$ sample with jets in Sect. 6.3.3, is shown in Fig. 36a, b, c for jets reconstructed in the central, end-cap, and the forward detector region, respectively. As expected from the previous observations, MC simulations agree reasonably well with data. It is also observed that the leading cluster in the central and end-cap detector regions is most often located either in the electromagnetic or in the hadronic calorimeters, and rarely between the modules. In the forward region, the hardest cluster is most often located in the first FCAL module.

The signal density $\rho _{\text {clus}}$ of topo-clusters is defined in Sect. 4.2.2. Figure 37 shows the $\rho _{\text {clus}}$ distributions for the leading topo-cluster in the jet. The complex structures of these distributions are well modelled. Their shape in the central and end-cap regions is driven by the jet fragmentation. Jets with a leading photon, or two nearby photons from a neutral pion decay, can produce the leading topo-cluster with a high signal density, reflecting the single or the two largely overlapping compact electromagnetic shower(s) reconstructed in the highly granular electromagnetic calorimeters. Jets with a leading hadron that reaches the detector typically produce less dense topo-cluster signals in the corresponding hadronic shower. For these jets an additional geometric effect is introduced, as the leading topo-cluster is more likely located in the hadronic calorimeters in ATLAS.^15^ The typically larger cell sizes in these detectors introduce lower density signals even for compact showers.

The forward detector region has a coarser longitudinal segmentation, with the first module FCAL0 closest to the collision vertex being about $30\,X_{0}$ and $2.5\,\lambda _{\text {nucl}}$ deep [52]. Consequently, most leading topo-clusters in jets going in this direction are located in FCAL0, as can be seen in the $\lambda _{\text {clus}}$ distribution in Fig. 36c. The $\rho _{\text {clus}}$ distribution in Fig. 37c therefore does not show the features seen in Figs. 37a and b, because the calorimeter read-out granularity changes smoothly within this module. The hard transitions between calorimeter modules with very different granularity affecting the $\rho _{\text {clus}}$ distributions in the central and end-cap regions are absent.

**Fig. 39:**
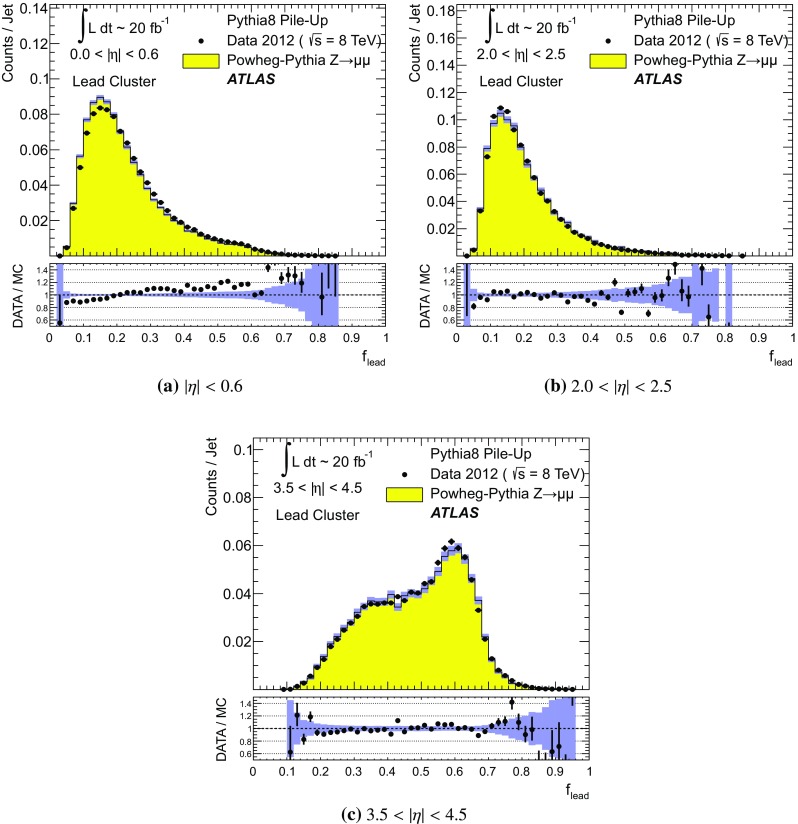
The distribution of the signal fraction $f_{\text {lead}}$ carried by the leading topo-cluster in jets, as defined in Eq. (45), in **a** the central, **b** the end-cap, and **c** the forward detector region. The jets are reconstructed using the anti-$k_{t}$ algorithm with $R = 0.4$ and with $30\,{\text {GeV}}< p_{\text {T},\text {jet}}^{{\,{\text {LCW}}+\text {JES}}}< 40\,{\text {GeV}}$ in $Z \!\rightarrow \!\mu \mu$ events in 2012 data and MC simulations with fully simulated pile-up. The data-to-MC simulation ratios are shown below the distributions. The *shaded bands* shown for the distributions obtained from MC simulations indicate statistical uncertainties and the corresponding uncertainty bands in the ratio plots

The overall effect of the LCW calibration described in Sect. 5 on the signal scale of the leading topo-cluster can be measured by the ratio of the basic EM scale signal $E_{\text {clus}}^{{{\text {EM}}}}$ to the fully calibrated cluster signal $E_{\text {clus}}^{{{\text {LCW}}}}$. The distribution of this ratio is shown for the three detector regions in Fig. 38a. These distributions are inclusive with respect to the topo-cluster classification described in Sect. 5.2. The shapes observed in the central and end-cap detector regions reflect this classification of the leading topo-cluster. The rightmost peak is mostly produced by topo-clusters that are generated by electromagnetic showers and predominantly calibrated as such. In this case the calibration corrections consist of relatively small out-of-cluster and dead material corrections only, as outlined in Sect. 5. As a consequence, $E_{\text {clus}}^{{{\text {EM}}}}/E_{\text {clus}}^{{{\text {LCW}}}}$ is closer to unity. Topo-clusters classified as hadronic receive much larger corrections, and are more likely to populate the lower side of the $E_{\text {clus}}^{{{\text {EM}}}}/E_{\text {clus}}^{{{\text {LCW}}}}$ spectrum.

The $E_{\text {clus}}^{{{\text {EM}}}}/E_{\text {clus}}^{{{\text {LCW}}}}$ distribution in the forward detector region shown in Fig. 38c does not display these shapes. This is due to a lack of classification power in the coarse geometry of the FCAL. Here most topo-clusters are classified as hadronic and receive relatively large corrections. The populated ranges of $E_{\text {clus}}^{{{\text {EM}}}}/E_{\text {clus}}^{{{\text {LCW}}}}$ in Figs. 38a and b indicate that the magnitude of the total correction scaling the basic cluster signal $E_{\text {clus}}^{{{\text {EM}}}}$ up to the locally calibrated signal $E_{\text {clus}}^{{{\text {LCW}}}}$ reaches considerably higher values in the central region than in the end-cap detector regions. This reflects the fact that the incoming particle energies are higher at larger $|\eta |$ for a given range in jet $p_{\text {T}}$. Therefore, the calorimeter response to hadrons relative to the response to electrons and photons ($e/\pi$) rises with increasing $|\eta |$, and reduces the amount of corrections needed. This effect is initially expected to be observed when comparing the end-cap with the forward region displayed in Fig. 38c as well, yet in the FCAL the out-of-cluster and dead material corrections are larger than the hadronic calibration addressing $e/\pi >1$ and thus dominate the overall LCW calibration.

The signal fraction carried by the leading topo-cluster in the jet is calculated relative to the fully corrected and calibrated $p_{\text {T},\text {jet}}^{{\,{\text {LCW}}+\text {JES}}}$, which provides a stable signal reference in the presence of pile-up (see Fig. 30a),

45 $$\begin{aligned} f_{\text {lead}}= \dfrac{p_{\text {T},\text {clus},\text {lead}}^{{{\text {EM}}}}}{p_{\text {T},\text {jet}}^{{\,{\text {LCW}}+\text {JES}}}}. \end{aligned}$$

This means that $f_{\text {lead}}$ is expected to satisfy $0< f_{\text {lead}}< 1$. Figure 39 shows the distribution of $f_{\text {lead}}$ in the three detector regions. The $f_{\text {lead}}$ distributions in the central region shown in Fig. 39a and the end-cap region shown in Fig. 39b display very similar features and indicate the most probable value^16^ is $f_{\text {lead}}^{\text {mop}}\approx \text {12--15}\,$%. The distribution of $f_{\text {lead}}$ in the forward detector region shown in Fig. 39c displays a significantly different shape introduced by the relatively low topo-cluster multiplicity in jets in this region, as shown in Figs. 31e and f. The peak at $f_{\text {lead}}^{\text {mop}}\approx 60\,$% in this distribution is consistent with jets with $N_{\text {clus}}^{\text {jet}}= 1$, and the leftmost shoulder indicates contributions from jets with $N_{\text {clus}}^{\text {jet}}= 2$, with the region in between populated by jets with $N_{\text {clus}}^{\text {jet}}> 2$. All distributions of $f_{\text {lead}}$ are modelled well in the MC simulations with fully simulated pile-up.

#### Pile-up dependence of leading topo-cluster signal features

**Fig. 40:**
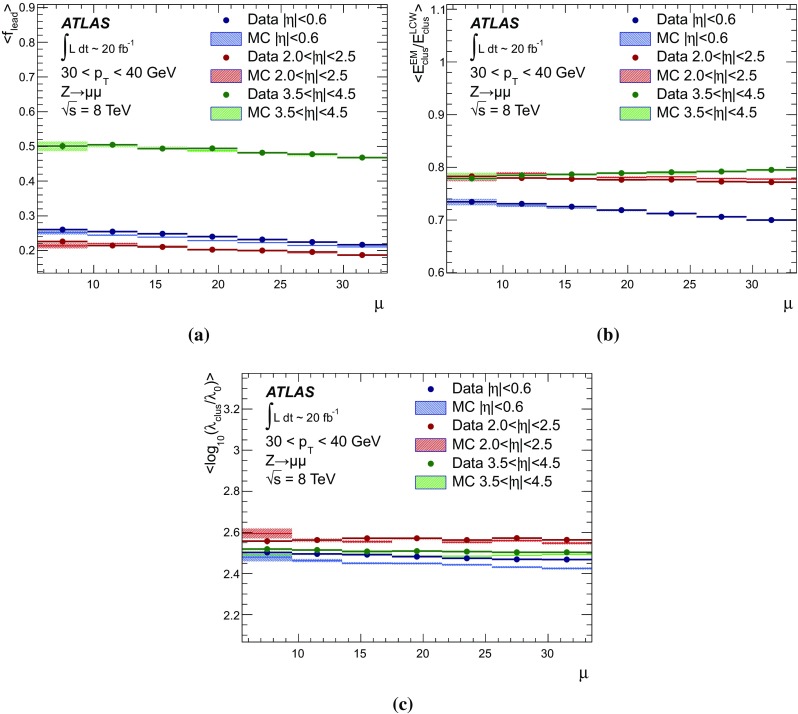
The pile-up dependence of **a** $f_{\text {lead}}$ defined in Eq. (45), **b** $E_{\text {clus}}^{{{\text {EM}}}}/E_{\text {clus}}^{{{\text {LCW}}}}$, and **c** the depth location $\lambda _{\text {clus}}$ of the leading topo-cluster in fully calibrated anti-$k_{t}$ jets reconstructed with $R = 0.4$ and with $30\,{\text {GeV}}< p_{\text {T},\text {jet}}^{{\,{\text {LCW}}+\text {JES}}}< 40\,{\text {GeV}}$ in $Z \!\rightarrow \!\mu \mu$ events in 2012 data and MC simulations with fully simulated pile-up. The reference scale for $\lambda _{\text {clus}}$ is $\lambda _{0} = 1\,{\text {mm}}$. The pile-up activity is measured in terms of the number of pile-up interactions $\mu$. The *shaded bands* shown for the results obtained from MC simulations indicate statistical uncertainties

The pile-up dependence of the average leading cluster signal fraction $\langle f_{\text {lead}}\rangle$, the average $\langle E_{\text {clus}}^{{{\text {EM}}}}/E_{\text {clus}}^{{{\text {LCW}}}}\rangle$ ratio, and the average depth location of the leading topo-cluster are displayed in Fig. 40. The pile-up activity is measured in terms of $\mu$ for this evaluation. A small linear drop of $\langle f_{\text {lead}}\rangle (\mu )$ is observed for increasing $\mu$ in all three detector regions in Fig. 40a. This signal loss of the leading topo-cluster can arise from two effects. First, the increase of the out-of-time pile-up contributions due to the rising $\mu$ reduces the signal due to the bipolar signal shaping function employed in the ATLAS LAr calorimeters (see discussion in Sect. 2.2.1). Second, the increasing in-time pile-up contributions at higher $\mu$ and the increased noise introduced by more out-of-time pile-up leads to additional splitting in the topo-cluster formation, which can take signal away from the leading cluster in the jets.

Figure 40b shows that the overall LCW calibration applied to the leading topo-cluster, measured by the average ratio $\langle E_{\text {clus}}^{{{\text {EM}}}}/E_{\text {clus}}^{{{\text {LCW}}}}\rangle$, in the end-cap and forward detector regions is stable against increasing pile-up activity. A slight drop can be observed with increasing $\mu$ in the central detector region, which indicates changes in the topo-cluster properties relevant to the LCW calibration introduced by increasing pile-up. One possible reason for that may be effects on the topo-cluster splitting in this region, as pile-up can induce spatial energy distributions leading to modifications in the splitting even for hard signal clusters.^17^ The depth location $\lambda _{\text {clus}}$, which enters the LCW calibration in the classification step discussed in Sect. 5.2, is found to be rather stable against pile-up, as shown in Fig. 40c. The pile-up dependence of the leading topo-cluster features discussed here are found to be well modelled in MC simulations with fully simulated pile-up.

#### Leading topo-cluster geometry and shapes

**Fig. 41:**
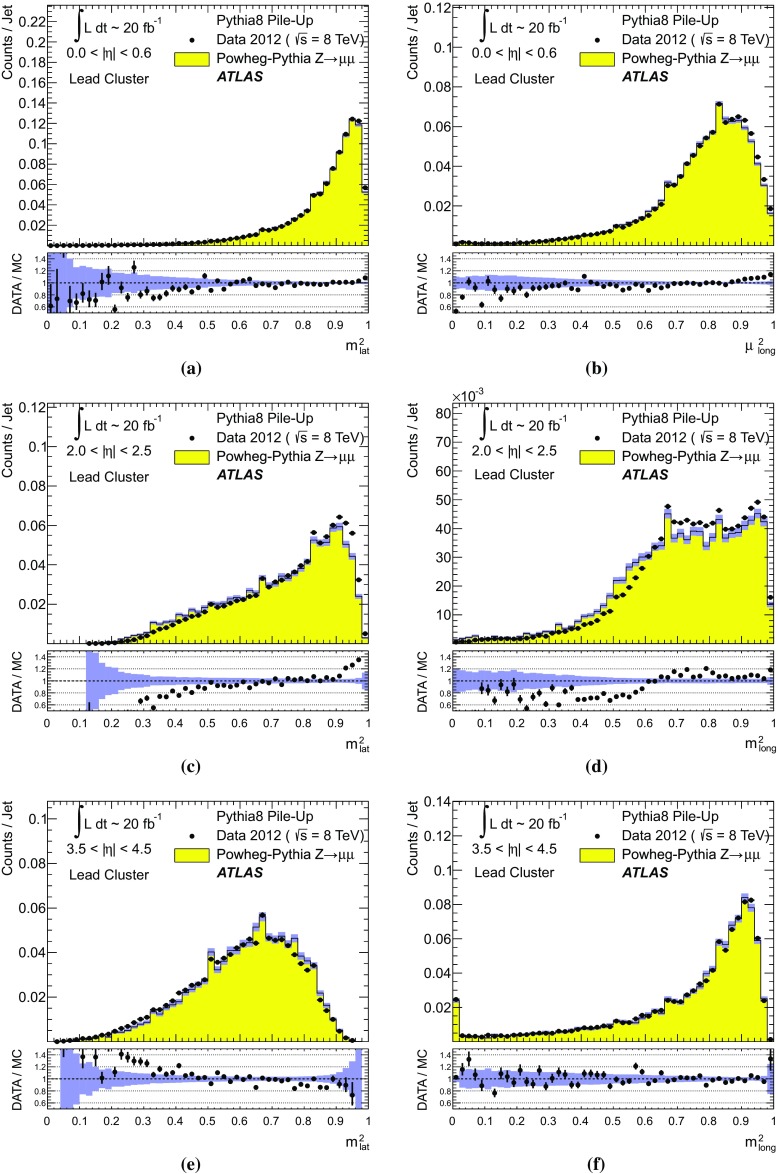
The distribution of the normalised (**a**, **c**, **e**) lateral ($\mathfrak {m}_{\text {lat}}^{2}$) and (**b**, **d**, **f**) longitudinal ($\mathfrak {m}_{\text {long}}^{2}$) extension measures of the leading topo-cluster in fully calibrated anti-$k_{t}$ jets with $R = 0.4$ and $30\,{\text {GeV}}< p_{\text {T},\text {jet}}^{{\,{\text {LCW}}+\text {JES}}}< 40\,{\text {GeV}}$ in $Z \!\rightarrow \!\mu \mu$ events in 2012 data and MC simulations with fully simulated pile-up, for jets in the (**a**, **b**) central ($|\eta |<0.6$), the (**c**, **d**) end-cap ($2.0<|\eta |<2.5$), and the (**e**, **f**) forward detector region ($3.5<|\eta |<4.5$) of ATLAS. The ratios of data and MC simulation distributions are shown below the plots. The *shaded bands* shown for the distributions obtained from MC simulations indicate statistical uncertainties and the corresponding uncertainty bands in the ratio plots

The spatial extensions of the leading topo-cluster in a jet are calculated as described in Sect. 4.1. The distributions of the normalised lateral energy dispersion $\mathfrak {m}_{\text {lat}}^{2}$ given in Eq. (18) and the normalised longitudinal energy dispersion $\mathfrak {m}_{\text {long}}^{2}$ given in Eq. (19) are shown in Fig. 41 for the leading topo-cluster in jets reconstructed with the anti-$k_{t}$ algorithm with $R = 0.4$ and $30\,{\text {GeV}}< p_{\text {T},\text {jet}}^{{\,{\text {LCW}}+\text {JES}}}< 40\,{\text {GeV}}$, in $Z \!\rightarrow \!\mu \mu$ events in 2012 data and MC simulations with fully simulated pile-up. The lateral extensions represented by $\mathfrak {m}_{\text {lat}}^{2}$ are reasonably well modelled in all three detector regions, with some residual discrepancies in particular in the low-value tails and upper edges of the spectra in the end-cap and forward regions. The longitudinal extensions measured by $\mathfrak {m}_{\text {long}}^{2}$ are modelled well in the central and forward detector regions, but their modelling shows some deficiencies in the end-cap region.

**Fig. 42:**
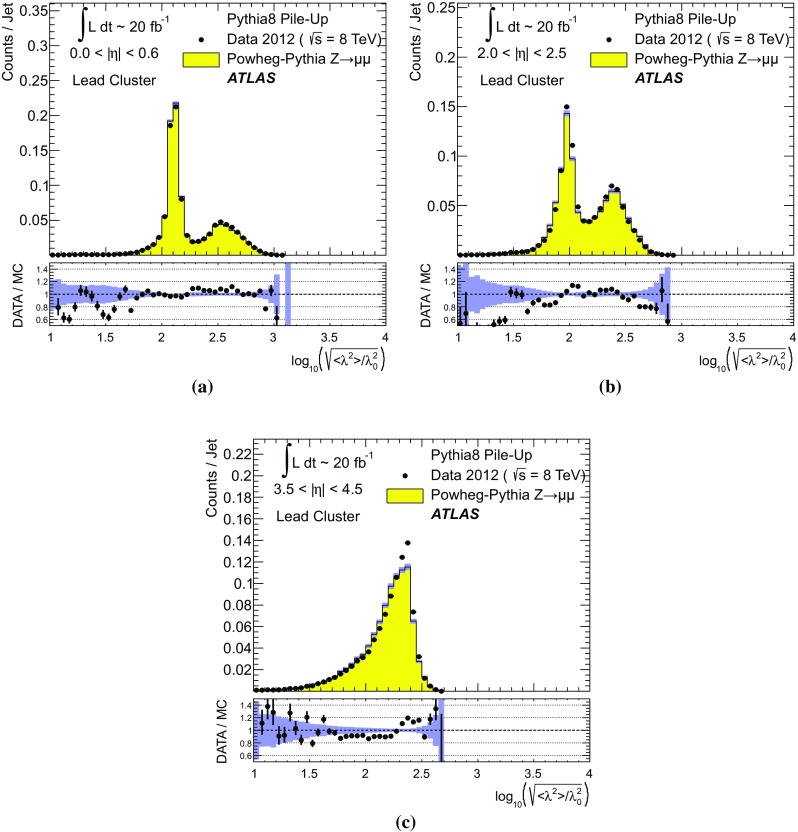
The length of the leading topo-cluster, measured in terms of the longitudinal spread (second moment) $\langle \lambda ^{2}\rangle$ of the cell coordinates along the principal cluster axis by $\sqrt{\langle \lambda ^{2}\rangle /\lambda _{0}^{2}}$, in anti-$k_{t}$ jets reconstructed with $R = 0.4$ and $30\,{\text {GeV}}< p_{\text {T},\text {jet}}^{{\,{\text {LCW}}+\text {JES}}}< 40\,{\text {GeV}}$ in $Z \!\rightarrow \!\mu \mu$ events in 2012 data and MC simulations with fully simulated pile-up. Distributions are shown for jets in the **a** central ($|\eta |<0.6$), the **b** end-cap ($2.0<|\eta |<2.5$), and the **c** forward detector region ($3.5<|\eta |<4.5$). The normalisation of the longitudinal spread is given by $\lambda _{0} = 1\,{\text {mm}}$. The ratios of data-to-MC simulations are shown below the distributions. The *shaded bands* indicate statistical uncertainties of the distributions from MC simulations and the resulting uncertainty bands in the ratio plots

The distribution of the leading topo-cluster length measure $\sqrt{\langle \lambda ^{2}\rangle }$ defined in Sect. 4.1.3 in the three detector regions is shown in Fig. 42a–c. The MC simulations reproduce the shape of the $\sqrt{\langle \lambda ^{2}\rangle }$ distributions from data well in the central and forward regions, with some deficiencies observed in the end-cap region. The shapes in the central and end-cap region are due to leading topo-clusters contained in the electromagnetic calorimeters populating the left peak of the distribution (short clusters) and leading topo-clusters in the hadronic calorimeters populating the right peak with longer clusters. The shape of the length distribution in the forward region shown in Fig. 42c is characterised by a sharp drop on the right of the spectrum, which corresponds to the half-depth of cells ($225\,{\text {mm}}$) in the FCAL modules. This shows that in this detector region the leading topo-cluster rarely extends into all three FCAL modules, as indicated by only few topo-clusters with $\sqrt{\langle \lambda ^{2}\rangle } > 225\,{\text {mm}}$. The leading cluster is more likely to share its energy between the first two modules FCAL0 and FCAL1, with $\sqrt{\langle \lambda ^{2}\rangle } \approx 225\,{\text {mm}}$ indicating a near equal share and $\sqrt{\langle \lambda ^{2}\rangle } < 225\,{\text {mm}}$ indicating that most of the cluster energy is in FCAL0.

**Fig. 43:**
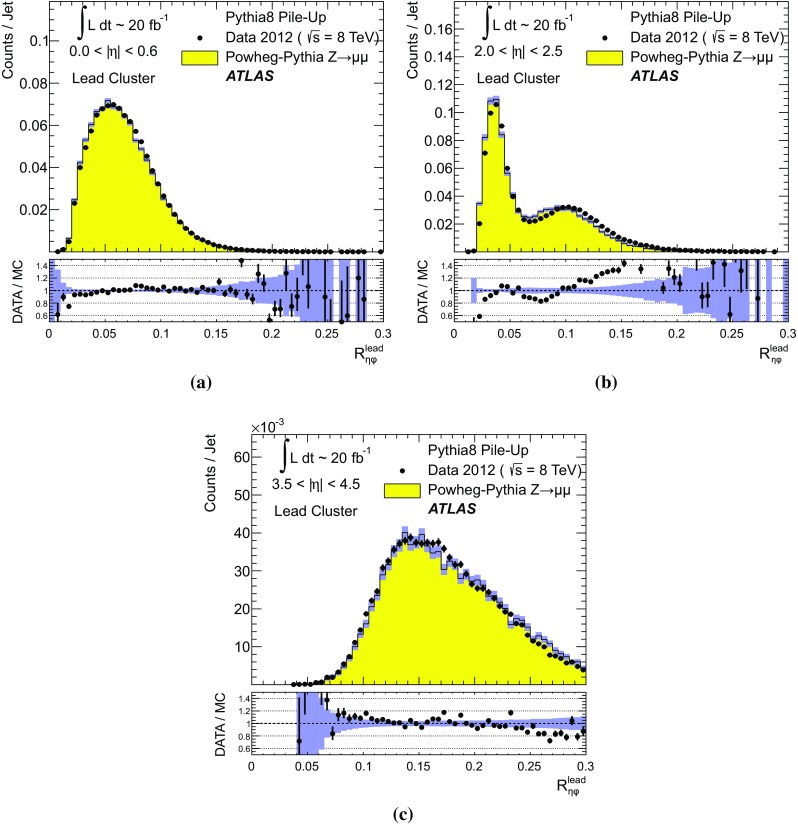
The size $R_{\eta \phi }^{\text {lead}}$ of the leading topo-cluster in $(\eta ,\phi )$ space, measured using Eq. (20), in anti-$k_{t}$ jets reconstructed with $R = 0.4$ and with $30\,{\text {GeV}}< p_{\text {T},\text {jet}}^{{\,{\text {LCW}}+\text {JES}}}< 40\,{\text {GeV}}$ in $Z \!\rightarrow \!\mu \mu$ events in 2012 data and MC simulations with fully simulated pile-up. Distributions are shown for jets in the **a** central ($|\eta |<0.6$), the **b** end-cap ($2.0<|\eta |<2.5$), and the **c** forward detector region ($3.5<|\eta |<4.5$) in ATLAS. The ratios of data to MC simulations are shown below the distributions. The *shaded bands* shown for the distributions obtained from MC simulations indicate statistical uncertainties and the corresponding uncertainty bands in the ratio plots

The size $R_{\eta \phi }^{\text {lead}}$ of the leading topo-cluster in $(\eta ,\phi )$ space is calculated from the respective cluster width estimates $\sigma _{\eta (\phi )}$ given in Eq. (20). Its distributions in various calorimeter regions are shown in Fig. 43. The $R_{\eta \phi }^{\text {lead}}$ distribution in the central region in Fig. 43a is consistent with topo-clusters in a calorimeter with a fine and regular read-out granularity. The double-peak structure in the end-cap region in Fig. 43b shows contributions from leading topo-clusters extending beyond $|\eta | = |\eta _{\text {jet}}| = 2.5$, where the cell granularity drops sharply by about a factor of four. This generates the right peak in the distribution.^18^ The $R_{\eta \phi }^{\text {lead}}$ distribution in the forward detector region displayed in Fig. 43c is consistent with a non-pointing calorimeter read-out segmentation with smooth transitions in the granularity from about $\Delta \eta \times \Delta \phi \approx 0.15\times 0.15$ at $|\eta | = 3.5$ to $\Delta \eta \times \Delta \phi \approx 0.3\times 0.3$ for $|\eta | = 4.5$.

**Fig. 44:**
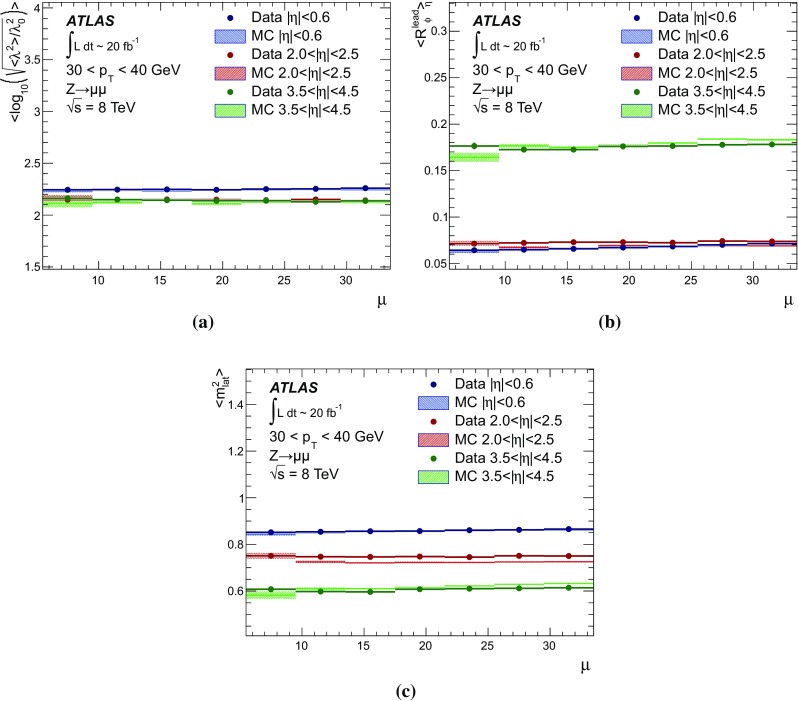
The average pile-up dependence of various geometric observables reconstructed from the leading topo-cluster in anti-$k_{t}$ jets reconstructed with $R = 0.4$ and $30\,{\text {GeV}}< p_{\text {T},\text {jet}}^{{\,{\text {LCW}}+\text {JES}}}< 40\,{\text {GeV}}$ in $Z \!\rightarrow \!\mu \mu$ events in 2012 data and MC simulations with fully simulated pile-up. The average cluster length, represented by $\log _{10}(\langle \lambda ^{2}/\lambda _{0}^{2}\rangle ^{1/2})$ with the reference scale $\lambda _{0} = 1\,{\text {mm}}$, is shown as a function of $\mu$ in **a**, for three detector regions. The average size $\langle R_{\eta \phi }^{\text {lead}}\rangle$ of the leading topo-cluster in $(\eta ,\phi )$ space is displayed for the same detector regions and as a function of $\mu$ in **b**. The average normalised lateral energy dispersion $\langle \mathfrak {m}_{\text {lat}}^{2}\rangle$ of the cluster, as a function of $\mu$ for the three detector regions, is shown in **c**. The *shaded bands* shown around the results obtained from MC simulations indicate statistical uncertainties

#### Pile-up dependence of leading topo-cluster geometry and shapes

The dependence of the geometry and shape of the leading topo-cluster in a jet on the pile-up activity measured by $\mu$ is shown in Fig. 44. No significant dependence is observed for the average longitudinal extension of this cluster shown in Fig. 44a, the average size of this cluster in $(\eta ,\phi )$ space in Fig. 44b, and its average lateral energy dispersion, defined in Eq. (18) and displayed in Fig. 44c.

The data/MC comparison of the average pile-up dependences shows generally acceptable agreement, but also suggests some residual deficiencies likely related to the simulation of the longitudinal and lateral (hadronic) shower shapes. Corresponding observations are reported in Refs. [49, 50, 53, 54] in the context of detailed comparisons of ATLAS test-beam data with simulations.

## Conclusion

Topological cell signal clusters (topo-clusters) provide a well-understood and calibrated signal definition for hadronic final-state reconstruction in the ATLAS calorimeters. The principal algorithm generating these topo-clusters includes a noise-suppression scheme based on signal-significance patterns which is similar to applications in previous experiments. The innovative approach developed for the ATLAS calorimeters not only employs a highly refined implementation of this algorithm in a high-energy, high-luminosity hadron collider environment characterised by significant collision backgrounds introduced by pile-up, but also uses the topo-clusters as a signal base for a local hadronic calibration (LCW) in a non-compensating calorimeter.

Both the topo-cluster formation and the LCW calibration have been validated in collisions without pile-up recorded in 2010, and in the more active pile-up environments observed in 2011 and 2012 operations. The residual effects of pile-up on cluster kinematics and observables in data are well controlled in that they can be reproduced with sufficient precision in MC simulations for topo-clusters either inside or outside jets. The largest observed data–MC differences mainly arise from imperfect modelling of the soft collision physics affecting pile-up. Overlaying pile-up from data on generated hard-scatter interactions in MC simulations yields significantly better agreement for most kinematic variables and topo-cluster moments.

From the LHC Run 1 experience, topo-clusters are now established as a well-performing signal base for jet and transverse missing momentum ($E_{\text {T}}^{\text {miss}}$) reconstruction in ATLAS. They provide noise suppression important for a high-quality calorimeter signal, and in this reduce the amount of data needed to represent the final state in the detector. Their spatial resolution allows not only detailed analysis of the energy flow in the proton–proton collision events as needed for $E_{\text {T}}^{\text {miss}}$ reconstruction but also analysis of more localised energy-flow structures inside jets. This is done routinely in boosted-object reconstruction techniques applied in jet substructure analysis, with recent examples from ATLAS discussed in Refs. [55–58].

## Footnotes

1 ATLAS uses a right-handed coordinate system with its origin at the nominal interaction point (IP) in the centre of the detector and the *z*-axis along the beam pipe. The *x*-axis points from the IP to the centre of the LHC ring, and the *y*-axis points upward. Cylindrical coordinates $(r,\phi )$ are used in the transverse plane, $\phi$ being the azimuthal angle around the beam pipe. The pseudorapidity is defined in terms of the polar angle $\theta$ as $\eta =-\ln \tan (\theta /2)$.

2 Selected examples of the actual distributions taken from data are shown in the context of the topo-cluster formation discussed in Sect. 3.1.1.

3 Stable particles are those with laboratory frame lifetimes $\tau$ defined by $c \tau > 10$ mm.

4 Calorimeter cells marked as having read-out or general signal extraction problems in the actual run conditions are not considered as seeds.

5 Signals in the pre-samplers and gap scintillators are not considered at all in guiding the topo-cluster splitting (see Ref. [1] for a detailed description of the ATLAS calorimeters).

6 This scale is motivated by the Molière radius of the electromagnetic shower, which in good approximation is set to $5\,{\text {cm}}$ for all calorimeters.

7 The first directional moment in $\eta$($\phi$) is only identical to $\eta _{\text {clus}}$($\phi _{\text {clus}}$) for topo-clusters without negative signal cells, because negative signal cells are omitted from its calculation while they contribute to the $\eta _{\text {clus}}$($\phi _{\text {clus}}$) reconstruction.

8 The constant parameters $\lambda _{\text {core}}$ and $r_{\text {core}}$ are introduced to ensure a finite contribution of the highest-energy cells to $\mathfrak {m}_{\text {long}}^{2}$ and $\mathfrak {m}_{\text {lat}}^{2}$, respectively, as those can be very close to the principal shower axes. The specific choices $\lambda _{\text {core}} = 10\,{\text {cm}}$ and $r_{\text {core}} = 4\,{\text {cm}}$ are motivated by the typical length of electromagnetic showers and the typical lateral cell size in the ATLAS electromagnetic calorimeters.

9 The $\sigma _{\eta }$ and $\sigma _{\phi }$ in this equation represent the energy-weighted root mean square (RMS) of the respective cell directions $\eta _{\text {cell}}$ and $\phi _{\text {cell}}$. Correspondingly, the full width at half maximum estimates for the topo-cluster are closer to $2.35 \sigma _{\eta }$ and $2.35 \sigma _{\phi }$.

10 Out-of-time pile-up introduces a coherent component into the calorimeter cell noise due to the correlation of signals in adjacent cells in showers generated by past energy flow. This contribution is reflected on average in the value for $\sigma _{\text {noise,cell}}^{\,{{\text {EM}}}}$, but cannot explicitly be evaluated for any given cell due to its highly stochastic and beam-conditions dependent nature.

11 For the purpose of this calculation, the EMC consists of sampling layers EMB1 to EMB3, EME1 to EME3, and FCAL0.

12 As cells and clusters are localised in the calorimeters, the preferred variables for this space are the azimuth $\phi$ and the pseudorapidity $\eta$, rather than the rapidity *y*. As topo-clusters are reconstructed as massless pseudo-particles (see Sect. 3.2), $y = \eta _{\text {clus}}$ for the complete object.

13 This is because the electromagnetic energy scale reconstructs a signal larger than expected for the deposited energy in case of pure ionisation, due to the lack of showering.

14 These can be generated by particles from different pile-up collisions going in the same direction in a given event.

15 In the case of a leading (stable) hadron in the jet, the leading topo-cluster may still arise from a photon, as the selection of this cluster is performed on the EM scale. This introduces a bias due to $e/\pi >1$, which is nevertheless well modelled in MC simulations, according to Fig. 37.

16 The particular choice of normalisation in the definition of $f_{\text {lead}}$ in Eq. (45) means that even for jets with only one topo-cluster $f_{\text {lead}}^{\text {mop}}$ is expected to be smaller than unity.

17 In particular, pile-up can introduce an additional signal maximum at the boundary of a relatively dense leading topo-cluster, which can have a significant effect on e.g. $\rho _{\text {clus}}$ and other cluster properties pertinent to the LCW calibration.

18 The location of this peak is consistent with the change of the cell size in sampling layers EME1 and EME2 of the electromagnetic end-cap calorimeter at $|\eta | = 2.5$, see Table 1.
